# Mechanistic elucidation guided by covalent inhibitors for the development of anti-diabetic PPARγ ligands†
†Electronic supplementary information (ESI) available: Supplementary figures and tables, experimental methods, procedures for synthesis, and full characterization data of compounds. See DOI: 10.1039/c6sc01279e


**DOI:** 10.1039/c6sc01279e

**Published:** 2016-05-13

**Authors:** Hwan Bae, Jun Young Jang, Sun-Sil Choi, Jae-Jin Lee, Heejun Kim, Ala Jo, Kong-Joo Lee, Jang Hyun Choi, Se Won Suh, Seung Bum Park

**Affiliations:** a Department of Chemistry , Seoul National University , Seoul 151-747 , Korea . Email: sbpark@snu.ac.kr ; Fax: +82 2 884 4025; b Department of Biological Science , Ulsan National Institute of Science and Technology , Ulsan 689-798 , Korea; c Graduate School of Pharmaceutical Sciences and College of Pharmacy , Ewha Womans University , Seoul 120-750 , Korea; d Department of Biophysics and Chemical Biology , Seoul National University , Seoul 151-747 , Korea

## Abstract

Peroxisome proliferator-activated receptor gamma (PPARγ) is a ligand-regulated transcription factor that plays crucial roles in adipogenesis, lipid metabolism, and glucose homeostasis. Several PPARγ ligands possess anti-diabetic activity and they commonly inhibit the phosphorylation of PPARγ at serine 273 (Ser273). The recently reported PPARγ ligand SR1664, which selectively blocks the phosphorylation of PPARγ without classical agonism, has potent anti-diabetic activity, indicating that the inhibition of Ser273 phosphorylation is sufficient to provoke anti-diabetic effects. In this study, we revealed the X-ray structure of PPARγ co-crystallized with SR1664 bound to the alternate binding site of PPARγ and confirmed that the alternate site binding of SR1664 blocks the phosphorylation of Ser273. Furthermore, using covalent inhibitors as chemical tools, we demonstrated that the inhibition of phosphorylation is attributed to the occupation of a specific site which is a hydrophobic region between helix 3 and β3–β4 at the binding pocket of PPARγ. In high-fat diet-induced obese mice, we confirmed the anti-diabetic activity of our covalent inhibitor SB1453 that was designed to bind at the specific site in PPARγ for blocking the phosphorylation of Ser273. Lastly, the target selectivity of SB1453 was demonstrated by fluorescence-based visualization of target proteins complexed with the covalent probe **11** containing a bioorthogonal functional group.

## Abstract 2

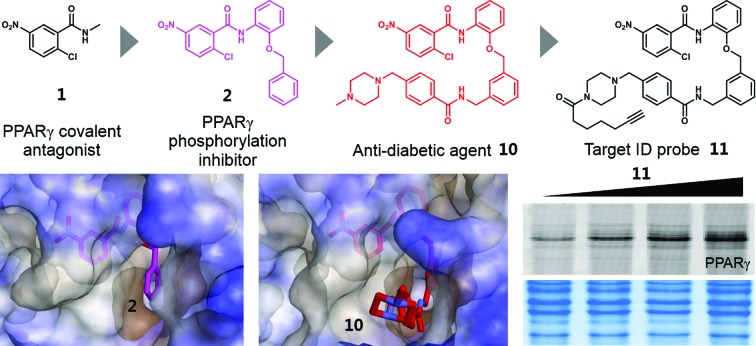
We revealed the X-ray structure of PPARγ co-crystallized with SR1664 bound to the alternate binding site of PPARγ and confirmed that this blocks the phosphorylation of Ser273.

## Introduction

Insulin resistance, a major symptom of type II diabetes, is a condition in which body cells become resistant to the normal actions of insulin.^1^ Because of the potent insulin-sensitizing effects of peroxisome proliferator-activated receptor gamma (PPARγ)-targeting drugs such as rosiglitazone and pioglitazone, PPARγ has been considered to be a major therapeutic target for the treatment of type II diabetes, although the underlying molecular mechanisms are still unclear.^2^–^4^ However, Choi *et al.* proposed a plausible mechanism by which anti-diabetic PPARγ ligands affect insulin sensitivity.^5^ They demonstrated that the obesity-induced phosphorylation of PPARγ at serine 273 (Ser273) results in the dysregulation of a subset of genes involved in insulin resistance and revealed that anti-diabetic PPARγ ligands effectively block this phosphorylation.^5^ Based on these findings, it was suggested that the efficacy of anti-diabetic PPARγ ligands is attributed to their ability to inhibit the phosphorylation of PPARγ. Moreover, it was recently elucidated that an extracellular signal-regulated kinase (ERK)/cyclin-dependent kinase 5 (Cdk5) axis regulates the diabetogenic actions of PPARγ through the phosphorylation of Ser273,^6^ and that Ser273-phosphorylated PPARγ is selectively recognized by thyroid hormone receptor-associated protein 3 (Thrap3) and regulates a diabetic gene set through these signaling pathways.^7^

Although glitazones have remarkable effects in the treatment of type II diabetes, their use has declined because of their serious adverse effects including weight gain, fluid retention, and congestive heart failure.^8^,^9^ Glitazones both inhibit the phosphorylation of Ser273 and fully activate the expression of PPARγ target genes, which is referred to as classical transcriptional agonism.^5^ Full classical agonism has been suspected of causing serious side effects; thus, there have been consistent efforts to develop a selective PPARγ modulator (SPPARγM) that exhibits reduced classical agonism while retaining potent effects on insulin sensitization.^10^–^12^ Because PPARγ has a large binding pocket and multiple interaction points with ligands, it is expected that PPARγ activities can be selectively regulated through the site-specific binding of ligands.^13^ The recent discovery of SR1664 as a representative SPPARγM helped demonstrate that the complete and selective modulation of PPARγ activities is possible *via* specific ligand binding. Unlike glitazones, SR1664, which inhibits the phosphorylation of Ser273 without altering the transcriptional activity of PPARγ, exerts potent *in vivo* anti-diabetic effects without causing fluid retention and weight gain.^14^ In consideration of these results, there is no doubt that designing a selective inhibitor of PPARγ phosphorylation can be a powerful strategy for the development of a novel anti-diabetic agent targeting PPARγ. However, the exact structural mechanism by which anti-diabetic PPARγ ligands block Ser273 phosphorylation has not yet been elucidated.

## Results and discussion

### Alternate site binding of SR1664 blocks phosphorylation of PPARγ at Ser273

**Fig. 1:**
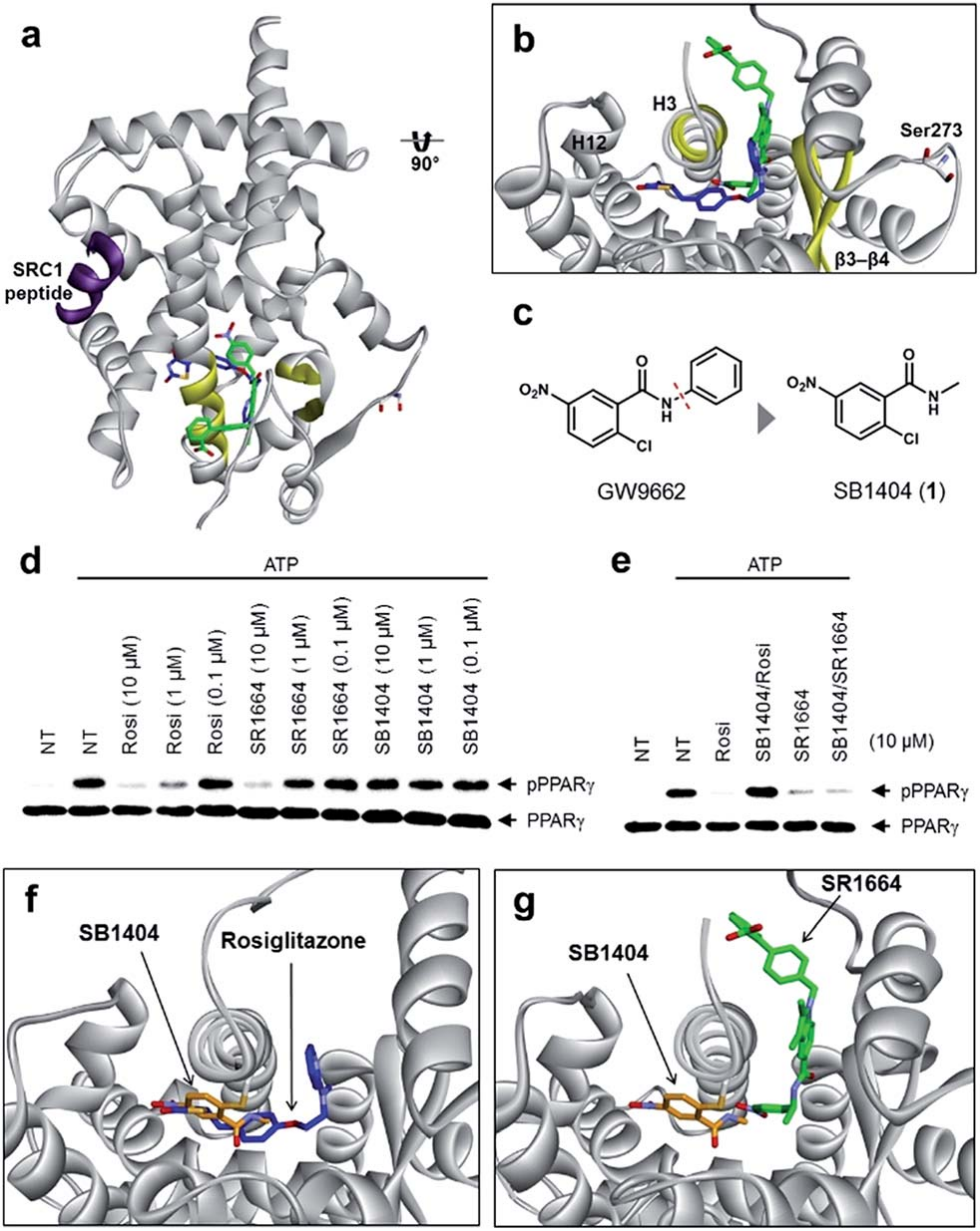
Structural elucidation of the binding mode of SR1664 which blocks PPARγ phosphorylation at Ser273. (a, b) Alignment of the SR1664–PPARγ LBD (green) and rosiglitazone–PPARγ LBD (blue, PDB: 2PRG) X-ray co-crystal structures. (c) Design of a small PPARγ antagonist, SB1404. (d) *In vitro* Cdk5 assay of PPARγ in the presence of rosiglitazone, SR1664 or SB1404. (e) *In vitro* Cdk5 assay on PPARγ treated by rosiglitazone or SR1664 with or without SB1404. NT, no treatment; pPPARγ, phosphorylated PPARγ. (f) Alignment of the rosiglitazone–PPARγ LBD (blue, PDB: ; 2PRG) and SB1404–PPARγ LBD (orange) X-ray co-crystal structures. (g) Alignment of the SR1664–PPARγ LBD (green) and SB1404–PPARγ LBD (orange) X-ray co-crystal structures.

To understand the exact molecular mechanism by which anti-diabetic PPARγ ligands can inhibit Ser273 phosphorylation, we resolved the crystal structure of the PPARγ ligand-binding domain (LBD) complexed with SR1664 and the SRC1 coactivator peptide to a resolution of 2.20 Å (Fig. 1a and S1a†). As the molecular interaction between PPARγ and SR1664 was mainly associated with the inhibition of Ser273 phosphorylation, we might gain more insight into the mechanism by which anti-diabetic PPARγ ligands block the phosphorylation of Ser273, by analyzing the co-crystal structures of the PPARγ LBD and SR1664. SR1664 had a completely different binding mode from full PPARγ agonists such as rosiglitazone which binds at the canonical binding pocket of PPARγ *via* strong hydrogen bonding with helix 12 (Fig. 1b, blue).^16^ In contrast, SR1664 bound to an alternate site which is defined as the region near the entrance of the canonical binding pocket occupied by the second MRL-20 molecule when two molar equivalents of an MRL-20 ligand are bound to PPARγ (Fig. 1b, green).^17^

To determine the phosphorylation-inhibiting effect of SR1664 bound at the alternate site, we investigated using GW9662, a synthetic irreversible PPARγ inhibitor that covalently binds to cysteine 313 (Cys313 in PPARγ2; Cys285 in PPARγ1) on helix 3 (H3).^18^ This covalent inhibitor completely blocks ligand engagement at the canonical binding pocket without fully inhibiting the alternate site binding of PPARγ ligands.^17^ In this study, GW9662 was used to block the canonical binding pocket to determine whether the alternate site binding of SR1664 affects coregulatory interactions.^17^ However, based on the X-ray co-crystal structure, we found that SR1664 exhibited a steric clash with the phenyl group of GW9662 (Fig. S2†), which can interfere in the binding event of SR1664 at the alternate site of PPARγ when this ligand engages PPARγ with the binding mode shown in the crystal structure.

Therefore, we designed and synthesized a smaller covalent inhibitor, SB1404 (**1**), by replacing the phenyl group with a methyl group (Fig. 1c). Compared to rosiglitazone or SR1664, SB1404 did not inhibit the Cdk5-mediated phosphorylation of PPARγ at any concentration *in vitro* (Fig. 1d). Nevertheless, SB1404 completely blocked the inhibitory effect of rosiglitazone on PPARγ phosphorylation, but it did not affect the inhibition of PPARγ phosphorylation by SR1664 (Fig. 1e).

Furthermore, on the basis of the X-ray crystal structure of the PPARγ LBD complexed with SB1404 and the SRC1 coactivator peptide (resolution, 2.80 Å), we confirmed that SB1404 covalently bound to Cys313 on H3 and completely blocked the binding of rosiglitazone at the canonical binding pocket of PPARγ (Fig. 1f and S3†). Unlike GW9662, SB1404 exhibited no steric clash with SR1664; thus, SR1664 can bind to SB1404-labeled PPARγ with the mode shown in the crystal structure (Fig. 1g), indicating that alternate site binding of SR1664 directly inhibits PPARγ phosphorylation. In fact, a different binding mode of SR1664 was recently reported,^15^ but it is not possible for SR1664 to bind to SB1404-labeled PPARγ through the reported binding mode (Fig. S4†). Although more studies are necessary to determine which conformation of ligand binding is the major one, we clearly determined the functional effect of the alternate site binding of SR1664 in terms of phosphorylation inhibition at Ser273 by Cdk5.

### Site-specific binding of PPARγ ligands causes inhibition of the phosphorylation

**Fig. 2:**
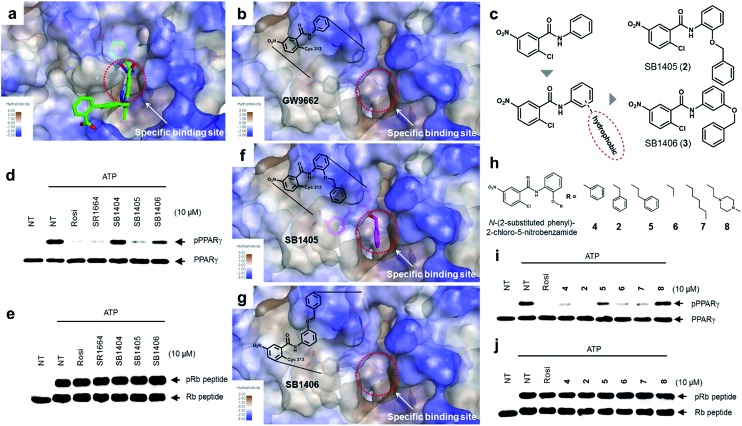
Structure-based rational design of covalent inhibitors of PPARγ phosphorylation. (a) The proposed specific binding site in the SR1664–PPARγ LBD X-ray co-crystal structures (green). (b) The specific binding site on the surface of the PPARγ LBD complexed with GW9662 (black, PDB: 3B0R). (c) Structure-based design of the covalent inhibitors SB1405 and SB1406, which were expected to bind at the specific site. (d, e) *In vitro* Cdk5 assay of PPARγ or the Rb peptide on treatment with rosiglitazone, SR1664, or the covalent inhibitors. NT, no treatment; pPPARγ, phosphorylated PPARγ; pRb peptide, phosphorylated Rb peptide. (f, g) Binding modes of SB1405 (pink) and SB1406 (brown) confirmed by X-ray crystallography. (h) Chemical structures of *N*-(2-substituted phenyl)-2-chloro-5-nitrobenzamides. (i, j) *In vitro* Cdk5 assay of PPARγ or the Rb peptide on treatment with rosiglitazone or *N*-(2-substituted phenyl)-2-chloro-5-nitrobenzamides.

The common biological effect of both SR1664 and rosiglitazone is the specific inhibition of PPARγ phosphorylation at Ser273, which means that they should share a common characteristic.^14^ By analyzing the binding modes of SR1664 and rosiglitazone in X-ray co-crystal structures, we found that both ligands occupied a specific hydrophobic region between helix H3 and the β3–β4 loop (Fig. 2a). Based on this structural insight, we hypothesized that the ligand binding at this specific binding site is responsible for the inhibition of PPARγ phosphorylation at Ser273. To test this hypothesis, we designed two covalent inhibitors that could selectively bind at the alternate site. Considering the structures of GW9662 and SB1404, it was found that a 2-chloro-5-nitrobenzamide moiety can serve as an electrophile and covalently trap Cys313 regardless of the functional groups attached to the amide moiety (Fig. S3†). Therefore, we rationally designed and synthesized SB1405 (**2**) and SB1406 (**3**) containing 2-(benzyloxy)phenyl and 3-(benzyloxy)phenyl groups, respectively, instead of the phenyl group in GW9662 (Fig. 2b and c).

On the basis of the GW9662–PPARγ co-crystal structure, we expected that the additional hydrophobic benzyl moiety would occupy the specific binding site of PPARγ between H3 and β3–β4, and conducted *in vitro* Cdk5 assays using the synthesized covalent inhibitors. Only SB1405 inhibited the phosphorylation of PPARγ (Fig. 2d) without blocking the phosphorylation of the C-terminal fragment of the retinoblastoma protein (Rb peptide), a well-known Cdk5 substrate (Fig. 2e).^19^ This indicated that SB1405 does not affect the fundamental kinase function of Cdk5 but blocks the phosphorylation of PPARγ at Ser273 similar to the effects of rosiglitazone and SR1664. However, we did not observe this inhibitory activity in the case of SB1406, which is the structural isomer of SB1405. To explain this intriguing result, we resolved the crystal structures of the PPARγ LBD complexed either with SB1405 or SB1406 to resolutions of 2.75 or 2.95 Å, respectively. Similarly to SB1404, both compounds covalently bound to Cys313, but they displayed different binding modes (Fig. S5†). In particular, the benzyl group of SB1405 occupied the specific binding site of PPARγ (Fig. 2f), whereas the same moiety of SB1406 did not occupy this region (Fig. 2g). Therefore, these co-crystal structures clearly elucidated why only SB1405 inhibited PPARγ phosphorylation at Ser273, demonstrating that the occupation of the hydrophobic alternate site of PPARγ is essential for the inhibition of PPARγ phosphorylation.

**Fig. 3:**
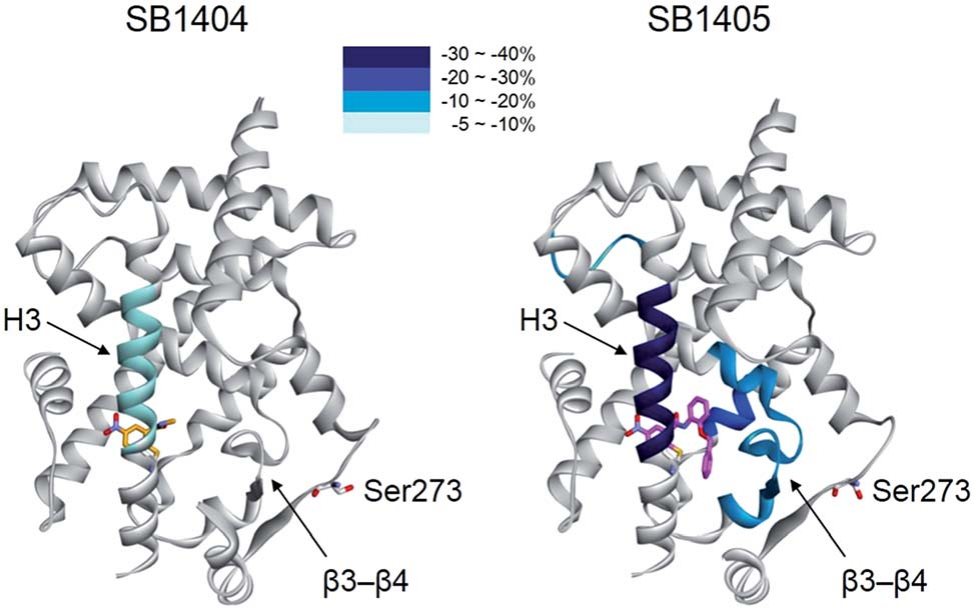
Overlay of differential HDX data onto the structures of the PPARγ LBD bound with either SB1404 or SB1405. Percentage difference in HDX between the apo and ligand-bound receptor is coloured according to the key.

When we aligned the co-crystal structures of the SB1404–PPARγ LBD and SB1405–PPARγ LBD, we did not observe any considerable differences in their backbone conformations with a root-mean-square deviation (RMSD, Cα) of 0.36 Å, and there was no significant difference in the positioning of the residues around the specific binding site (Fig. S6†), which is consistent with previous crystallography study of PPARγ.^20^ Based on this structural information, we assumed that the inhibition of PPARγ phosphorylation is not an outcome of conformational changes but that it is probably caused by ligand-induced changes in the dynamic nature of the PPARγ LBD. To test this hypothesis, we performed a hydrogen/deuterium exchange (HDX) experiment with mass spectrometry. As shown in Fig. 3 and S7,† SB1405, but not SB1404, significantly reduced the hydrogen/deuterium exchange rate in the β-sheet compared to the ligand-free PPARγ LBD. This ligand-induced reduction of the hydrogen/deuterium exchange rate at that site is an indication of change in the dynamic nature of β-sheet, and the reduced flexibility of this region probably results in the subsequent inhibition of Cdk5-mediated PPARγ phosphorylation at Ser273. This concept has been discussed in previous studies based on results that PPARγ phosphorylation inhibitors commonly stabilized H3 and the β-sheet region.^5^,^14^,^17^ However, we clearly demonstrated this concept by comparing the HDX-MS results of SB1404 and SB1405. Moreover, SB1405 did not cause any structural dynamic changes on the C-terminal indicating that SB1405 acts as a partial or non-agonist of PPARγ, while anti-diabetic PPARγ ligands including rosiglitazone decrease hydrogen/deuterium exchange at the C-terminal as well as the β-sheet region of PPARγ in recent HDX studies.^5^

Based on the mechanistic and structural understanding of PPARγ phosphorylation, we selected *N*-(2-substituted phenyl)-2-chloro-5-nitrobenzamide as a suitable molecular framework and synthesized a series of covalent inhibitors of PPARγ phosphorylation containing different R groups to effectively occupy this specific binding site (Fig. 2h). As shown in Fig. 2i, these inhibitors displayed good inhibitory activities toward Cdk5-mediated *in vitro* PPARγ phosphorylation, excluding **8** which contains a hydrophilic piperazine moiety. In contrast, all these compounds exerted no inhibitory effect on the phosphorylation of the Rb peptide by Cdk5 (Fig. 2j). Taken together, the *N*-(2-substituted phenyl)-2-chloro-5-nitrobenz-amides possessing hydrophobic R substituents efficiently blocked Cdk5-mediated PPARγ phosphorylation at Ser273, and the occupation of R substituents at the hydrophobic region between H3 and β3–β4 of PPARγ appears to be sufficient for reducing the flexibility of this region, which inhibits the *in vitro* phosphorylation of PPARγ by Cdk5.

### Structure-guided optimization and evaluation of covalent inhibitors for *in vivo* analysis

**Fig. 4:**
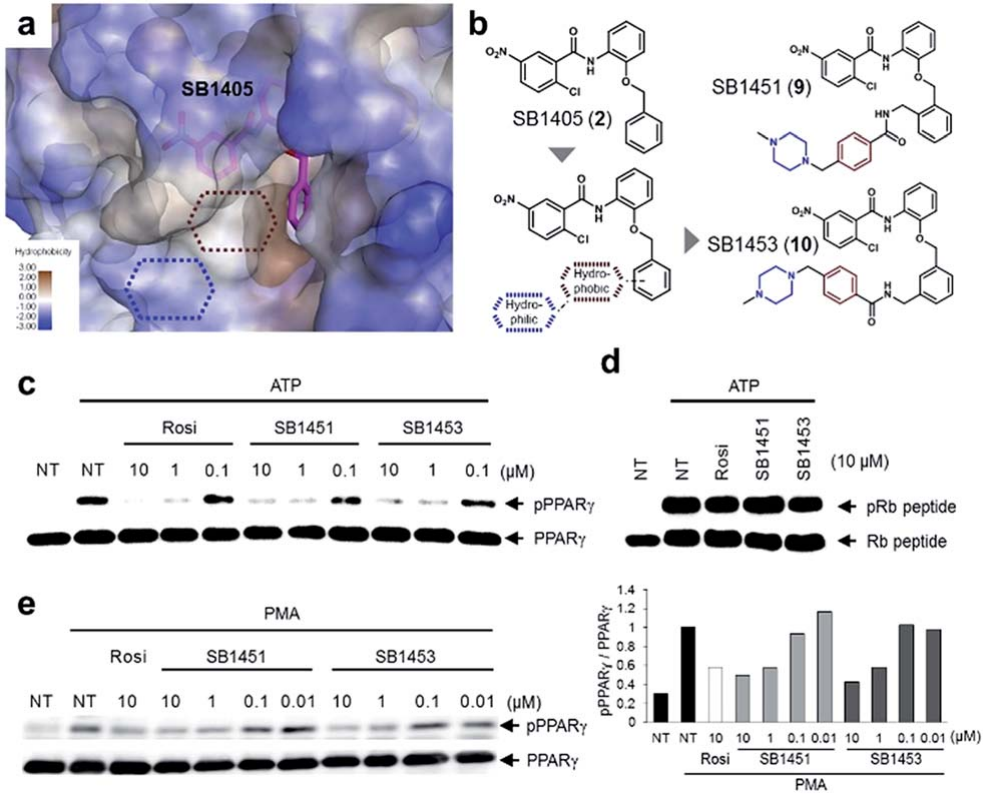
Rational optimization of covalent PPARγ phosphorylation inhibitors for *in vivo* analysis. (a, b) Structure-guided rational design of the covalent inhibitors SB1451 and SB1453 to improve the solubility of SB1405. (c, d) *In vitro* Cdk5 assay of PPARγ or the Rb peptide after treatment with rosiglitazone, SB1451, or SB1453. NT, no treatment; pPPARγ, phosphorylated PPARγ; pRb peptide, phosphorylated Rb peptide. (e) PMA-induced phosphorylation of PPARγ in HEK-293 cells expressing PPARγ after treatment with rosiglitazone, SB1451, or SB1453.

Among the *N*-(2-substituted phenyl)-2-chloro-5-nitrobenzamides, we selected SB1405 (**2**) for a further *in vivo* study because of its excellent inhibition potency in the *in vitro* Cdk5 assay (Fig. 2i) and the perfect occupancy of its benzyl moiety at the specific binding site (Fig. 2f). However, we were concerned that the *in vivo* evaluation of SB1405 might be limited due to its poor solubility. To solve this problem, we decided to modify the structure of SB1405 without changing the 2-chloro-5-nitrobenzamide moiety to preserve covalent anchoring with Cys313 of PPARγ and a hydrophobic benzyl moiety to specifically bind the alternate site for the phosphorylation inhibition. Thus, we introduced an additional moiety on the benzyl moiety of SB1405 to occupy the remaining empty hydrophobic space (brown) and the subsequent hydrophilic space (blue) (Fig. 4a). On the basis of the crystal structure of the SB1405–PPARγ LBD, we designed and synthesized SB1451 (**9**) and SB1453 (**10**) containing hydrophilic piperazine moieties attached to the additional benzene rings to improve solubility (Fig. 4b). After synthesizing SB1451 and SB1453, we examined their inhibitory activities against PPARγ phosphorylation at Ser273 in a concentration-dependent manner and confirmed their excellent potency in the *in vitro* Cdk5 assay (Fig. 4c). This inhibitory potency of SB1451 and SB1453 regarding PPARγ phosphorylation was also conserved in the cellular system, which was similar to that of rosiglitazone (Fig. 4e). However, they did not inhibit the basic kinase function of Cdk5 as monitored by a Western blot analysis of Rb peptide phosphorylation *in vitro* (Fig. 4d).

### Covalent PPARγ phosphorylation inhibitors exert anti-diabetic effects *in vivo* without promoting adipogenesis

**Fig. 5:**
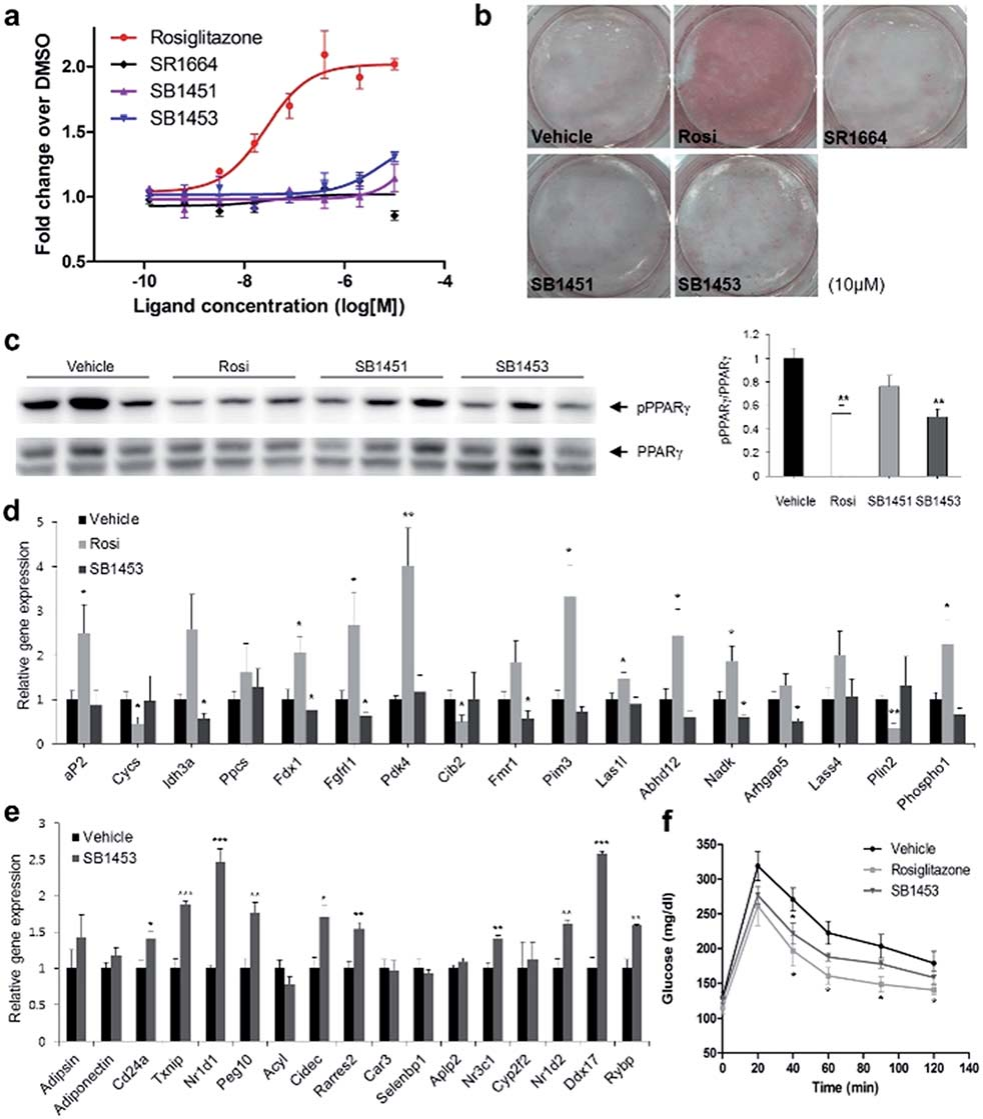
Anti-diabetic activity of SB1453 in DIO mice without promoting adipogenesis. (a) Transcriptional activity of a PPAR-derived reporter gene in HEK-293T cells expressing full-length PPARγ after 24 h of treatment with rosiglitazone, SR1664, SB1451, or SB1453 (*n* = 3). (b) Oil Red O staining of accumulated lipids in 3T3-L1 cells treated with rosiglitazone, SR1664, SB1451, or SB1453. (c) Phosphorylation of PPARγ in the WAT of DIO mice after 7 days of treatment with vehicle, rosiglitazone, SB1451, or SB1453 (10 mg per kg per day). (d) Expression of the agonist gene set in WAT. (e) Expression of the gene set regulated by PPARγ phosphorylation in WAT. (f) Glucose-tolerance test in DIO mice treated with vehicle, rosiglitazone, or SB1453 (7 days, 10 mg per kg per day) (*n* = 6). Error bars denote SEM. *P < 0.05, **P < 0.01, ***P < 0.001.

To examine the agonistic activity in PPARγ-mediated transcription, we conducted two cellular assays. First, using a luciferase reporter gene assay in HEK-293T cells expressing full-length PPARγ with tandem PPAR response elements (PPRE), we confirmed that SB1451 and SB1453 had almost no classical transcriptional agonism compared with that of rosiglitazone (Fig. 5a). Secondly, we tested whether these covalent inhibitors stimulate adipogenesis by monitoring cellular lipid accumulation in 3T3-L1 cells *via* Oil Red O staining.^21^,^22^ As shown in Fig. 5b, rosiglitazone (10 μM) fully stimulated adipocyte differentiation, whereas SB1451 or SB1453 (10 μM) did not trigger adipogenesis in 3T3-L1 cells, which confirms the lack of transactivation associated with these inhibitors. Although we observed a slight increase in the PPARγ transcriptional activity upon treatment with SB1453 at 10 μM in the luciferase assay, this concentration was insufficient to stimulate the differentiation of pre-adipocytes into mature adipocytes.

We evaluated the anti-diabetic activity of SB1451 and SB1453 in animal models using DIO mice that are insulin-resistant with an increased level of phosphorylated PPARγ at Ser273.^5^ All animal experiments were performed according to procedures approved by Ulsan National Institute of Science and Technology’s Institutional Animal Care and Use Committee. As shown in Fig. 5c, SB1453 effectively decreased the phosphorylation of PPARγ at Ser273 in the white adipose tissue (WAT) of the DIO mice similarly to the effect of rosiglitazone. SB1451 was less potent in both the *in vivo* reduction of PPARγ phosphorylation and the resulting anti-diabetic activity than SB1453. Previous studies clearly demonstrated that SR1664, a selective inhibitor of PPARγ phosphorylation without classical agonism, exerts *in vivo* anti-diabetic effects and causes changes in the expression of diabetic genes that were dysregulated as a result of PPARγ phosphorylation in obese animals.^12^,^14^ Similarly to SR1664, SB1453 altered the expression of 10 out of 17 affected genes (Fig. 5e). Furthermore, we did not observe any SB1453-induced stimulation of the “agonist” gene set in the white adipose tissue of the DIO mice as defined in a previous report^14^ (Fig. 5d). The glucose tolerance in the DIO mice was improved with the treatments of SB1453 at 10 mg per kg per day for 7 days, although this effect was moderate compared with that of rosiglitazone (Fig. 5f). These results indicated that SB1453 has anti-diabetic actions and preferentially regulates genes sensitive to PPARγ phosphorylation.

We also investigated several adverse effects, including fluid retention and cardiac hypertrophy, which have been observed following treatment with glitazones.^8^,^23^ As shown in Fig. S8a,† the treatment of rosiglitazone caused hemodilution, whereas the treatment of SB1453 had no detectable changes compared with vehicle. Furthermore, the expressions of natriuretic peptide type B (BNP), the marker gene of heart failure, or myosin heavy chain β (β-MHC), the marker gene of hypertrophy, were significantly increased in only rosiglitazone-treated mice without changes in the heart weight (Fig. S8b and c†). These results strongly suggest that SB1453 does not induce the adverse effects associated with the *in vivo* treatment of glitazones.

### Covalent inhibitor selectively binds to PPARγ

**Fig. 6:**
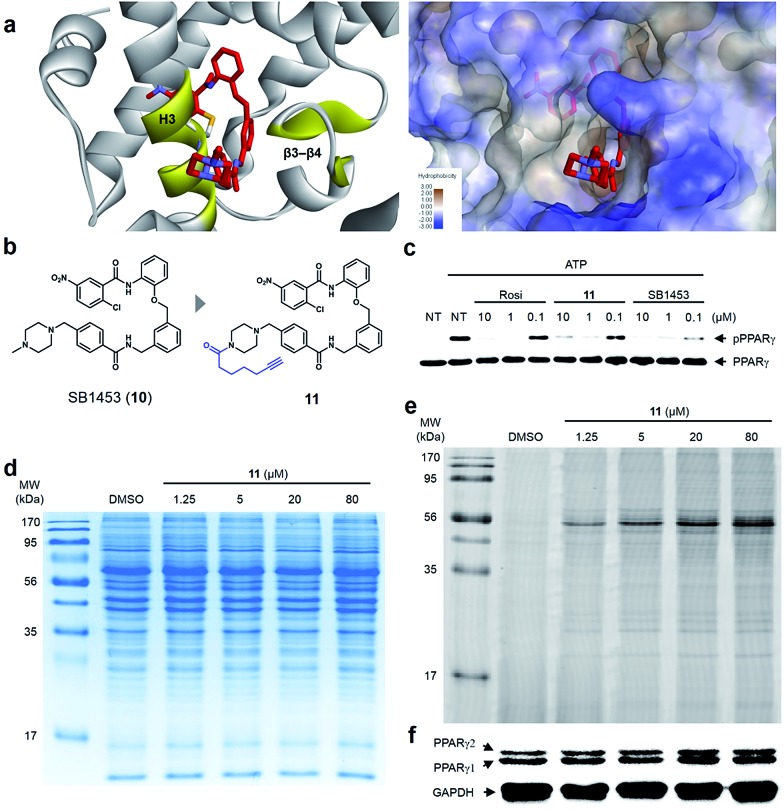
(a) X-ray co-crystal structure of the SB1453–PPARγ LBD reveals the exposure of the piperidine moiety at the entrance of the PPARγ binding pocket. (b) Structure-guided rational design of the target identification probe **11** to demonstrate the target selectivity. (c) *In vitro* Cdk5 assay of PPARγ on treatment with rosiglitazone, **11**, or SB1453 in a dose-dependent manner. (d, e) Coomassie staining and fluorescence scanning patterns of SDS-PAGE of lysates from differentiated 3T3-L1 adipose cells treated with probe **11** in various concentrations. (f) Western blot against the same gel in (e) to confirm PPARγ as the target proteins labeled by **11**.

Lastly, we confirmed the target selectivity of SB1453 using a target identification probe. Prior to the probe design, we resolved the crystal structure of the PPARγ LBD complexed with SB1453 and the SRC1 coactivator peptide (resolution, 2.30 Å) to gain more information about its binding mode (Fig. 6a). As expected, SB1453 covalently bound to Cys313 on H3 and occupied the hydrophobic region between H3 and β3–β4. In addition, its piperazine moiety was positioned at the entrance of the PPARγ binding pocket. Based on this structural information, we designed the target identification probe **11** containing a terminal acetylene group on the piperazine moiety to enable the fluorescence labeling of target proteins *via* a bioorthogonal click reaction (Fig. 6b). Despite the structural change, probe **11** retained its phosphorylation inhibitory effect, indicating that this probe probably covalent bonded with Cys313 on the H3 of PPARγ (Fig. 6c).

First, we confirmed the PPAR subtype selectivity of our covalent inhibitor. Probe **11** was incubated with the proteome of HEK-293T cells expressing either murine PPARα, PPARδ, or PPARγ, followed by a copper-catalyzed click reaction with an azide-containing Cy5 to visualize the proteins complexed with probe **11**. The resulting proteome was separated by gel electrophoresis, and target proteins were visualized *via* fluorescence scanning. Predominantly, the fluorescence-labeled protein was only detected in the PPARγ-transfected cells, indicating that probe **11** efficiently binds to PPARγ, and not to PPARα or PPARδ (Fig. S9†). Then, we performed the same experiment with differentiated 3T3-L1 adipose cells. Interestingly, the predominant bands appeared on the fluorescent gel in a dose-dependent manner without any difference in protein expression pattern (Fig. 6d and e). These labeled proteins were identified as PPARγ1 (53 kDa) and PPARγ2 (57 kDa) by Western blot analysis (Fig. 6f). In fact, they are two isoforms of PPARγ and have a common LBD to which SB1453 binds. This result demonstrated that our covalent inhibitor SB1453 selectively binds to target protein and might be free from general concerns regarding the nonspecific binding of irreversible covalent inhibitors.^24^

## Conclusions

In this study, we identified that an alternate site binding of SR1664 effectively blocks the phosphorylation of PPARγ at Ser273. By comparing this binding mode with that of the conventional PPARγ ligand rosiglitazone, we found that ligand binding at a specific binding site, the hydrophobic region between H3 and β3–β4, is closely related to the inhibition of PPARγ phosphorylation at Ser273. To determine the functional effect of PPARγ ligand binding at the specific site, we rationally designed and synthesized irreversible covalent inhibitors as chemical tools. By analysing the data from biochemical assays, X-ray crystallography, and HDX mass spectrometry with these covalent inhibitors, we found that occupation of the specific binding site in PPARγ by small-molecule ligands causes the change in the dynamic nature of β-sheet and directly correlates with the inhibitory effects on the phosphorylation of PPARγ at Ser273. This structural insight led us to rationally design the improved covalent inhibitors SB1451 and SB1453, which effectively inhibit the phosphorylation of PPARγ at Ser273 *in vitro* and in adipose cells. We also demonstrated that SB1453 exerts potent anti-diabetic effects in DIO mice by blocking PPARγ phosphorylation at Ser273 in the white adipose tissue without several of the adverse effects associated with glitazones. Lastly, through fluorescence-based visualization of the target proteins complexed with the covalent probe **11** containing a bioorthogonal functional handle, we confirmed that our potent anti-diabetic agent SB1453 selectively binds to PPARγ. This study provides a useful guideline for the structure-based rational design of SPPARγMs that effectively inhibit the phosphorylation of Ser273 for the development of anti-diabetic PPARγ ligands. It can be expected that new classes of PPARγ-targeting anti-diabetic drugs will be developed in accordance with this guideline.

## Supplementary Material

Supplementary information

Supplementary information
