# Supplementary material for: Mechanistic elucidation guided by covalent inhibitors for the development of anti-diabetic PPARγ ligands
Source: Chem Sci. 2016 May 13;7(8):5523–9. doi: 10.1039/c6sc01279e (PMC6021786; doi:10.1039/c6sc01279e)

Electronic Supplementary Material (ESI) for Chemical Science.
This journal is © The Royal Society of Chemistry 2016

<Supporting Information>

Mechanistic Elucidation Guided by Covalent Inhibitors for the Development of Anti-diabetic PPARγ Ligands

Hwan Bae,[a] Jun Young Jang,[a] Sun-Sil Choi,[b] Jae-Jin Lee,[c] Heejun Kim,[a] Ala Jo,[a]

Kong-Joo Lee,[c] Jang Hyun Choi,[b] Se Won Suh,[a,d] and Seung Bum Park*[a,d]

[a]Department of Chemistry, Seoul National University, Seoul, 151-747, Korea

[b]Department of Biological Science, Ulsan National Institute of Science and Technology, Ulsan, 689-798, Korea

[c]Graduate School of Pharmaceutical Sciences and College of Pharmacy, Ewha Womans University, Seoul 120-750, Korea

[d]Department of Biophysics and Chemical Biology, Seoul National University, Seoul, 151-747, Korea

*e-mail: sbpark@snu.ac.kr

Content

1. Supplementary Figures……………………………………………………………..**S2**

2. Supplementary Tables……………………………………………………………**S11**

3. Biochemical Procedures…………………………………………………………**S14**

4. Crystallography…………………………………………………………………**S18**

5. *In vivo* studies…………………………………………………………………**S20**

6. Chemical Synthesis……………………………………………………………..**S21**

7. Supplementary References………………………………………………………**S41**

8. ^{1}H and ^{13}C NMR spectra…..…………………………………………………**S42**

1. Supplementary Figures

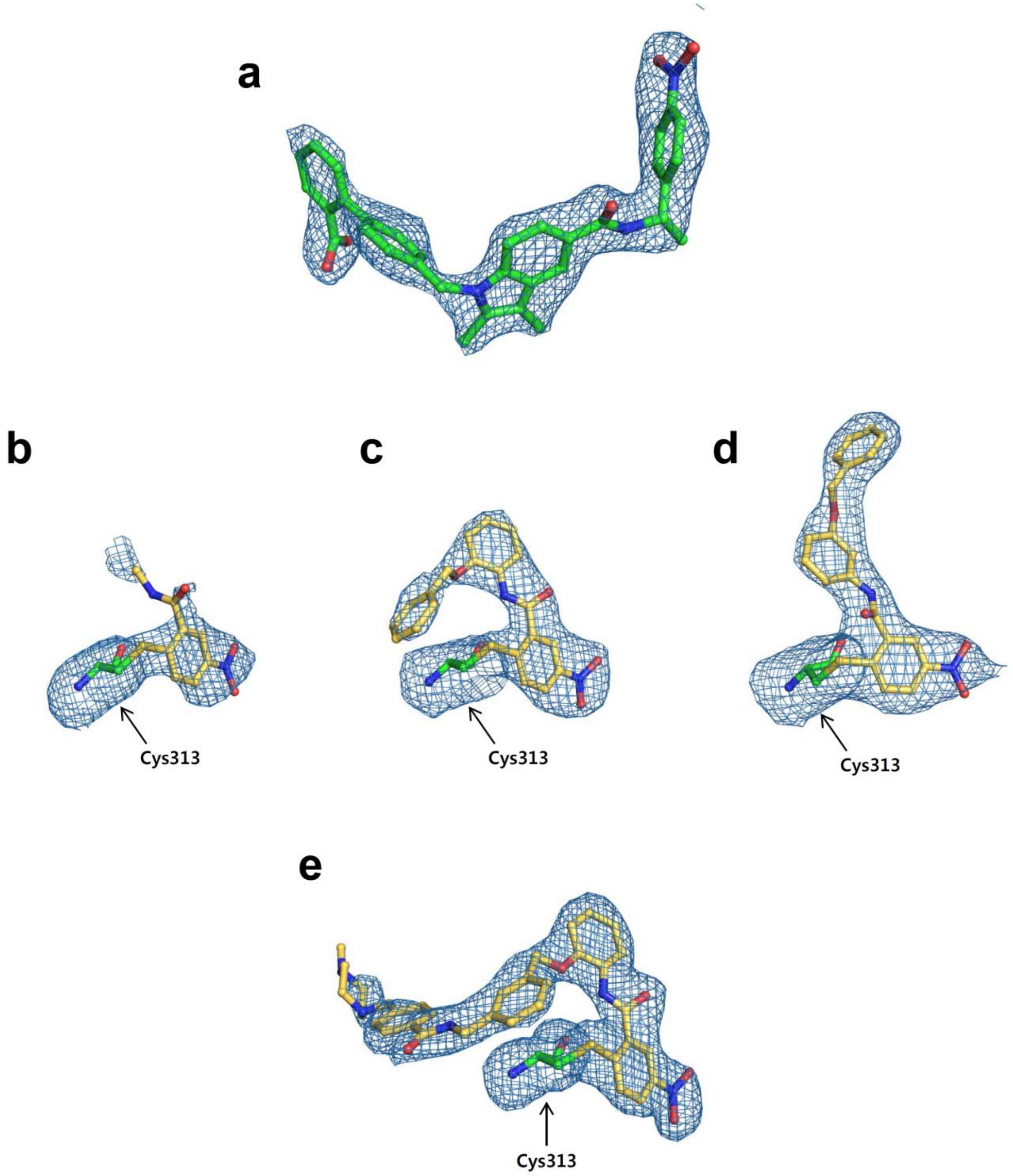

Fig. S1. Electron density maps for compounds. Electron density is shown from the F_O-F_C omit maps (contoured at 2.5σ). (**a**) SR1664 (PDB: 5DWL). (**b**) SB1404 (PDB: 5DV6). (**c**) SB1405 (PDB: 5DV3). (**d**) SB1406 (PDB: 5DSH). (**e**) SB1453 (PDB: 5DVC).

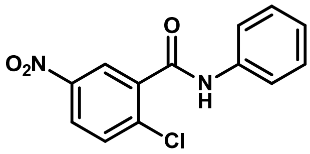

GW9662

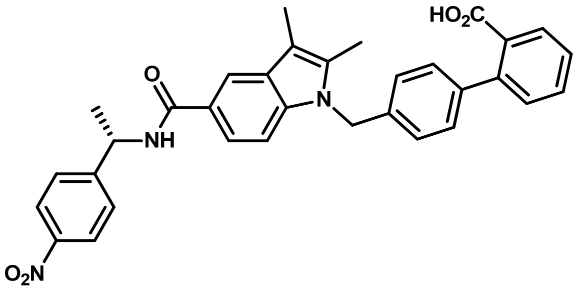

SR1664

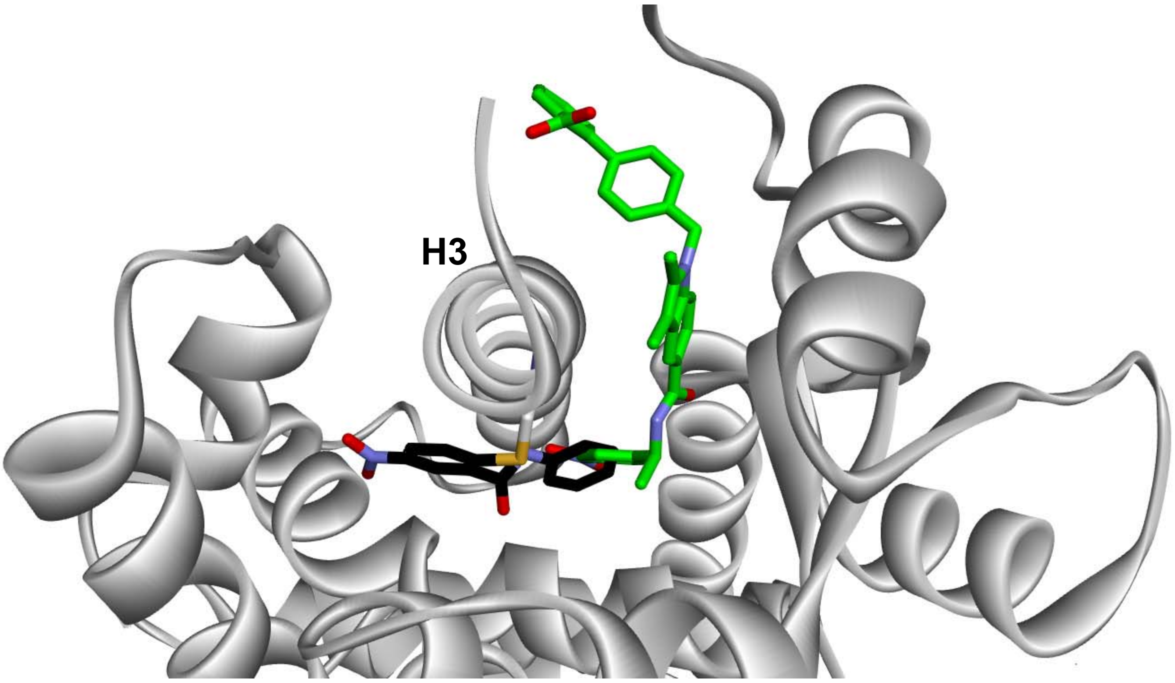

Fig. S2. Alignment of the SR1664–PPARγ LBD and GW9662–PPARγ LBD (PDB: 3B0R) X-ray co-crystal structures. SR1664 (green) exhibits a steric clash with phenyl group of GW9662 (black) which is covalently bound to Cys313 on H3 of PPARγ.

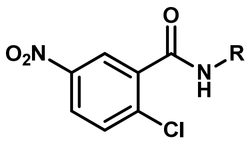

R = phenyl ▶ GW9662

R = methyl ▶ SB1404

2-chloro-5-nitrobenzamide

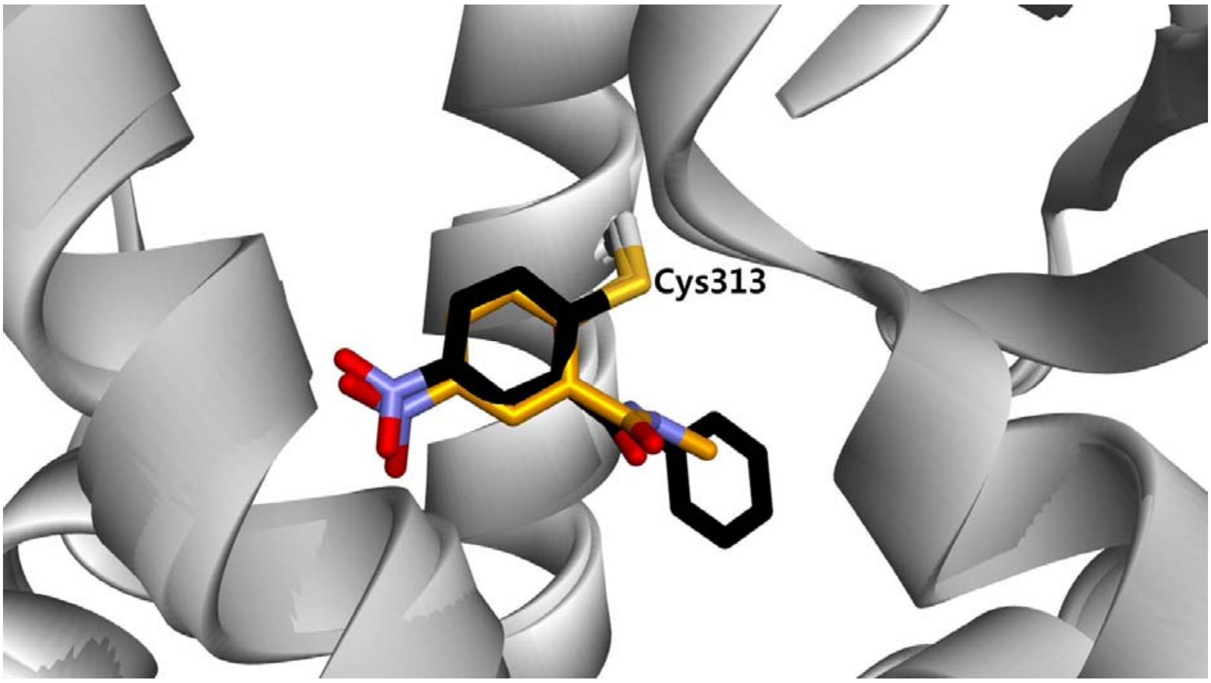

Fig. S3. Alignment of the SB1404–PPARγ LBD and GW9662–PPARγ LBD (PDB: 3B0R) X-ray co-crystal structures. Like GW9662 (black), SB1404 (orange) makes covalent bonding with Cys313 on H3 of PPARγ, indicating that 2-chloro-5-nitrobenzamide moiety can serve as an electrophile and covalently trap Cys313 regardless of functional groups attached to amide.

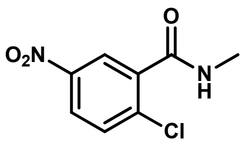

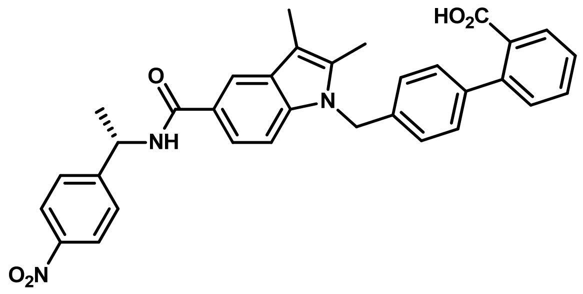

SB1404 **SR1664**

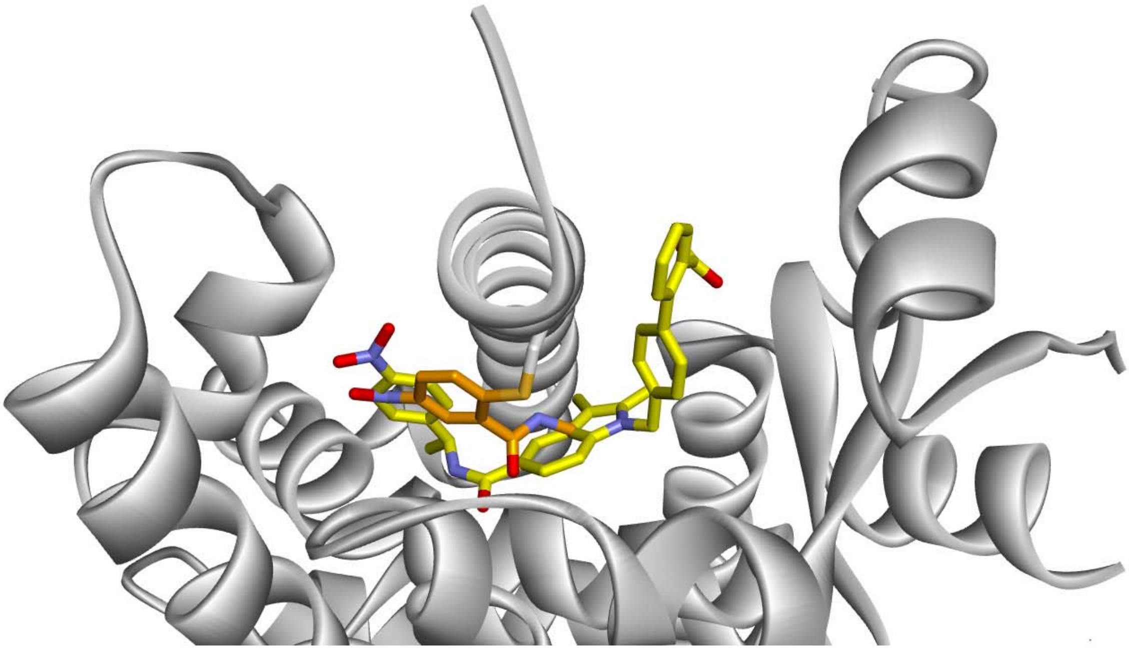

Fig. S4. Alignment of the previously reported SR1664–PPARγ LBD (PDB: 4R2U) and SB1404–PPARγ LBD X-ray co-crystal structures. SR1664 (yellow) overlaps with SB1404 (orange) complexed with PPARγ LBD, indicating SR1664 cannot coexist with SB1404 in the canonical binding pocket of PPARγ through the previously reported binding mode [*Nat. Comm.* **2015** (Ref. 15)] .

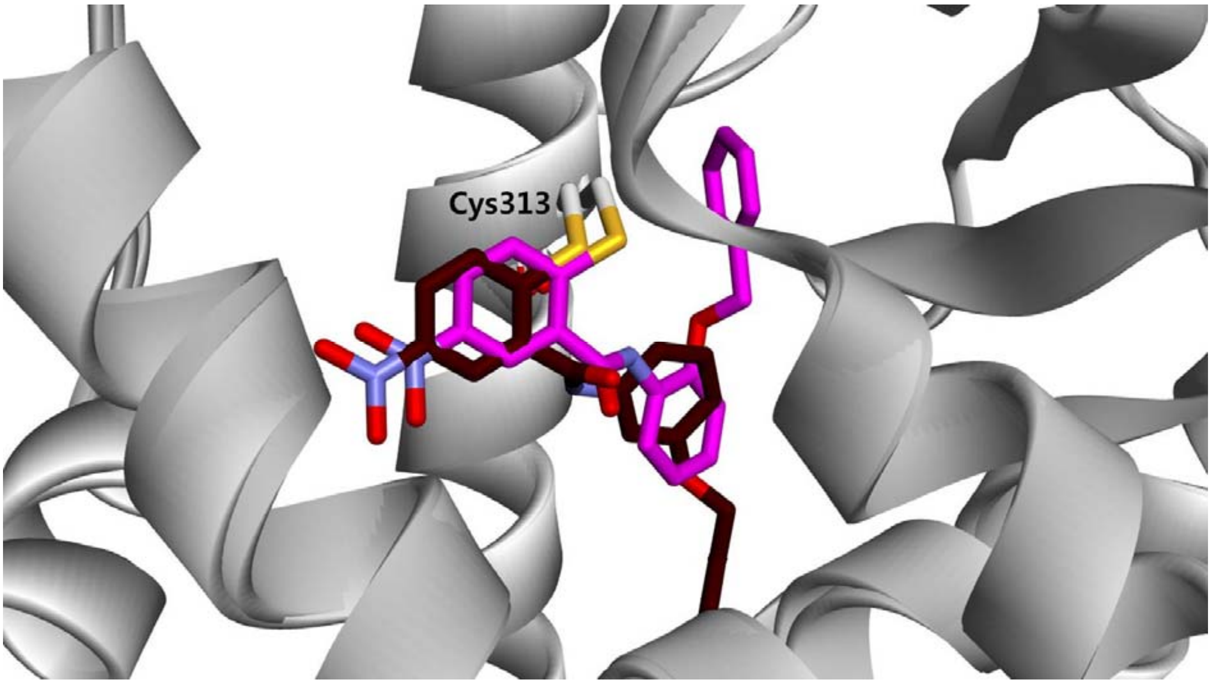

Fig. S5. Alignment of the SB1405–PPARγ LBD and SB1406–PPARγ LBD X-ray co-crystal structures. Both SB1405 (pink) and SB1406 (brown) covalently bind to Cys313 on H3 of PPARγ, but they have different binding modes.

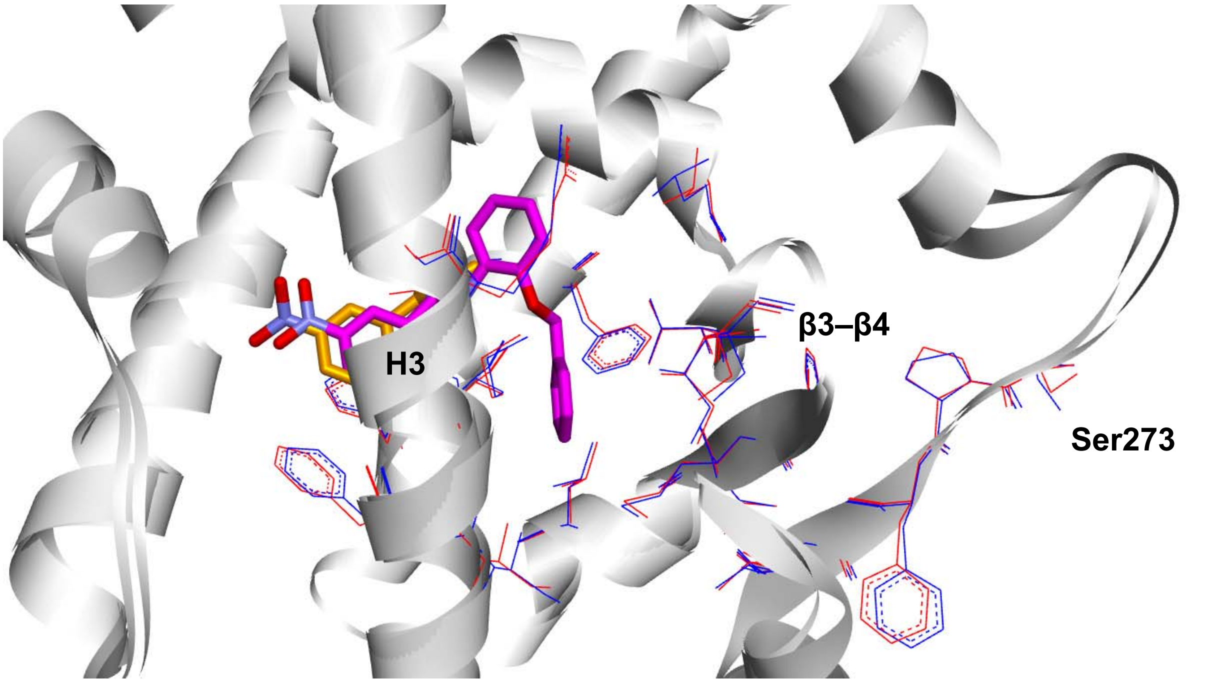

Fig. S6. Alignment of the SB1404–PPARγ LBD and SB1405–PPARγ LBD X-ray co-crystal structures. There is no significant difference between residues' positioning of SB1404–PPARγ LBD co-crystal structure (red) and that of SB1405–PPARγ LBD co-crystal structure (blue) around the specific binding site.

Sequence	Start	End	SB1404						SB1405						MHP	CS
			10s	60s	300s	1800s	7200s	mean	10s	60s	300s	1800s	7200s	mean		
AEISSDIDQLNPESADL	223	239	0.0	0.0	0.0	0.0	0.0	0.0	0.0	0.0	0.0	0.0	0.0	0.0	1815.8152	2
ISSDIDQLNPESADL	225	239	0.0	0.0	0.0	0.0	0.0	0.0	0.0	0.0	0.0	0.0	0.0	0.0	1615.7554	2
ISSDIDQLNPESADLRA	225	241	0.0	0.0	0.0	0.0	0.0	0.0	0.0	0.0	0.0	0.0	0.0	0.0	1842.8878	2
PESADL	234	239	0.0	0.0	0.0	0.0	0.0	0.0	0.0	0.0	0.0	0.0	0.0	0.0	630.2893	1
YIKSFPLTKAKARAIL	250	265	0.0	0.0	0.0	0.0	0.0	0.0	0.0	0.0	0.0	0.0	0.0	0.0	1819.1047	3
TGKTTDKSPFVIYDM	266	280	0.0	0.0	0.0	0.0	0.0	0.0	0.0	0.0	0.0	0.0	0.0	0.0	1701.8200	2
TGKTTDKSPFVIYDMNSLM	266	284	5.6	0.0	2.8	0.0	0.0	1.7	-2.8	0.6	2.8	0.0	0.0	0.1	2147.0144	2
MMGEDKIKFKHITPLQEQSKEVA	284	306	0.0	0.0	0.0	0.0	0.0	0.0	0.0	0.0	0.0	0.0	0.0	0.0	2686.4065	4
FRSVEAVQEITE	315	326	0.0	0.0	-12.5	-10.0	-12.5	-7.0	-25.0	-33.3	-37.5	-33.3	-25.0	-30.8	1406.7056	2
AVQEITEYAKSIPGFVNL	320	337	0.0	0.0	0.0	0.0	0.0	0.0	-5.9	-2.9	-8.8	-17.6	-17.6	-10.6	1978.0386	2
ITEYAKSIPGFVNL	324	337	0.0	-3.8	0.0	0.0	0.0	-0.8	0.0	-3.8	0.0	-3.8	-3.8	-2.3	1550.8332	2
YAKSIPGFVNL	327	337	0.0	5.0	5.0	0.0	0.0	2.0	-5.0	5.0	0.0	0.0	0.0	0.0	1207.6366	2
YAKSIPGFVNLDLND	327	341	3.6	0.0	0.0	0.0	0.0	0.7	0.0	-3.6	0.0	0.0	0.0	-0.7	1664.7870	2
YAKSIPGFVNLDLNDQVTL	327	345	2.8	0.0	2.8	2.8	2.8	2.2	0.0	0.0	0.0	0.0	0.0	0.0	2106.0926	2
PGFVNL	332	337	0.0	0.0	0.0	0.0	0.0	0.0	0.0	0.0	0.0	0.0	0.0	0.0	645.3519	1
DLNDQVTL	338	345	0.0	0.0	0.0	0.0	0.0	0.0	0.0	0.0	0.0	0.0	0.0	0.0	916.4452	1
QVTLLKYGVHEIIYTML	342	358	0.0	0.0	0.0	0.0	0.0	0.0	0.0	0.0	0.0	-2.9	0.0	-0.6	2020.1008	3
LKYGVHEIIY	346	355	0.0	0.0	0.0	0.0	0.0	0.0	0.0	0.0	0.0	-5.0	0.0	-1.0	1233.6306	2
LKYGVHEIIYTML	346	358	-3.8	0.0	0.0	0.0	0.0	-0.8	0.0	0.0	0.0	-3.8	0.0	-0.8	1578.8462	2
TMLASLMNKDGVL	356	368	0.0	-3.8	0.0	0.0	0.0	-0.8	0.0	-7.7	-23.1	-30.8	-30.8	-18.5	1391.7160	2
ISEGQGFMTREFL	369	381	3.8	0.0	3.8	0.0	0.0	1.5	-3.8	-3.8	-19.2	-15.4	-19.2	-12.3	1513.7048	2
ISEGQGFMTREFLKSLRKPFGD	369	390	0.0	0.0	0.0	0.0	0.0	0.0	-4.8	-9.5	-19.0	-19.0	-19.0	-14.3	2542.3233	4
FLKSLRKPFGDF	380	391	0.0	0.0	0.0	0.0	0.0	0.0	-9.1	-4.5	-4.5	-4.5	0.0	-4.5	1453.8061	3
KSLRKPFGDFMEPKFEF	382	398	10.0	5.0	10.0	5.0	0.0	6.0	-15.0	-25.0	-10.0	-5.0	-5.0	-12.0	2102.0716	3
PKFEF	394	398	12.5	12.5	12.5	12.5	12.5	12.5	-12.5	-25.0	-25.0	-12.5	-25.0	-20.0	666.3427	1
AVKFNALELDDSD	399	411	0.0	0.0	0.0	0.0	0.0	0.0	0.0	-3.8	0.0	0.0	0.0	-0.8	1435.6826	2
AVKFNALELDDSD L	399	412	0.0	-3.6	3.6	0.0	0.0	0.0	0.0	-3.6	0.0	-3.6	0.0	-1.4	1548.7760	2
NALELDSDL	403	412	0.0	0.0	0.0	-5.0	0.0	-1.0	-5.0	0.0	-10.0	-5.0	-5.0	-5.0	1103.5060	1
LELDDSDL	405	412	-6.3	-6.3	-6.3	0.0	0.0	-3.8	-6.3	-6.3	-12.5	0.0	0.0	-5.0	918.4162	1
IAVIILSGDRPGL	416	428	0.0	0.0	0.0	0.0	0.0	0.0	0.0	0.0	0.0	0.0	0.0	0.0	1322.7974	2
IAVIILSGDRPGLL	416	429	0.0	0.0	0.0	0.0	0.0	0.0	0.0	-3.8	0.0	0.0	0.0	-0.8	1435.8740	2
VIILSGDRPGLL	418	429	0.0	0.0	0.0	0.0	0.0	0.0	0.0	-4.5	0.0	0.0	0.0	-0.9	1251.7626	2
LSGDRPGLL	421	429	6.3	6.3	6.3	6.3	6.3	6.3	-6.3	-18.8	-18.8	-12.5	-12.5	-13.8	926.4278	2
NVKPIEDIQDNLL	430	442	0.0	0.0	0.0	0.0	0.0	0.0	0.0	0.0	0.0	0.0	0.0	0.0	1509.8016	2
NVKPIEDIQDNLLQA	430	444	0.0	0.0	0.0	0.0	0.0	0.0	0.0	0.0	0.0	0.0	0.0	0.0	1708.8950	2
IQDNLLQALELQLKLNHPESSQL	437	459	0.0	0.0	0.0	0.0	0.0	0.0	0.0	0.0	0.0	0.0	0.0	0.0	2643.4522	3
LELQLKLNHPESSQL	445	459	3.6	0.0	0.0	0.0	0.0	0.7	3.6	0.0	0.0	0.0	0.0	0.7	1747.9548	2
FAKLLQKMTD	460	469	0.0	0.0	5.0	0.0	0.0	1.0	0.0	-5.0	0.0	-5.0	-5.0	-3.0	1193.6288	2
FAKLLQKMTDLRQ	460	472	3.8	0.0	0.0	-3.8	-3.8	-0.8	0.0	0.0	-3.8	-7.7	-7.7	-3.8	1590.8647	3
LQKMTDLRQIVTEHVQL	464	480	0.0	-2.9	0.0	-2.9	0.0	-1.2	0.0	0.0	0.0	0.0	0.0	0.0	2051.1202	3
HVQLLQVIKKTETDMSLHPLL	477	497	0.0	0.0	0.0	0.0	0.0	0.0	0.0	0.0	0.0	0.0	0.0	0.0	2442.3856	3
LQVIKKTETDMSLHPLL	481	497	0.0	0.0	0.0	0.0	0.0	0.0	0.0	0.0	0.0	0.0	0.0	0.0	1965.1064	2
LQVIKKTETDMSLHPLLQE	481	499	0.0	0.0	0.0	-2.8	0.0	-0.6	-5.6	0.0	0.0	-2.8	-2.8	-2.2	2222.2165	3
SLHPLLQEIYKDLY	492	505	0.0	0.0	0.0	0.0	0.0	0.0	0.0	0.0	-3.8	-3.8	-3.8	-2.3	1730.9348	2
HPLLQEIYKDLY	494	505	0.0	0.0	4.5	4.5	0.0	1.8	0.0	0.0	0.0	0.0	0.0	0.0	1530.8072	2

Fig. S7. Differential HDX-MS data for SB1404 and SB1405. The sequence of the peptide is given along with PPARγ2 start / end residue numbers. The %D values are the difference between the mean HDX value obtained from apo PPARγ LBD measured at 5 time points (10 s, 60 s, 300 s, 1800 s, 7200 s). Each experiment was triplicated.

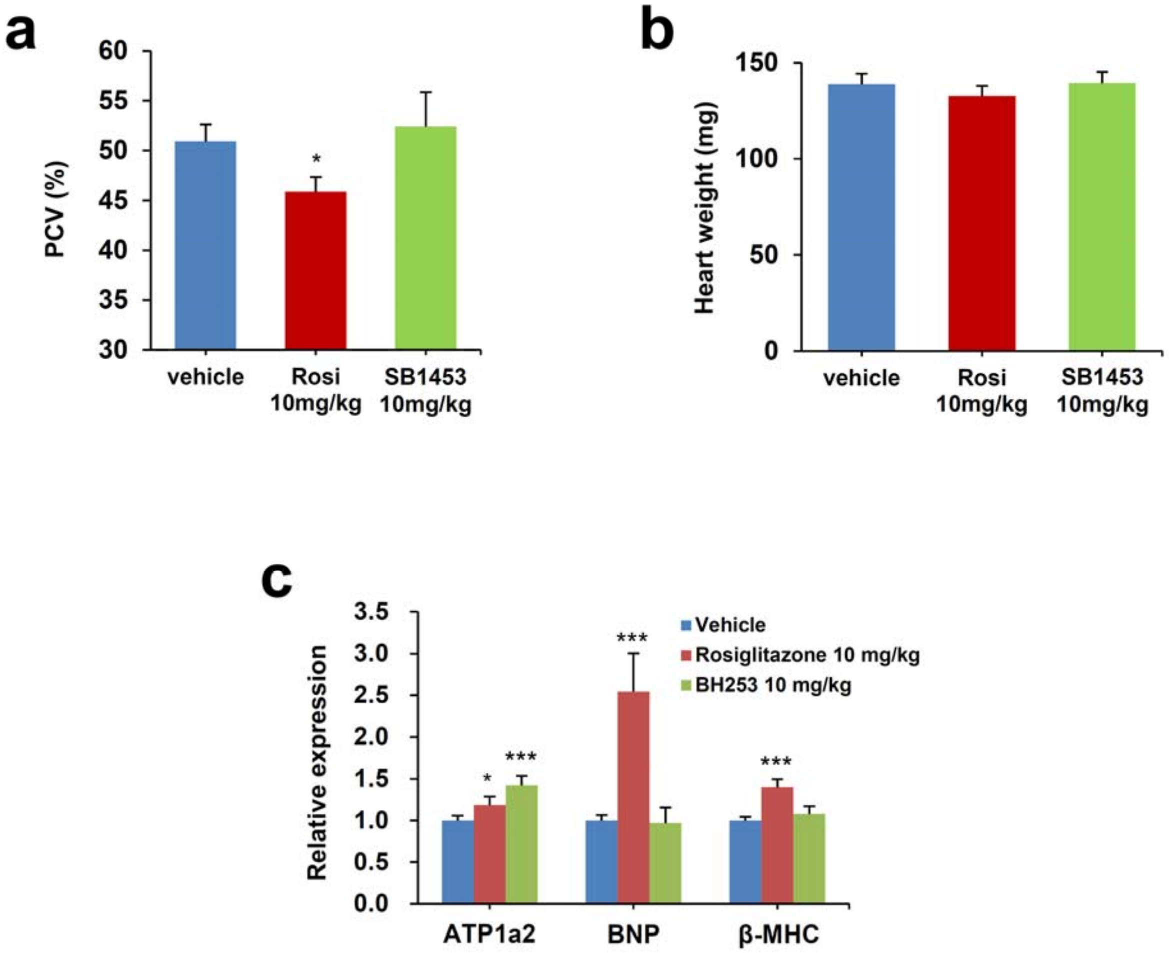

Fig. S8. Adverse effects upon treatment with rosiglitazone or SB1453 *in vivo*. Packed cell volume (PCV) in whole blood (**a**), heart weight (**b**), and the expression of marker genes for heart failure and cardiac hypertrophy in heart (**c**) were determined in high-fat diet-induced obese mice treated with rosiglitazone or SB1453 (14 days, 10 mg/kg/day) (*n=6*). All of represented error bars are S.E.M. (*n=6*). *$p<0.05$, ***$p<0.001$ compared with vehicle.

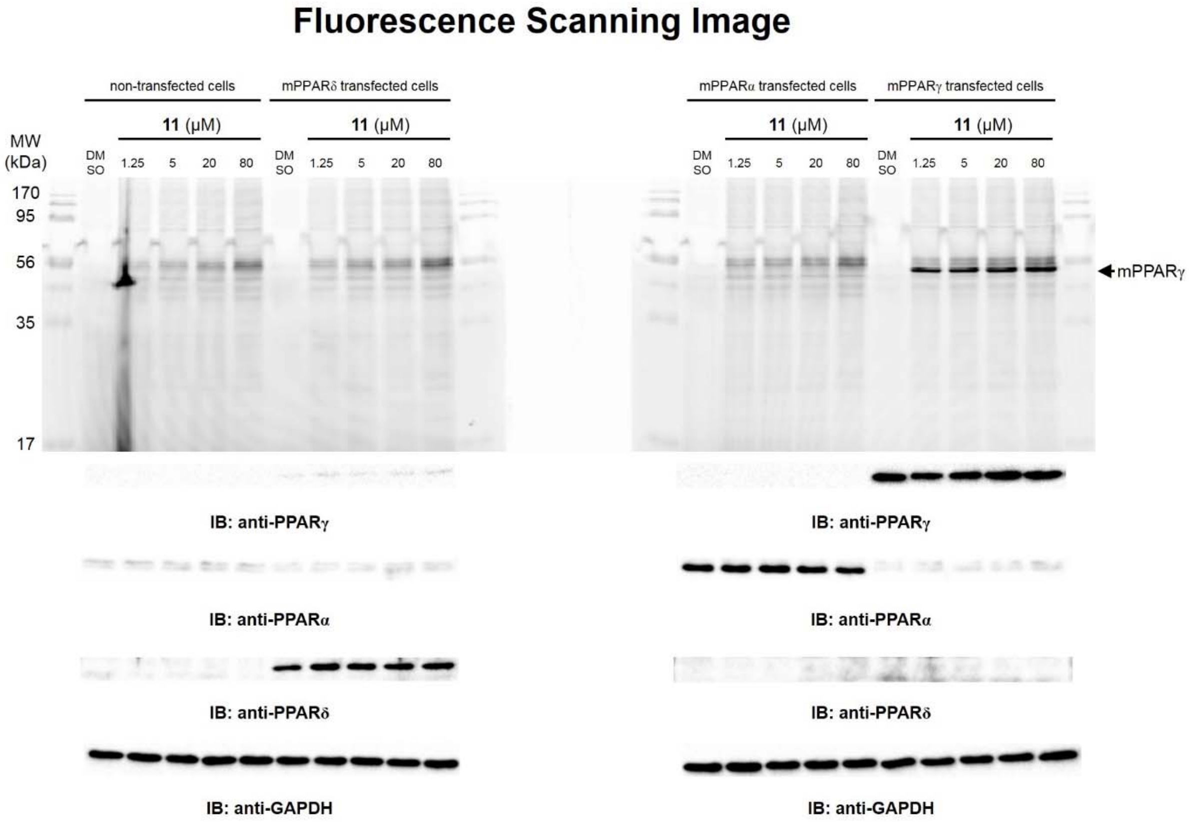

Fig. S9. PPAR subtype selectivity of covalent inhibitor. Predominant fluorescence-labelled protein was observed in lysate of murine PPARγ (mPPARγ) transfected HEK-293T cells when these cells were treated with target identification probe **11**, followed by copper-catalyzed Click reaction with an azide-containing Cy5. In case of non-transfected cells, murine PPARα (mPPARα) transfected cells, and PPARδ (mPPARδ) transfected cells, this kind of fluorescence-labelled protein was not detected.

2. Supplementary Tables

Table S1. Statistics on data collection and refinement

	SB1404	SB1405	SB1406	SB1451	SB1453	SR1664
A. Data collection						
Beamline source[a]	PLS BL-7A	PLS BL-7A	PLS BL-7A	PLS BL-7A	PLS BL-7A	PLS BL-7A
Space group	$P2_12_12$	$P2_12_12$	$P2_12_12$	$P2_12_12$	$P2_12_12$	$P2_12_12$
Unit cell parameters						
a (Å)	53.38	53.95	53.33	55.27	54.86	53.08
b (Å)	130.85	130.89	131.33	131.30	130.28	130.87
c (Å)	53.27	52.59	52.85	51.97	52.25	53.06
$\alpha = \beta = \gamma$ (°)	90	90	90	90	90	90
X-ray wavelength (Å)	0.97934	0.97935	0.97934	0.97933	0.97935	0.97934
Resolution range (Å)	50.0–2.80 (2.85–2.80)[b]	50.0–2.75 (2.80–2.75)[b]	50.0–2.95 (3.00–2.95)[b]	50.0–2.75 (2.80–2.75)[b]	50.0–2.30 (2.34–2.30)[b]	50.0–2.20 (2.24–2.20)[b]
Total / unique reflections	53,885 / 9,648	51,736 / 10,385	56,026 / 8,258	51,628 / 10,377	101,485 / 17,551	135,258 / 19,374
Completeness (%)	98.5 (99.0)[b]	99.5 (100.0)[b]	99.3 (100.0)[b]	99.7 (100.0)[b]	99.7 (100.0)[b]	99.2 (100.0)[b]
$\langle I \rangle / \langle \sigma_I \rangle$	34.0 (4.0)[b]	30.3 (5.0)[b]	38.2 (4.6)[b]	25.2 (4.0)[b]	36.9 (5.2)[b]	42.3 (4.3)[b]
R_{merge}[c] (%)	6.1 (52.8)[b]	7.6 (41.6)[b]	6.4 (57.8)[b]	8.9 (64.9)[b]	7.2 (47.2)[b]	7.2 (55.9)[b]
B. Model refinement						
Resolution range (Å)	30.0–2.80	30.0–2.75	30.0–2.95	30.0–2.75	30.0–2.30	30.0–2.20
R_{work} / R_{free}[d] (%)	20.8 / 24.4	20.2 / 23.0	20.5 / 25.7	19.2 / 24.3	19.6 / 24.1	20.6 / 23.8
No. of non-hydrogen atoms						
Protein	2189	2201	2154	2247	2252	2264
Ligand	13	26	26	44	44	41
Water oxygen	32	25	25	39	87	73
Wilson *B* factor (Å^2)	75.0	66.6	78.6	55.1	45.7	48.8
Average *B* factor (Å^2)						
Protein	83.3	78.5	86.2	59.7	58.4	65.4
Ligand	98.6	78.3	84.5	59.4	60.0	64.9
Water oxygen	81.0	69.0	65.0	50.1	59.6	62.6
R.m.s. deviations from ideal geometry						
Bond lengths (Å)	0.011	0.009	0.009	0.010	0.010	0.013
Bond angles (°)	1.48	1.45	1.47	1.43	1.50	1.49
Ramachandran plot[e]						
Favored / Outliers (%)	97.0 / 0.0	97.4 / 0.0	97.3 / 0.0	97.1 / 0.0	98.5 / 0.0	98.2 / 0.0
Poor rotamers (%)	0.00	0.00	0.00	0.00	0.00	0.00

[a] PLS stands for Pohang Light Source, Korea.

[b] Values in parentheses refer to the highest resolution shell.

[c] $R_{merge} = \Sigma_h \Sigma_i \mid I(h)_i - \langle I(h) \rangle \mid / \Sigma_h \Sigma_i I(h)_i$, where $I(h)$ is the intensity of reflection h, Σ_h is the sum over all reflections, and Σ_i is the sum over i measurements of reflection h.

[d] $R_{work} = \Sigma \mid |F_{obs}| - |F_{calc}| \mid / \Sigma |F_{obs}|$, where R_{free} is calculated for a randomly chosen 5% of reflections, which were not used for structure refinement and R_{work} is calculated for the remaining reflections.

[e] Values obtained using *MolProbity*.

Table S2. Primer sequences used for qPCR		
**Gene**	**Forward primer**	**Reverse primer**
aP2	AAGGTGAAGAGCATCATAACCCT	TCACGCCTTTCATAACACATTCC
Adiponectin	TGTTCCTCTTAATCCTGCCCA	CCAACCTGCACAAGTTCCCTT
Adipsin	CATGCTCGGCCCTACATGG	CACAGAGTCGTCATCCGTCAC
Cyp2f2	GTCGGTGTTCACGGTGTACC	AAAGTTCCGCAGGATTTGGAC
Rarres2	GCCTGGCCTGCATTAAAATGG	CTTGCTTCAGAATTGGGCAGT
Selenbp1	ATGGCTACAAAATGCACAAAGTG	CCTGTGTTCCGGTAAATGCAG
Car3	TGACAGGTCTATGCTGAGGGG	CAGCGTATTTTACTCCGTCCAC
Peg10	TGCTTGCACAGAGCTACAGTC	AGTTTGGGATAGGGGCTGCT
Cidec	ATGGACTACGCCATGAAGTCT	CGGTGCTAACACGACAGGG
Cd24a	GTTGCACCGTTTCCCGGTAA	CCCCTCTGGTGGTAGCGTTA
Acyl	CAGCCAAGGCAATTTCAGAGC	CTCGACGTTTGATTAACTGGTCT
Nr1d2	TGAACGCAGGAGGTGTGATTG	GAGGACTGGAAGCTATTCTCAGA
Ddx17	TCTTCAGCCAACAATCCCAATC	GGCTCTATCGGTTTCACTACG
Aplp2	GTGGTGGAAGACCGTGACTAC	TCGGGGGAACTTTAACATCGT
Nr3c1	AGCTCCCCCTGGTAGAGAC	GGTGAAGACGCAGAAACCTTG
Rybp	CGACCAGGCCAAAAAGACAAG	CACATCGCAGATGCTGCATT
Txnip	TCTTTTGAGGTGGTCTTCAACG	GCTTTGACTCGGGTAACTTCACA
Nr1d1	TACATTGGCTCTAGTGGCTCC	CAGTAGGTGATGGTGGGAAGTA
Cycs	CCAAATCTCCACGGTCTGTTC	ATCAGGGTATCCTCTCCCCAG
Ppcs	CGCTTTCTGGACAACTTCAGT	GGGAGCGCATTCTCTTCGG
Fdx1	CAAGGGGAAAATTGGCGACTC	TTGGTCAGACAAACTTGGCAG
Fgfrl1	ATGGCCGCACAATCCACAG	TGGTGGCCTTGCACACATAAA
Idh3a	TGGGTGTCCAAGGTCTCTC	CTCCCACTGAATAGGTGCTTTG
Abhd12	GTCACCTTGGAGCATGAGC	GCAATGTAGAACCCCAGAACAC
Nadk	TCATGGGGATGAGACCTGGAG	ACAAGCACACTCTTGGGAGAC
Arhgap5	TTGGACTCTCTGGGACTGAAA	AGCACAGAAGTATGCTCTGGA
Pdk4	AGGGAGGTCGAGCTGTTCTC	GGAGTGTTCACTAAGCGGTCA
Las1l	GGAGGTGAACATTCCAGACTG	CTCATCCAACTCCCAGGTTTC
Cib2	GACAACTACCAGGACTGCACT	CCATCCTCGGAGAAAGCCTC
Fmr1	CAATGGCGCTTTCTACAAGGC	TCTGGTTGCCAGTTGTTTTCA
Pim3	AAGGACACGGTCTACACTGAC	GACACCACTCAATAAGCTGCT
Phospho1	CTCACCTTCGACTTCGATGAGA	CCCAGGTACTTAAAGACTCGTTG
Plin2	GACCTTGTGTCCTCCGCTTAT	CAACCGCAATTTGTGGCTC
Lass4	TACCCACATCAGACCCTGAAT	TCATGGGGATGAGACCTGGAG

3. Biochemical Procedures

Reagents. SR1664 (SML0636, Sigma), GW9662 (M6191, Sigma), and rosiglitazone (R0106, TCI) were purchased from commercial venders and used without further purification. Cell culture reagents, including serum, medium, and antibiotic-antimycotic solution, were obtained from Gibco, Invitrogen.

Cell culture. HEK-293, HEK-293T and 3T3-L1 cells were obtained from American Type Culture Collection and cultured in Dulbecco's modified Eagle's medium (DMEM) supplemented with 1% (v/v) antibiotic-antimycotic solution and 10% fetal bovine serum (FBS). 3T3-L1 preadipocytes were grown in 6-well plates to 100% confluency and induced by treating the cells with 1 μM dexamethasone, 850 nM insulin, and 10 μM compound in DMEM containing 10% FBS for 2 days, and the cell culture was subsequently maintained by replacing the medium with fresh DMEM containing 10% FBS and 850 nM insulin. At day 8, accumulated lipid in the 3T3-L1 cells was detected by Oil Red O staining.

***In vitro* kinase assay.** *In vitro* Cdk5 assay was conducted according to the manufacturer's instructions [Cell Signaling Technology, USA]. Briefly, 0.5 μg of purified PPARγ LBD was incubated with active Cdk5/p35 [Millipore, USA] in assay buffer (25 mM Tris-HCl, pH 7.5, 5 mM β-glycerophosphate, 2 mM DTT, 0.1 mM Na_3VO_4, 10 mM $MgCl_2$) containing 25 μM ATP for 30 min at 30 °C. Compounds were pre-incubated with PPARγ LBD for 30 min at 30 °C before performing the assay. Rb-peptide [residues 773–928, Millipore] was also used as a substrate of Cdk5 to know whether the compounds affect the fundamental kinase function of Cdk5 or not. Phosphorylation of substrates was analyzed by western blotting with anti-Cdk substrate antibody to detect phospho-Ser in a K/R-S-P-K/R motif, which is the consensus motif for Cdk substrates

[Cell Signaling Technology, USA].

Cell-based luciferase reporter gene assay. HEK-293T cells were seeded in 96-well plates at a density of 7000 cells per well a day prior to transfection. The cells were transfected with pDR-1 luciferase reporter plasmid, PPARγ, RXRα, and pRL-renillin using the calcium phosphate transfection protocol. Following an overnight transfection, the cells were treated with rosiglitazone, SR1664, SB1451, or SB1453 for 24 h. The cells were harvested and reporter gene assay were performed by using the Dual-luciferase kit [Promega, USA]. Luciferase activity was measured using Bio-Tek microplate reader [ELx800TM, Bio-Tek Instruments Inc., USA] and normalized to Renilla activity. Fold change of treated cells over DMSO-treated control cells were plotted in triplicates.

Preparation of cell or tissue lysates and immunoblotting. HEK-293 cells expressing PPARγ were treated with phorbol 12-myristate 13-acetate (PMA) (0.5 μM) for 30 min and total cell lysates were incubated with FLAG M2 agarose [Sigma Aldrich, USA] at 4 °C. For tissue lysates, WAT from mice was homogenized in RIPA buffer (50 mM Tris, pH 7.5, 150 mM NaCl, 1% NP-40, 0.5% sodium deoxycholate, 0.1% SDS with protease and phosphatase inhibitors). Immunoprecipitates, total cell lysates or tissue lysates were analyzed with phospho-specific antibody against PPARγ Ser273 or anti-PPARγ antibody [Santa Cruz Biotechnology, Inc., USA].

Gene expression analysis. Total RNA was isolated from cells or tissues using Trizol reagents [Invitrogen, CA, USA]. The RNA was reverse-transcribed using ABI reverse transcription kit. Quantitative PCR reactions were performed with SYBR green fluorescent dye using an ABI9300 PCR machine. Relative mRNA expression was determined by the ΔΔ-Ct method normalized to TATA-binding protein (TBP) levels.

Hydrogen-deuterium exchange mass spectrometry (HDX-MS) PPARγ LBD (aa 223~505) 25 μM were incubated with 50 μM SB1404 or SB1405 in 5% ACN for 30 min at R.T., followed by incubating with 20-fold D_2O at 25 °C for the various times: 10, 60, 300, 1800, and 7200 s. The deuterium labeling reaction was quenched by 2.5 mM tris (2-carboxyethyl) phosphine (TCEP) in formic acid, pH 2.3. For protein digestion, 1 μg of porcine pepsin was added to each quenched protein sample and incubated on ice for 3 min before injection. Peptic peptides were separated and identified by nanoAcquity^{TM}/ESI/MS (SYNAPT^{TM} HDMS^{TM}) [Waters, UK]. Briefly, peptides were desalted on C18 trap column cartridge [Waters, UK] and separated on analytical column (BEH130 C18, 1.7 μm particle size, 100 μm i.d. x 100 mm) by gradient elution from 8% ACN to 40% ACN, 0.1% formic acid [Waters, UK] at a flow rate 0.5 μL/min for 7 min. The auto-sampler chamber was set at 5°C. The trap, analytical column and all tubing were immersed in an ice bath, 0°C, to minimize deuterium back-exchange. Separated peptic peptides in gradient chromatography were identified by spraying on line to ESI/MS/MS. We obtained the MS coverage of identified peptides was 94%. The extent of deuterium exchange was monitored the increase in mass of the isotope distribution for each identified peptide, and calculated by Microsoft Excel. The theoretical maximum deuterium exchange value was calculated for each peptide based on the number of exchangeable amides. Each experiment was triplicated.

Target identification using fluorescence labeling. HEK-293T cells expressing murine PPARα, PPARδ, or PPARγ, or differentiated 3T3-L1 cells were scrapped with cold phosphate buffered saline (PBS) and centrifuged. The supernatant was discarded and cell pellet was resuspended in PBS containing protease inhibitor cocktail. The cells were lysed by freeze-thaw cycles, and the cell lysate was centrifuged at 4 °C,

13000 rpm for 15 min. The mixture of proteome and **11** was incubated at 30 °C for 1 h. Click chemistry was performed to the mixture with Cy5-azide [Lumiprobe, USA] (80 μM), TBTA (100 μM), CuSO4 (1 mM), TCEP (2 mM) and *t*BuOH (5%) for 1.5 h. The resulting proteome was separated by gel electrophoresis and scanned with Typhoon Trio [GE Healthcare, UK]. The in-gel fluorescence signal was visualized at the Cy5 (633 nm excitation) channel by Typhoon Trio and analyzed by ImageQuant TL program [Amersham Bioscience, USA]. The fluorescence labeled protein was identified by western blotting with anti-PPARγ antibody [Cell Signaling Technology, USA].

4. Crystallography

Protein expression and purification. The human PPARγ LBD construct (residues 195–477 in PPARγ1) was PCR-amplified and cloned into the expression vector pET-28b(+) [Novagen, USA]. This construct of the recombinant protein encodes a 21-residue *N*-terminal tail (MGSSHHHHHH SSGLVPRGSH M) containing a His_6 tag and a thrombin cleavage site in front of the starting residue Ala195. The recombinant human PPARγ LBD was overexpressed in *Escherichia coli* Rosetta 2(DE3) cells using the Luria Broth culture medium. Protein expression was induced by 0.5 mM isopropyl β-D-thiogalactopyranoside and the cells were incubated for additional 24 h at 18 °C following growth to mid-log phase at 37 °C. The cells were lysed by sonication in buffer A (20 mM Tris-HCl at pH 8.5, 150 mM NaCl, 10% (v/v) glycerol and 0.1 mM tris(2-carboxyethyl) phosphine hydrochloride) containing 5 mM imidazole and 1 mM phenylmethylsulfonyl fluoride. The crude lysate was centrifuged at 36,000 g for 1 h. The supernatant was applied to a HiTrap Chelating HP affinity chromatography column [GE Healthcare, UK], which was previously equilibrated with buffer A containing 5 mM imidazole. Upon eluting with a gradient of imidazole in the same buffer, the human PPARγ LBD was eluted at 45–100 mM imidazole concentration. The eluted protein was desalted in buffer A by HiPrep 26/10 desalting column [GE Healthcare, UK] to remove imidazole, and the protein was cleaved with 2 U/mg thrombin protease [Sigma Aldrich, USA] at 4 °C overnight. The *N*-terminal fusion tag and uncleaved material were removed by rechromatography with a HiTrap Chelating HP affinity chromatography column. The flow-through was applied to a HiLoad XK-16 Superdex 200 prep-grade column [GE Healthcare, UK], which was previously equilibrated with buffer A. Fractions containing the human PPARγ LBD were pooled and concentrated

to 15.4 mg/mL using an Amicon Ultra-15 Centrifugal Filter Unit [Millipore, USA].

Crystallization. Before crystallization, the purified PPARγ LBD and a LXXLL motif-containing peptide derived from human steroid receptor coactivator-1 (SRC1) (residues 685–700, ERHKILHRLLQEGSPS) were mixed in a ratio of 1:2, with a 10-fold molar excess of the PPARγ ligands. After overnight incubation, the protein was crystallized by the sitting-drop vapor diffusion method using the Mosquito robotic system [TTP Labtech, UK] at 23 °C by mixing 0.2 μL of the protein solution and 0.2 μL of the reservoir solution. Crystals of PPARγ LBD in complex with various ligands and the SRC1 peptide were obtained with a reservoir solution of 2.2 M sodium malonate (pH 7.0), except for crystals of PPARγ LBD in complex with SB1451. The PPARγ·SB1451·SRC1 crystals were obtained with a reservoir solution of 100 mM sodium cacodylate (pH 6.5), 190 mM sodium acetate and 26% (w/v) PEG 8000. All structures were deposited in the protein data bank: SR1664 (PDB: 5DWL), SB1404 (PDB: 5DV6), SB1405 (PDB: 5DV3), SB1406 (PDB: 5DSH), SB1451 (PDB: 5DV8) and SB1453 (PDB: 5DVC).

X-ray data collection. X-ray diffraction data were collected at 100 K using a Quantum 270 CCD detector system [Area Detector Systems Corporation, USA] at the BL-7A experimental station of Pohang Light Source, Korea. Raw X-ray diffraction data were processed and scaled using the program suit HKL2000[1]. Data collection statistics are summarized in **Table S1**. All structures were solved by molecular replacement with the program *MolRep*[2] using the previously published PPARγ LBD structure (PDB: 3VN2)[3] as a search model. Subsequent model building was done manually using the program *COOT*[4] and the models were refined with the program *REFMAC5*[5], including the bulk solvent correction. A total of 5% of the data was randomly set aside as test

data for the calculation of R_{free}.[6] The stereochemistry of the refined models was assessed by *MolProbity*[7]. Refinement statistics are summarized in **Table S1**.

5. *In vivo* studies

Animals. All animal experiments were performed according to procedures approved by Ulsan National Institute of Science and Technology's Institutional Animal Care and Use Committee. 5-week-old male C57BL/6J mice [DBL, Korea] were fed a high fat diet [60% kcal fat, D12492, Research Diets Inc., NJ, USA] for 10 weeks. For glucose tolerant tests (GTTs), mice were intraperitoneally injected daily 10 mg/kg rosiglitazone, SB1453, or vehicle for 7 days, and fasted overnight prior to injection of 1.5 g/kg D-glucose. Glucose was measured in tail vein blood at intervals after glucose injection using a Truetrack glucometer [Nipro Diagnostics, Japan]. For analysis of adverse effects, mice were intraperitoneally injected daily 10 mg/kg rosiglitazone, SB1453, or vehicle for 14 days. The packed cell volume (PCV) was determined by dividing the volume of packed red blood cells by the total volume of blood followed by centrifuging whole blood in a capillary tube.

Statistical analysis. Data are presented as means ± standard errors of the means (SEMs) as indicated in the figure legends. Comparisons between two groups were made by unpaired two-tailed Student's *t*-tests. P values of <0.05 were considered statistically significant. Microsoft Excel was used for statistical calculations.

6. Chemical Synthesis

The section below describes the synthesis of compounds which are used in this study.

General information

All chemicals and solvents were purchased from commercial venders and used without further purification unless noted otherwise. Analytical thin layer chromatography (TLC) was performed on pre-coated glass-backed plates (silica gel 60; F_{254} 0.25 mm) with visualization by ultraviolet (UV) irradiation at 254 nm and/or staining with ninhydrin solution. The products were purified by flash column chromatography on silica gel (230–400 mesh). ^{1}H and ^{13}C NMR spectra were recorded on Bruker DRX-300 [Bruker Biospin, Germany], Agilent 400-MR DD2 [Agilent, USA] or Varian Inova-500 [Varian Assoc., Palo Alto, USA]. ^{1}H chemical shifts are reported in ppm from tetramethylsilane (TMS) as internal standard. Multiplicity was indicated as follows: s (singlet), d (doublet), t (triplet), q (quartet), p (pentet), m (multiplet), dd (doublet of doublet), dt (doublet of triplet), td (triplet of doublet), brs (broad singlet), etc. Coupling constants are reported in Hertz (Hz). ^{13}C chemical shifts are reported in ppm relative to chloroform-*d* (δ 77.16, triplet) or DMSO-d_6 (δ 39.52, septet). Mass spectrometric analysis was performed with Finnigan Surveyor MSQ Plus LC/MS [Thermo Electron Corp., USA] or LCMS-2020 [Shimadzu Corp., JAPAN] using electrospray ionization (ESI).

General procedure for synthesis of compounds 1–4 and 6

To a solution of 2-chloro-5-nitrobenzoyl chloride (500 mg, 2.3 mmol) and amine (1.5 equiv.) in dichloromethane (20 mL), triethylamine (2.5 equiv.) was added and the mixture was stirred at room temperature for 1 h. After removal of solvent and triethylamine under the reduced pressure, the residue was dissolved with 1N HCl and dichloromethane. The organic layer was separated and the aqueous layer was extracted two times with dichloromethane. The combined organic layer was dried over anhydrous Na_2SO_4(s) and filtered through a celite-packed glass filter. The filtrate was concentrated under the reduced pressure and purified by silica-gel flash column chromatography (1:5~1:2 = ethyl acetate:*n*-hexane, v/v) to obtain the desired product.

Compound 1 (SB1404), 2-chloro-*N*-methyl-5-nitrobenzamide

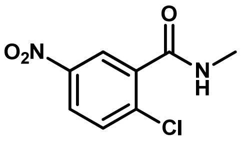

Yield: 68% as white solid; ^{1}H NMR (400 MHz, $CDCl_3$) δ 8.51 (d, *J* = 2.8 Hz, 1H), 8.21 (dd, *J* = 8.4, 2.8 Hz, 1H), 7.60 (d, *J* = 8.8 Hz, 1H), 6.23 (brs, 1H), 3.07 (d, *J* = 4.8 Hz, 1H); ^{13}C NMR (100 MHz, $CDCl_3$) δ 165.0, 146.7, 137.7, 136.6, 131.6, 125.8, 125.4, 27.2; LRMS (ESI) *m/z* calcd for $C_8H_8ClN_2O_3$ $[M+H]^+$: 215.02; Found: 215.06.

Compound 2 (SB1405), *N*-(2-(benzyloxy)phenyl)-2-chloro-5-nitrobenzamide

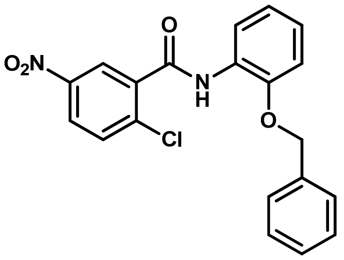

Yield: 98% as light yellow solid; ^{1}H NMR (400 MHz, $CDCl_3$) δ 8.78 (brs, 1H), 8.64 (d, *J* = 2.8 Hz, 1H), 8.48 (dd, *J* = 7.6, 1.2 Hz, 1H), 8.16 (dd, *J* = 8.8, 2.4 Hz, 1H), 7.48 (d, *J* = 8.8 Hz, 1H), 7.41–7.34 (m, 5H), 7.10 (d, *J* = 7.6, 1.6 Hz, 1H),

7.05–7.00 (m, 2H), 5.11 (s, 2H); ^{13}C NMR (100 MHz, $CDCl_3$) δ 161.5, 147.8, 146.7, 137.5, 136.2, 136.0, 131.7, 128.8, 128.6, 128.0, 127.3, 126.1, 125.9, 125.0, 121.6, 120.6, 111.8, 71.2; LRMS (ESI) *m/z* calcd for $C_{20}H_{16}ClN_2O_4$ $[M+H]^+$: 383.08; Found: 382.91.

Compound 3 (SB1406), *N*-(3-(benzyloxy)phenyl)-2-chloro-5-nitrobenzamide

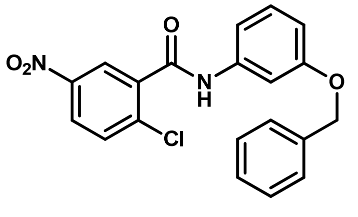

Yield: 98% as white solid; ^1H NMR (400 MHz, $CDCl_3$) δ 8.58 (d, *J* = 2.4 Hz, 1H), 8.23 (dd, J = 8.8, 2.4 Hz, 1H), 7.84 (s, 1H), 7.64 (d, J = 8.8 Hz, 1H), 7.45–7.24 (m, 7H), 7.09 (d, J = 8.4 Hz, 1H), 6.81 (dd, J = 8.4, 2.0 Hz, 1H), 5.07 (s, 2H); ^{13}C NMR (100 MHz, $CDCl_3$) δ 162.4, 159.5, 146.7, 138.2, 137.7, 136.8, 136.6, 131.7, 130.1, 128.7, 128.2, 127.7, 126.1, 125.3, 112.8, 112.2, 107.2 70.2; LRMS (ESI) *m/z* calcd for $C_{20}H_{16}ClN_2O_4$ $[M+H]^+$: 383.08; Found: 382.87.

Compound 4, 2-chloro-5-nitro-*N*-(2-phenoxyphenyl)benzamide

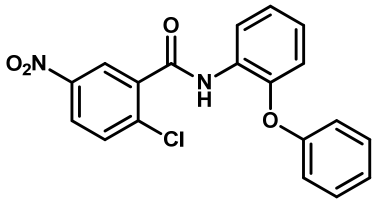

Yield: 94% as off-white solid; ^1H NMR (500 MHz, $CDCl_3$) δ 8.63 (s, 1H), 8.60 (d, *J* = 2.5 Hz, 1H), 8.58 (dd, *J* = 7.5, 1.5 Hz, 1H), 8.23 (dd, *J* = 7.5, 2.5 Hz, 1H), 7.60 (d, *J* = 8.5 Hz, 1H), 7.38–7.35 (m, 2H), 7.21–7.10 (m, 3H), 7.02 (d, *J* = 9.0 Hz, 2H), 6.92 (dd, *J* = 8.0, 1.5 Hz, 1H); ^{13}C NMR (100 MHz, $CDCl_3$) δ 161.9, 156.4, 146.8, 146.2, 137.5, 136.4, 131.7, 130.1, 129.1, 126.0, 125.8, 125.3, 124.3, 124.2, 121.4, 118.6, 118.1; LRMS (ESI) *m/z* calcd for $C_{19}H_{14}ClN_2O_4$ $[M+H]^+$: 369.06; Found: 369.05.

Compound 6, 2-chloro-*N*-(2-ethoxyphenyl)-5-nitrobenzamide

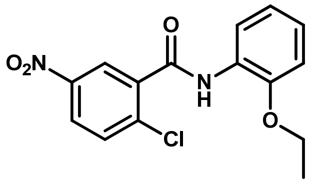

Yield: 95% as off-white solid; ^{1}H NMR (400 MHz, $CDCl_3$) δ 8.77 (brs, 1H), 8.72 (d, *J* = 2.8 Hz, 1H), 8.50 (dd, *J* = 8.0, 1.2 Hz, 1H), 8.25 (dd, *J* = 8.8, 2.8 Hz, 1H), 7.66 (d, *J* = 8.8 Hz, 1H), 7.13 (td, *J* = 8.0, 1.2 Hz, 1H), 7.02 (t, *J* = 8.0 Hz, 1H), 6.92 (d, *J* = 8.0 Hz, 1H), 4.14 (q, *J* = 6.8 Hz, 2H), 1.45 (t, *J* = 6.8 Hz, 3H); ^{13}C NMR (100 MHz, $CDCl_3$) δ 161.5, 147.7, 146.9, 137.5, 136.5, 131.9, 127.1, 126.2, 126.0, 125.0, 121.2, 120.3, 111.1, 64.4, 15.0; LRMS (ESI) *m/z* calcd for $C_{15}H_{14}ClN_2O_4$ $[M+H]^+$: 321.06; Found: 321.15.

General procedure for synthesis of compounds 5 and 7

N-Boc-2-aminophenol (100 mg, 0.48 mmol), alkyl bromide (2.0 equiv.), and K_2CO_3 (1.5 equiv.) were dissolved in anhydrous DMF (15 mL) under argon atmosphere. The reaction mixture was stirred at 80 °C for 12 h, and the crude mixture was diluted with deionized water. The aqueous layer was extracted three times with ethyl acetate. The combined organic layer was dried over anhydrous Na_2SO_4(s) and filtered through a celite-packed glass filter. The filtrate was concentrated under the reduced pressure and purified by silica-gel flash column chromatography (1:10 = ethyl acetate:*n*-hexane, v/v) to obtain *N*-Boc-2-substituted aniline. After Boc-deprotection with 10% trifluoroacetic acid in dichloromethane (10 mL) at room temperature for 1 h, the reaction mixture was washed with saturated aqueous $NaHCO_3$ three times, and concentrated under the reduced pressure to provide 2-substituted aniline. To a solution of 2-substituted aniline in dichloromethane (10 mL), 2-chloro-5-nitrobenzoyl chloride (1.5 equiv.) and triethylamine (2.0 equiv.) were added and the mixture was stirred at

room temperature for 1 h. After removal of solvent and triethylamine under the reduced pressure, the residue was purified by silica-gel flash column chromatography (1:5 = ethyl acetate:*n*-hexane, v/v) to obtain the desired product.

Compound 5, 2-chloro-5-nitro-*N*-(2-phenethoxyphenyl)benzamide

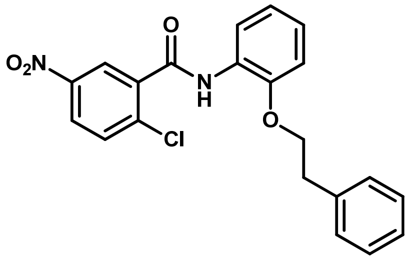

Yield: 63% as light yellow solid; ^{1}H NMR (300 MHz, $CDCl_3$) δ 8.54 (d, *J* = 5.7 Hz, 1H), 8.46 (dd, *J* = 8.1, 1.8 Hz, 1H), 8.36 (s, 1H), 8.26 (dd, *J* = 8.7, 2.7 Hz, 1H), 7.64 (d, *J* = 8.7 Hz, 1H), 7.16–7.08 (m, 6H), 7.04 (td, *J* = 7.8, 1.5 Hz, 1H), 6.95 (dd, *J* = 7.8, 1.5 Hz, 1H), 4.30 (t, *J* = 6.6 Hz, 2H), 3.10 (t, *J* = 6.6 Hz, 2H); ^{13}C NMR (75 MHz, $CDCl_3$) δ 161.7, 147.4, 146.8, 137.8, 137.7, 136.9 131.7, 128.8, 128.6, 127.3, 126.7, 125.9, 125.5, 125.0, 121.6, 120.4, 111.6, 69.1, 35.6; LRMS (ESI) *m/z* calcd for $C_{21}H_{18}ClN_2O_4$ $[M+H]^+$: 397.10; Found: 397.15.

Compound 7, 2-chloro-*N*-(2-(hexyloxy)phenyl)-5-nitrobenzamide

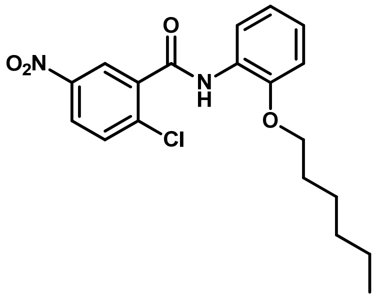

Yield: 50% as white solid; ^{1}H NMR (400 MHz, $CDCl_3$) δ 8.76 (brs, 1H), 8.72 (d, *J* = 2.8 Hz, 1H), 8.50 (dd, *J* = 8.0, 1.2 Hz, 1H), 8.25 (dd, *J* = 8.8, 2.8 Hz, 1H), 7.66 (d, *J* = 8.8 Hz, 1H), 7.13 (t, *J* = 8.0, 1H), 7.02 (t, *J* = 8.0 Hz, 1H), 6.92 (d, *J* = 8.0 Hz, 1H), 4.06 (t, *J* = 6.8 Hz, 2H), 1.81 (m, 2H), 1.45 (m, 2H), 1.32 (m, 4H), 0.87 (t, *J* = 6.8 Hz, 3H); ^{13}C NMR (100 MHz, $CDCl_3$) δ 161.5, 147.9, 146.9, 137.5, 136.5, 131.8, 127.1, 126.2, 126.0, 125.0, 121.2, 120.3, 111.1, 68.9, 31.6, 29.3, 25.9, 22.7, 14.1; LRMS (ESI) *m/z* calcd for $C_{19}H_{22}ClN_2O_4$ $[M+H]^+$: 377.13; Found: 377.20.

Synthesis of compound 8, 2-chloro-*N*-(2-(2-(4-methylpiperazin-1-yl)ethoxy)phenyl)-5-nitrobenzamide

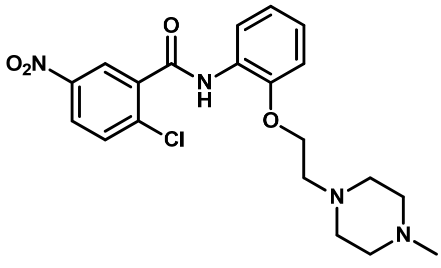

N-Boc-2-aminophenol (290 mg, 1.4 mmol), 4-methylpiperazine-1-ethanol (200 mg, 1.4 mmol), triphenylphosphine (550 mg, 2.1 mmol) were dissolved in anhydrous THF (20 mL) under argon atmosphere and diethyl azodicarboxylate (360 mg, 2.1 mmol) was added slowly. The reaction mixture was stirred at room temperature for 12 h, and concentrated under the reduced pressure. The residue was purified by silica-gel flash column chromatography (1:20 = methanol:dichloromethane, v/v) to obtain tert-butyl (2-(2-(4-methylpiperazin-1-yl)ethoxy)phenyl)carbamate. After Boc-deprotection with 10% trifluoroacetic acid in dichloromethane (10 mL) at room temperature for 1 h, the reaction mixture was washed with saturated aqueous $NaHCO_3$ three times, and concentrated under the reduced pressure to provide 2-(2-(4-methylpiperazin-1-yl)ethoxy)aniline. To a solution of 2-(2-(4-methylpiperazin-1-yl)ethoxy)aniline in dichloromethane (10 mL), 2-chloro-5-nitrobenzoyl chloride (460 mg, 2.1 mmol) and triethylamine (0.39 mL, 2.8 mmol) were added and the mixture was stirred at room temperature for 1 h. After removal of solvent and triethylamine under the reduced pressure, the residue was purified by silica-gel flash column chromatography (1:10 = MeOH:CH_2Cl_2, v/v) to obtain **8** (260 mg). Yield: 45% as light yellow solid; ^{1}H NMR (400 MHz, $CDCl_3$) δ 9.31 (brs, 1H), 8.64 (d, *J* = 2.8 Hz, 1H), 8.45 (td, *J* = 8.0, 1.6 Hz, 1H), 8.26 (td, *J* = 8.4, 2.8 Hz, 1H), 7.66 (d, *J* = 8.4 Hz, 1H), 7.13 (dd, *J* = 8.0, 1.6 Hz, 1H), 7.08 (dd, *J* = 8.0, 1.2 Hz, 1H), 6.98 (dd, *J* = 8.0, 1.2 Hz, 1H), 4.19 (t, *J* = 5.6 Hz, 2H), 2.72 (t, J = 5.6 Hz, 2H), 2.50–2.43 (m, 8H), 2.16 (s, 3H); ^{13}C NMR (100 MHz, $CDCl_3$) δ 161.9, 147.9, 146.5, 137.8, 137.0, 131.5, 128.0,

125.7, 125.5, 125.1, 121.9, 121.0, 113.2, 66.7, 57.0, 54.6, 53.1, 45.8; LRMS (ESI) *m/z* calcd for $C_{20}H_{24}ClN_4O_4$ $[M+H]^+$: 419.15; Found: 419.06.

Procedure for synthesis of compound 9 (SB1451)

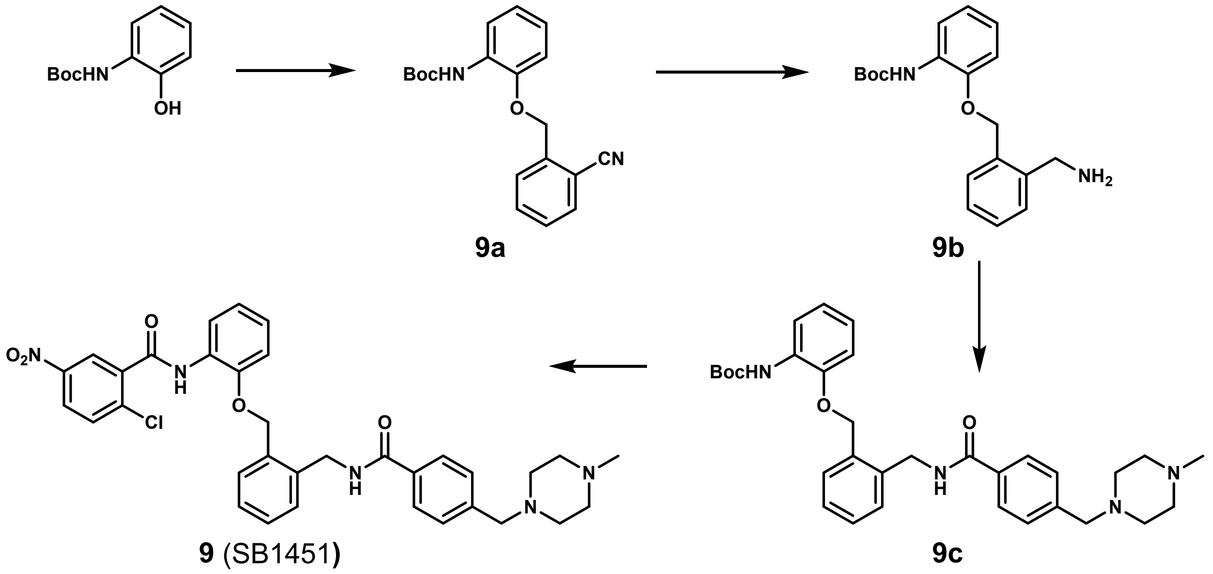

Synthesis of 9a, *tert*-butyl (2-((2-cyanobenzyl)oxy)phenyl)carbamate

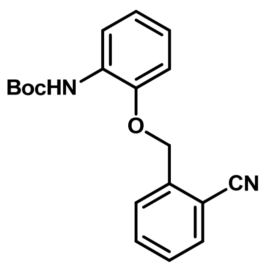

N-Boc-2-aminophenol (800 mg, 3.8 mmol), 2-(bromomethyl) benzonitrile (1.5 g, 7.6 mmol) and K_2CO_3 (790 mg, 5.7 mmol) were dissolved in anhydrous DMF (30 mL) under argon atmosphere. The reaction mixture was stirred at 80 °C for 12 h, and the crude mixture was diluted with deionized water. The aqueous layer was extracted three times with ethyl acetate. The combined organic layer was dried over anhydrous Na_2SO_4(s) and filtered through a celite-packed glass filter. The filtrate was concentrated under the reduced pressure and purified by silica-gel flash column chromatography (1:5 =

ethyl acetate:*n*-hexane, v/v) to obtain **9a** (1.23 g). Yield: 99% as off-white solid; ^{1}H NMR (400 MHz, $CDCl_3$) δ 8.09 (d, *J* = 6.8 Hz, 1H), 7.73 (d, *J* = 8.0 Hz, 1H), 7.64 (t, *J* = 7.6 Hz, 1H), 7.59 (d, *J* =7.2 Hz, 1H), 7.46 (t, *J* = 7.2 Hz, 1H), 7.09 (s, 1H), 7.00–6.92 (m, 2H), 6.89 (d, *J* = 8.0 Hz, 1H), 5.33 (s, 2H), 1.53 (s, 9H); ^{13}C NMR (100 MHz, $CDCl_3$) δ 152.9, 146.2, 140.2, 133.30, 133.25, 128.9, 128.7, 128.6, 122.4, 122.3, 119.0, 117.2, 111.9, 111.5, 80.6, 68.4, 28.5; LRMS (ESI) *m/z* calcd for $C_{19}H_{21}N_2O_3$ [M+H]$^{+}$: 325.16; Found: 325.25.

Synthesis of 9b, *tert*-butyl (2-((2-(aminomethyl)benzyl)oxy)phenyl)carbamate

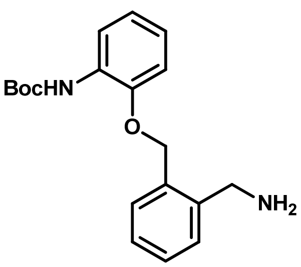

To a solution of **9a** (600 mg, 1.85 mmol) in anhydrous THF (15 mL) under argon atmosphere, $LiAlH_4$ (1.0 M in THF, 3.7ml) was added dropwise at 0 °C, and the mixture was warmed to room temperature. After 3 h, the reaction mixture was cooled to 0 °C and quenched with deionized water (3 mL). The crude mixture was worked up with ethyl acetate and saturated aqueous sodium potassium tartrate, and the organic layer was washed by brine. The combined organic layer was dried over anhydrous Na_2SO_4(s) and filtered through a celite-packed glass filter. The filtrate was concentrated under the reduced pressure and purified by silica-gel flash column chromatography (1:10 = MeOH:CH_2Cl_2, v/v) to obtain **9b** (360 mg). Yield: 59% as yellow oil; ^{1}H NMR (500 MHz, $CDCl_3$) δ 8.13 (s, 1H), 7.85 (s, 1H), 7.44–7.38 (m, 3H), 7.31 (t, *J* = 7.0 Hz, 1H), 7.02-6.96 (m, 3H), 5.15 (s, 2H), 3.96 (s, 2H), 1.77 (brs, 2H), 1.52 (s, 9H); ^{13}C NMR (100 MHz, $CDCl_3$) δ 153.1, 147.1, 140.4, 134.4, 129.9, 129.24, 129.20, 129.0, 127.7, 122.5, 121.9, 118.9, 112.5, 80.2, 69.6, 43.4, 28.5; LRMS (ESI) *m/z* calcd for $C_{19}H_{25}N_2O_3$ [M+H]$^{+}$: 329.19; Found:329.25.

Synthesis of 9c, *tert*-butyl (2-((2-((4-((4-methylpiperazin-1-yl)methyl)benzamido) methyl) benzyl)oxy)phenyl)carbamate

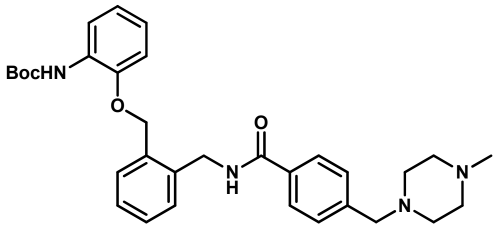

To a solution of **9b** (140 mg, 0.43 mmol) in dichloromethane (10 mL), 4-((4-methyl-1-piperazinyl)methyl)benzoic acid dihydrochloride (160 mg, 0.52 mmol), 1-ethyl-3-(3-dimethyl aminopropyl)carbodiimide hydrochloride (100 mg, 0.52 mmol), triethylamine (0.24 mL, 1.7 mmol), and 4-dimethylaminopyridine (5.3 mg, 0.043 mmol) were added, and the mixture was stirred at room temperature for 12 h. The crude mixture was worked up with dichloromethane and saturated aqueous $NaHCO_3$, and the organic layer was washed by brine. The combined organic layer was dried over anhydrous Na_2SO_4(s) and filtered through a celite-packed glass filter. The filtrate was concentrated under the reduced pressure and purified by silica-gel flash column chromatography (1:5 = MeOH:CH_2Cl_2, v/v) to obtain **9c** (170 mg). Yield: 74% as yellow oil; ^{1}H NMR (400 MHz, $CDCl_3$) δ 8.04 (s, 1H), 7.63 (d, J = 8.4 Hz, 2H), 7.46–7.34 (m, 4H), 7.33 (d, J = 9.2 Hz, 2H), 6.99 (s, 1H), 6.97–6.90 (m, 3H), 6.61 (t, J = 4.8 Hz, 1H), 5.18 (s, 2H), 4.73 (d, J = 5.6 Hz, 2H), 3.49 (s, 2H), 2,34 (brs, 8H), 2.29 (s, 3H), 1.47 (s, 9H); ^{13}C NMR (100 MHz, $CDCl_3$) δ 167.0, 152.8, 146.7, 142.4, 137.0, 134.7, 132.9, 130.0, 129.7, 120.24, 129.22, 128.5, 128.3, 127.0, 122.6, 122.0, 118.7, 112.3, 80.6, 69.3, 62.5, 55.1, 53.0, 46.0, 41.6, 28.4; LRMS (ESI) *m/z* calcd for $C_{32}H_{41}N_4O_4$ $[M+H]^+$: 545.31; Found:545.35.

Synthesis of 9 (SB1451), 2-chloro-*N*-(2-((2-((4-((4-methylpiperazin-1-yl)methyl) benzamido)methyl)benzyl)oxy)phenyl)-5-nitrobenzamide

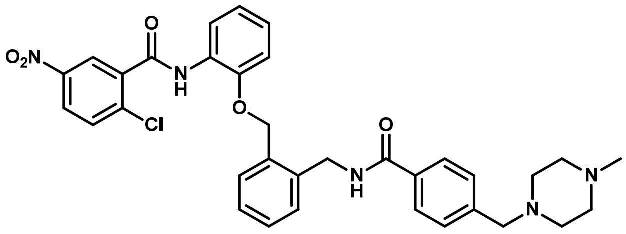

After **9c** (110 mg, 0.20 mmol) was treated with 10% trifluoroacetic acid in dichloromethane (10 mL) at room temperature for 1 h, the reaction mixture was washed with saturated aqueous $NaHCO_3$ three times, and concentrated under the reduced pressure to provide *N*-(2-((2-aminophenoxy)methyl)benzyl)-4-((4-methylpiperazin-1-yl)methyl)benzamide. To a solution of *N*-(2-((2-aminophenoxy)methyl)benzyl)-4-((4-methylpiperazin-1-yl)methyl)benzamide in dichloromethane (10 mL), 2-chloro-5-nitrobenzoyl chloride (66 mg, 0.30 mmol) and triethylamine (56 µL, 0.40 mmol) were added and the mixture was stirred at room temperature for 1 h. After removal of solvent and triethylamine under the reduced pressure, the residue was purified by silica-gel flash column chromatography (1:5 = MeOH:CH_2Cl_2, v/v) to obtain **9** (85 mg). Yield : 68% as light yellow solid; ^{1}H NMR (400 MHz, DMSO-*d*$_6$) δ 10.09 (s, 1H), 8.99 (t, *J* = 6.0 Hz, 1H), 8.38 (d, *J* = 2.8 Hz, 1H), 8.26 (dd, *J* = 8.8, 2.8 Hz, 1H), 7.87 (dd, *J* = 7.6, 1.2 Hz, 1H), 7.80 (d, *J* = 9.2, 1H), 7.75 (d, *J* = 7.6, 2H), 7.55 (d, *J* = 7.6, 1H), 7.39–7.20 (m, 7H), 7.01 (t, *J* = 7.6 Hz, 1H), 5.34 (s, 2H), 4.59 (d, *J* = 5.6 Hz, 2H), 3.84 (s, 2H), 2.34 (brs, 8H), 2.15 (s, 3H); ^{13}C NMR (100 MHz, DMSO-*d*$_6$) δ 166.1, 163.1, 150.5, 146.0, 141.8, 137.9, 137.7, 137.2, 134.2, 132.8, 131.3, 128.5, 128.2, 128.1, 127.1, 126.8, 126.29, 126.26, 125.5, 124.7, 124.0, 120.5, 113.1, 67.9, 61.6, 54.7, 52.5, 45.7; LRMS (ESI) *m/z* calcd for $C_{34}H_{35}ClN_5O_5$ $[M+H]^+$: 628.23; Found: 628.30.

Procedure for synthesis of compound 10 (SB1453)

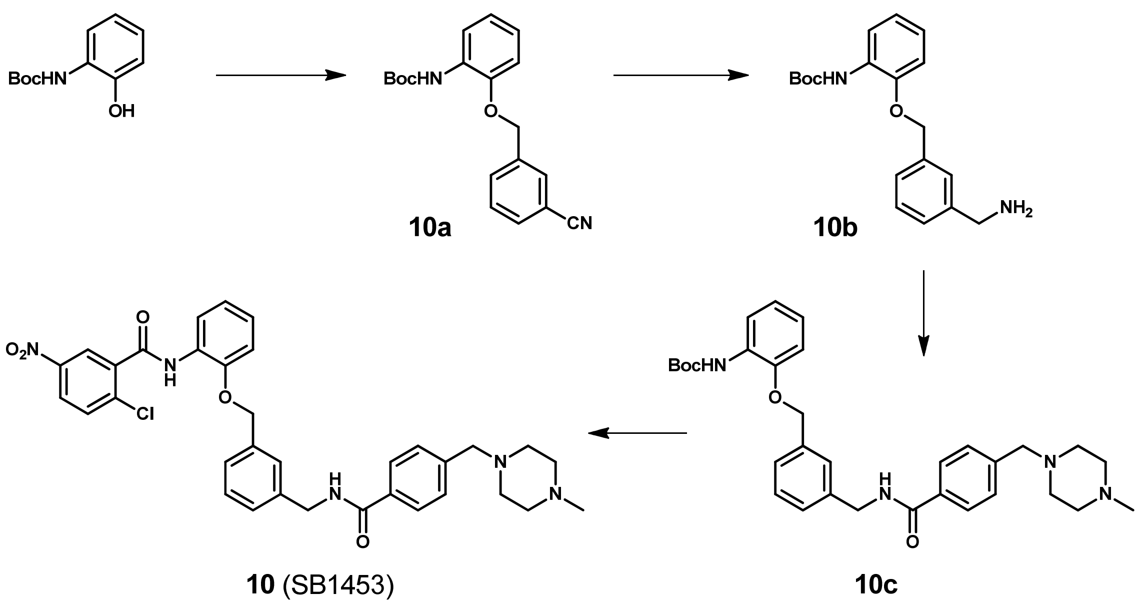

Synthesis of 10a, *tert*-butyl (2-((3-cyanobenzyl)oxy)phenyl)carbamate

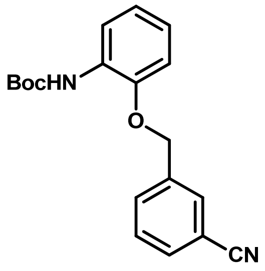

N-Boc-2-aminophenol (800 mg, 3.8 mmol), 3-(bromomethyl) benzonitrile (1.5 g, 7.6 mmol) and K_2CO_3 (790 mg, 5.7 mmol) were dissolved in anhydrous DMF (30 mL) under argon atmosphere. The reaction mixture was stirred at 80 °C for 12 h, and the crude mixture was diluted with deionized water. The aqueous layer was extracted three times with ethyl acetate. The combined organic layer was dried over anhydrous Na_2SO_4(s) and filtered through a celite-packed glass filter. The filtrate was concentrated under the reduced pressure and purified by silica-gel flash column chromatography (1:5 = ethyl acetate:*n*-hexane, v/v) to obtain **10a** (1.20 g). Yield: 97% as yellow oil; ^{1}H NMR (400 MHz, $CDCl_3$) δ 8.10 (d, *J* = 7.2 Hz, 1H), 7.71 (s, 1H), 7.64 (d, *J* =8.0 Hz, 1H), 7.52 (t, *J* = 8.0 Hz, 1H), 7.02 (s, 1H), 7.00–6.91 (m, 2H), 6.83 (dd, *J* = 7.6, 1.2 Hz, 1H), 5.14 (s, 2H), 1.53 (s, 9H); ^{13}C NMR (100 MHz, $CDCl_3$) δ 152.8, 146.3, 138.3, 132.0,

131.7, 130.9, 129.7, 128.5, 122.5, 122.1, 118.9, 118.6, 113.1, 111.7, 80.7, 69.6, 28.5; LRMS (ESI) *m/z* calcd for $C_{19}H_{21}N_2O_3$ $[M+H]^+$: 325.16; Found:325.20.

Synthesis of 10b, *tert*-butyl (2-((3-(aminomethyl)benzyl)oxy)phenyl)carbamate

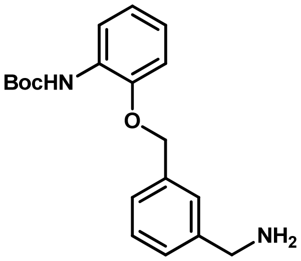

To a solution of **10a** (600 mg, 1.85 mmol) in anhydrous THF (15 mL) under argon atmosphere, $LiAlH_4$ (1.0 M in THF, 3.7 mL) was added dropwise at 0 °C, and the mixture was warmed to room temperature. After 3 h, the reaction mixture was cooled to 0 °C and quenched with deionized water (3 mL). The crude mixture was worked up with ethyl acetate and saturated aqueous sodium potassium tartrate, and the organic layer was washed by brine. The combined organic layer was dried over anhydrous Na_2SO_4(s) and filtered through a celite-packed glass filter. The filtrate was concentrated under the reduced pressure and purified by silica-gel flash column chromatography (1:10 = MeOH:CH_2Cl_2, v/v) to obtain **10b** (440 mg). Yield: 72% as off-white solid; ^1H NMR (400 MHz, $CDCl_3$) δ 8.07 (brs, 1H), 7.40 (s, 1H), 7.38–7.29 (m, 3H), 7.12 (s, 1H), 6.97–6.87 (m, 3H), 5.08 (s, 2H), 3.91 (s, 2H), 2.81 (brs, 2H), 1.51 (s, 9H); ^{13}C NMR (100 MHz, $CDCl_3$) δ 152.9, 146.8, 137.1, 129.1, 128.4, 127.4, 126.6, 126.4, 122.5, 121.6, 118.6, 111.8, 80.5, 70.8, 45.9, 28.5; LRMS (ESI) *m/z* calcd for $C_{19}H_{25}N_2O_3$ $[M+H]^+$: 329.19; Found: 329.22.

Synthesis of 10c, *tert*-butyl (2-((3-((4-((4-methylpiperazin-1-yl)methyl)benzamido) methyl)benzyl)oxy)phenyl)carbamate

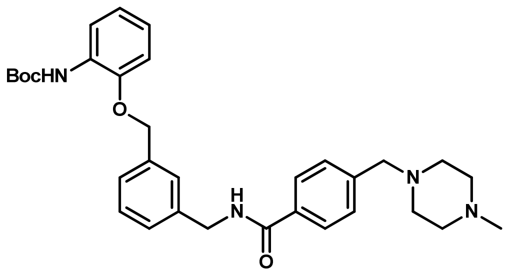

To a solution of **10b** (140 mg, 0.43 mmol) in dichloromethane (10 mL), 4-((4-methyl-1-piperazinyl)methyl)benzoic acid dihydrochloride (160 mg, 0.52 mmol), 1-ethyl-3-(3-dimethyl aminopropyl)carbodiimide hydrochloride (100 mg, 0.52 mmol), triethylamine (0.24 mL, 1.7 mmol), and 4-dimethylaminopyridine (5.3 mg, 0.043 mmol) were added, and the mixture was stirred at room temperature for 12 h. The crude mixture was worked up with dichloromethane and saturated aqueous $NaHCO_3$, and the organic layer was washed by brine. The combined organic layer was dried over anhydrous Na_2SO_4(s) and filtered through a celite-packed glass filter. The filtrate was concentrated under the reduced pressure and purified by silica-gel flash column chromatography (1:5 = MeOH:CH_2Cl_2, v/v) to obtain **10c** (230mg). Yield: 98% as yellow oil; ^{1}H NMR (400 MHz, $CDCl_3$) δ 8.05 (s, 1H), 7.75 (d, *J* = 8.0 Hz, 2H), 7.38 (d, *J* = 8.0 Hz, 2H), 7.36–7.33 (m, 4H), 7.07 (brs, 1H), 6.93–6.86 (m, 3H), 6.66 (s, 1H), 5.09 (s, 2H), 4.65 (d, *J* = 5.6 Hz, 2H), 3.54 (s, 2H), 2.50 (brs, 8H), 2.31 (s, 3H), 1.53 (s, 9H); ^{13}C NMR (100 MHz, $CDCl_3$) δ 167.4, 152.9, 146.7, 142.1, 139.0, 137.2, 133.2, 129.3, 128.5, 127.9, 127.1, 127.0, 126.8, 122.5, 121.6, 118.6, 112.0, 80.6, 70.8, 62.5, 55.0, 52.9, 45.9, 44.0, 28.4; LRMS (ESI) *m/z* calcd for $C_{32}H_{41}N_4O_4$ $[M+H]^+$: 545.31; Found: 545.40.

Synthesis of 10 (SB1453), 2-chloro-*N*-(2-((3-((4-((4-methylpiperazin-1-yl)methyl) benzamido)methyl)benzyl)oxy)phenyl)-5-nitrobenzamide

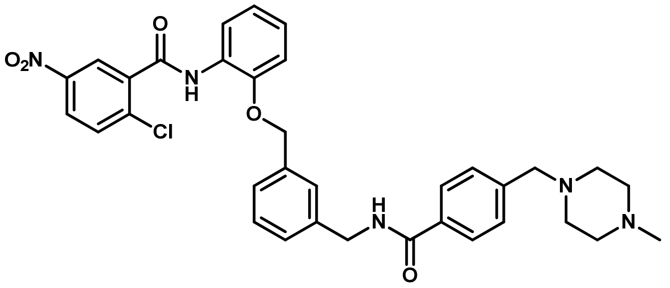

After **10c** (110 mg, 0.20 mmol) was treated with 10% trifluoroacetic acid in dichloromethane (10 mL) at room temperature for 1 h, the reaction mixture was washed with saturated aqueous $NaHCO_3$ three times, and concentrated under the reduced pressure to provide *N*-(3-((2-aminophenoxy)methyl)benzyl)-4-((4-methylpiperazin-1-yl)methyl)benzamide. To a solution of *N*-(3-((2-aminophenoxy)methyl)benzyl)-4-((4-methylpiperazin-1-yl)methyl) benzamide in dichloromethane (10 mL), 2-chloro-5-nitrobenzoyl chloride (66 mg, 0.30 mmol) and triethylamine (56 μL, 0.40 mmol) were added and the mixture was stirred at room temperature for 1 h. After removal of solvent and triethylamine under the reduced pressure, the residue was purified by silica-gel flash column chromatography (1:5 = MeOH:CH_2Cl_2, v/v) to obtain **10** (102 mg). Yield: 81% as light yellow solid; ^1H NMR (400 MHz, DMSO-d_6) δ 10.07 (s, 1H), 8.98 (t, *J* = 6.0 Hz, 1H), 8.37 (d, *J* = 2.8 Hz, 1H), 8.29 (dd, *J* = 8.4, 2.8 Hz, 1H), 7.84–7.81 (m, 4H), 7.43–7.31 (m, 5H), 7.26 (d, *J* = 7.6 Hz, 1H), 7.15 (t, *J* = 5.2 Hz, 2H), 7.01 (t, *J* = 7.6 Hz, 1H), 5.19 (s, 2H), 4.47 (d, *J* = 6.0 Hz, 2H), 3.48 (s, 2H), 2.34 (brs, 8H), 2.14 (s, 3H); ^{13}C NMR (100 MHz, DMSO-d_6) δ 166.0, 163.0, 150.6, 146.0, 141.8, 139.9, 137.7, 137.2, 137.0, 133.0, 131.3, 128.6, 128.4, 127.2, 126.6. 126.32, 126.27, 125.9, 125.6, 124.7, 124.1, 120.5, 113.2, 69.8, 61.6, 54.7, 52.6, 45.7, 42.5; LRMS (ESI) *m/z* calcd for $C_{34}H_{35}ClN_5O_5$ $[M+H]^+$: 628.23; Found: 628.41.

Procedure for synthesis of compound 11

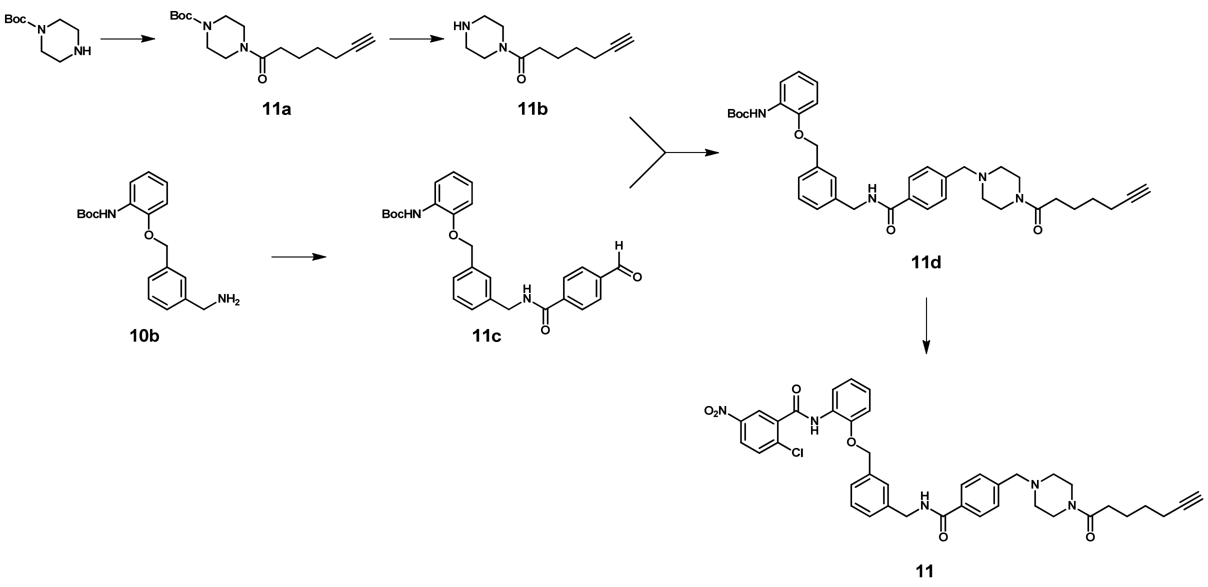

Synthesis of 11a, *tert*-butyl 4-(hept-6-ynoyl)piperazine-1-carboxylate

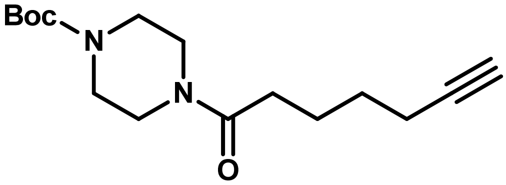

To a solution of 1-Boc-piperazine (1.00 g, 5.37 mmol) in dichloromethane (50 mL), 6-heptynoic acid (722 mg, 6.44 mmol), 1-ethyl-3-(3-dimethylaminopropyl)carbodiimide hydrochloride (1.23 g, 6.44 mmol), triethylamine (0.897 mL, 6.44 mmol), and 4-dimethylaminopyridine (65.6mg, 0.537 mmol) were added, and the mixture was stirred at room temperature for 12 h. The crude mixture was worked up with dichloromethane and saturated aqueous NaHCO3, and the organic layer was washed by brine. The combined organic layer was dried over anhydrous Na_2SO_4(s) and filtered through a celite-packed glass filter. The filtrate was concentrated under the reduced pressure and purified by silica-gel flash column

chromatography (1:10 = MeOH:CH_2Cl_2, v/v) to obtain **11a** (1.50 g). Yield: 95% as light yellow solid; ^{1}H NMR (400 MHz, $CDCl_3$) δ 3.59 (t, *J* = 4.8 Hz, 2H), 3.45 (s, 4H), 3.40 (t, *J* = 4.8 Hz, 2H), 2.36 (t, *J* = 7.2 Hz, 2H), 2.23 (td, *J* = 7.2, 2.4 Hz, 2H), 1.95 (t, *J* = 2.8 Hz, 1H), 1.77 (p, *J* = 7.2 Hz, 2H), 1.59 (p, *J* = 7.2 Hz, 2H), 1.47 (s, 9H); ^{13}C NMR (100 MHz, $CDCl_3$) δ 171.4, 154.8, 84.1, 80.4, 68.7, 45.5, 41.5, 32.9, 28.5, 28.1, 24.3, 18.3; LRMS (ESI) *m/z* calcd for $C_{16}H_{27}N_2O_3$ [M+H]$^+$: 295.20; Found: 295.20.

Synthesis of 11b, 1-(piperazin-1-yl)hept-6-yn-1-one

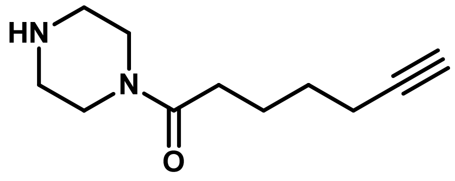

After **11a** (1.00 g, 3.40 mmol) was treated with 10% trifluoroacetic acid in dichloromethane (50 mL) at room temperature for 12 h, the reaction mixture was washed with saturated aqueous $NaHCO_3$ three times, and concentrated under the reduced pressure to obtain **11** (530 mg). Yield: 80% as yellow oil; ^{1}H NMR (400 MHz, $CDCl_3$) ^{1}H NMR (400 MHz, $CDCl_3$) δ 3.60 (t, *J* = 4.8 Hz, 2H), 3.46 (t, *J* = 4.8 Hz, 2H), 2.86 (m, 4H), 2.74(brs, 1H), 2.35 (t, *J* = 7.2 Hz, 2H), 2.23 (td, *J* = 7.2, 2.8 Hz 2H), 1.95 (t, *J* = 2.4 Hz, 1H), 1.76 (p, *J* = 7.2 Hz, 2H), 1.59 (p, *J* = 7.2 Hz, 2H); ^{13}C NMR (100 MHz, $CDCl_3$) δ 171.1, 84.0, 68.6, 46.6, 46.2, 45.7, 42.4, 32.6, 28.0, 24.3, 18.2; LRMS (ESI) *m/z* calcd for $C_{11}H_{19}N_2O$ [M+H]$^+$: 195.15; Found: 195.20.

Synthesis of 11c, *tert*-butyl (2-((3-((4-formylbenzamido)methyl)benzyl)oxy)phenyl) carbamate

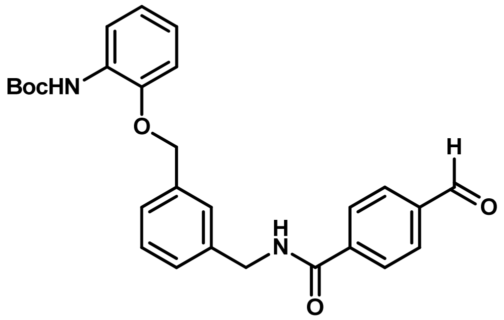

To a solution of **10b** (120 mg, 0.36 mmol) in dichloromethane (10 mL), 4-formyl benzoic acid (66 mg, 0.44 mmol), 1-ethyl-3-(3-dimethyl aminopropyl)carbodiimide hydrochloride (84 mg, 0.44 mmol), triethylamine (0.10 mL, 0.73 mmol), and 4-dimethylaminopyridine (4.5 mg, 0.036 mmol) were added, and the mixture was stirred at room temperature for 12 h. The crude mixture was worked up with dichloromethane and saturated aqueous $NaHCO_3$, and the organic layer was washed by brine. The combined organic layer was dried over anhydrous Na_2SO_4(s) and filtered through a celite-packed glass filter. The filtrate was concentrated under the reduced pressure and purified by silica-gel flash column chromatography (1:1 = ethyl acetate:*n*-hexane, v/v) to obtain **11c** (72 mg). Yield: 43% as off-white solid; ^{1}H NMR (400 MHz, $CDCl_3$) δ 10.03 (s, 1H), 8.01 (s, 1H), 7.93 (d, J = 8.4 Hz, 2H), 7.89 (d, J = 8.4 Hz, 2H), 7.37–7.31 (m, 4H), 7.03 (s, 1H), 6.96–6.86 (m, 4H), 5.07 (s, 2H), 4.63 (d, J = 5.6 Hz, 2H), 1.48 (s, 9H); ^{13}C NMR (100 MHz, $CDCl_3$) δ 191.6, 166.4, 152.9, 146.8, 139.5, 138.5, 138.3, 137.2, 129.9, 129.3, 128.5, 127.93, 127.88, 127.1, 127.0, 122.6, 121.7, 118.7, 112.1, 80.6, 70.9, 44.2, 28.4; LRMS (ESI) *m/z* calcd for $C_{27}H_{29}N_2O_5$ $[M+H]^+$: 461.21; Found: 461.30.

Synthesis of 11d, *tert*-butyl (2-((3-((4-((4-(hept-6-ynoyl)piperazin-1-yl)methyl) benzamido)methyl)benzyl)oxy)phenyl)carbamate

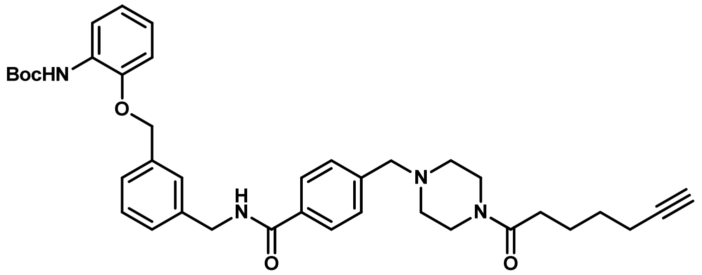

To a solution of **11b** (46 mg, 0.23 mmol) in dichloromethane (10 mL), **11c** (72 mg, 0.16 mmol), and sodium triacetoxyborohydride (33 mg, 0.16 mmol) were added, and the mixture was stirred under argon atmosphere at room temperature for 12 h. The crude mixture was worked up with dichloromethane and saturated aqueous $NaHCO_3$, and the organic layer was washed by brine. The combined organic layer was dried over anhydrous Na_2SO_4(s) and filtered through a celite-packed glass filter. The filtrate was concentrated under the reduced pressure and purified by silica-gel flash column chromatography (1:10 = MeOH:CH_2Cl_2, v/v) to obtain **11d** (95 mg). Yield: 95% as light yellow solid; ^{1}H NMR (400 MHz, $CDCl_3$) δ 8.05 (s, 1H), 7.77 (d, *J* = 7.6 Hz, 2H), 7.39–7.32 (m, 6H), 7.06 (s, 1H), 6.92–6.85 (m, 3H), 6.82 (t, *J* = 5.2 Hz, 1H), 5.08 (s, 2H), 4.64 (d, *J* = 5.2 Hz, 2H), 3.60 (brs, 2H), 3.54 (s, 2H), 3.45 (brs, 2H), 2.42–2.38 (m, 4H), 2.32 (t, *J* = 7.6 Hz, 2H), 2.20 (td, *J* = 7.2, 2.8 Hz, 2H), 1.94 (t, *J* = 2.8 Hz, 1H), 1.74 (p, *J* = 7.2 Hz, 2H), 1.56 (p, *J* = 7.2 Hz, 2H), 1.50 (s, 9H); ^{13}C NMR (100 MHz, $CDCl_3$) δ 171.2, 167.2, 152.8, 146.7, 141.7, 139.0, 137.1, 133.3, 129.17, 129.13, 128.5, 127.7, 127.2, 127.0, 126.7, 126.6, 122.4, 121.6, 118.5, 111.9, 84.1, 80.5, 70.8, 68.7, 62.4, 53.2, 52.8, 45.6, 43.9, 41.5, 32.7, 28.4, 28.1, 24.3, 18.2; LRMS (ESI) *m/z* calcd for $C_{38}H_{47}N_4O_5$ [M+H]$^+$: 639.35; Found: 639.40.

Synthesis of 11, 2-chloro-*N*-(2-((3-((4-((4-(hept-6-ynoyl)piperazin-1-yl)methyl) benzamido)methyl)benzyl)oxy)phenyl)-5-nitrobenzamide

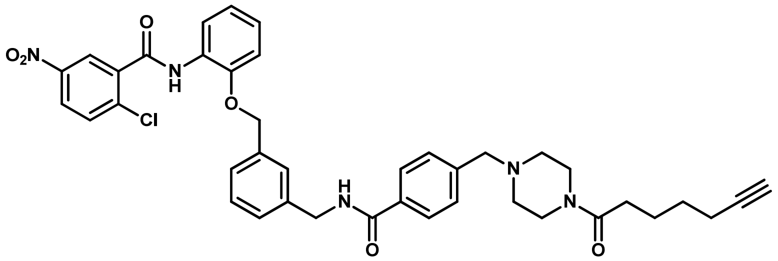

After **11d** (95 mg, 0.15 mmol) was treated with 10% trifluoroacetic acid in DCM (10 mL) at room temperature for 1 h, the reaction mixture was washed with saturated aqueous $NaHCO_3$ three times, and concentrated under the reduced pressure to provide *N*-(3-((2-aminophenoxy)methyl)benzyl)-4-((4-(hept-6-ynoyl)piperazin-1-yl)methyl)benzamide. To a solution of *N*-(3-((2-aminophenoxy)methyl)benzyl)-4-((4-(hept-6-ynoyl)piperazin-1-yl)methyl)benzamide in dichloromethane (10 mL), 2-chloro-5-nitrobenzoyl chloride (39 mg, 0.18 mmol) and triethylamine (41 μL, 0.29 mmol) were added and the mixture was stirred at room temperature for 1 h. After removal of solvent and triethylamine under the reduced pressure, the residue was purified by silica-gel flash column chromatography (1:5 = MeOH:CH_2Cl_2, v/v) to obtain **11** (101 mg). Yield: 94% as light yellow solid; ^{1}H NMR (500 MHz, $CDCl_3$) δ 8.71 (s, 1H), 8.63 (d, *J* = 2.0 Hz, 1H), 8.48 (dd, *J* = 8.0, 1.5 Hz, 1H), 8.18 (dd, *J* = 8.5, 2.5 Hz, 1H), 7.73 (d, *J* = 8.5 Hz, 2H), 7.55 (d, *J* = 9.0 Hz, 1H),7.40–7.33 (m, 6H), 7.12 (td, *J* = 7.5, 1.5 Hz, 1H), 7.07–7.00 (m, 2H), 6.54 (t, *J* = 6.0 Hz, 1H), 5.13 (s, 2H), 4.65 (d, *J* = 6.0 Hz, 2H), 3.62 (brs, 2H), 3.56 (s, 2H), 3.47 (brs, 2H), 2.42 (brs, 4H), 2.33 (t, *J* = 7.5 Hz, 2H), 2.22 (td, *J* = 7.0, 2.5 Hz, 2H), 1.94 (t, *J* = 2.5 Hz, 1H), 1.75 (p, *J* = 7.5 Hz, 2H), 1.58 (p, *J* = 7.5 Hz, 2H); ^{13}C NMR (125 MHz, $CDCl_3$) δ 171.2, 167.2, 161.6, 147.8, 146.8, 139.2, 137.6, 136.7, 136.3, 131.9, 129.4, 129.3, 128.1, 127.4, 127.2, 126.0, 125.2, 121.8, 120.8, 112.0,

84.2, 71.1, 68.7, 62.4, 53.3, 52.9, 45.6, 43.9, 41.8, 32.8, 28.2, 24.4, 18.3; LRMS (ESI) *m/z* calcd for $C_{40}H_{41}ClN_5O_6$ $[M+H]^+$: 722.27; Found: 722.25.

7. Supplementary References

1 Y. Amano, T. Yamaguchi, T. Ohno, T. Niimi, M. Orita, H. Sakashita and M.Takeuchi, *Hypertens. Res.*, 2012, **35**, 715–719.

2 A. T. Brünger, *Nature,* 1992, **355**, 472–474.

3 V. B. Chen, W. B. 3rd Arendall, J. J. Headd, D. A. Keddy, R. M. Immormino, G. j. Kapral, L. W. Murray and D. C. Richardson, *Acta Cryst. D,* 2010, **66**, 12–21.

4 P. Emsley, B. Lohkamp, W.G. Scott and K. Cowtan, *Acta Cryst. D,* 2010, **66**, 486–501.

5 G. N. Murshudov, A. A. Vagin & E. J. Dodson, *Acta Cryst. D,* 1997, **53**, 240–255.

6 Z. Otwinowski and W. Minor, *Enzymol.,* 1997, **276**, 307–326.

7 A. Vagin and A. Teplyakov, *Acta Cryst. D,* 2010, **66**, 22–25.

8. ^{1}H and ^{13}C NMR spectra

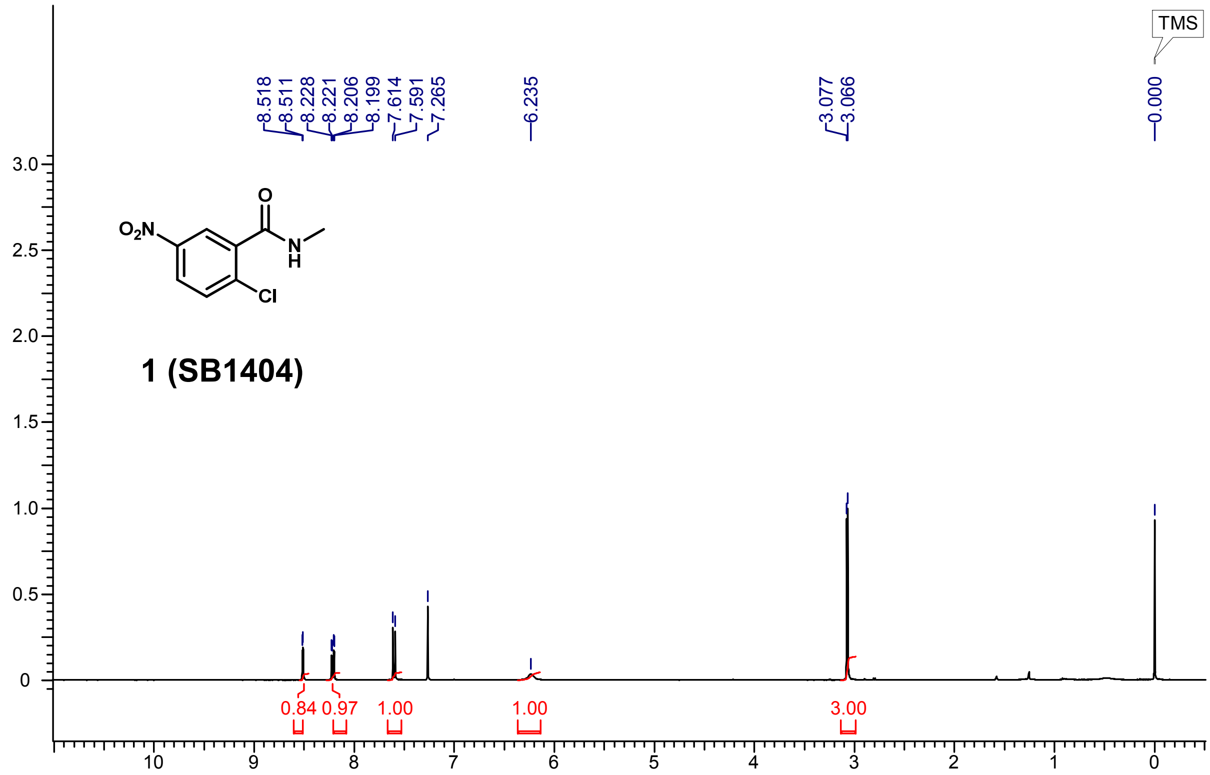

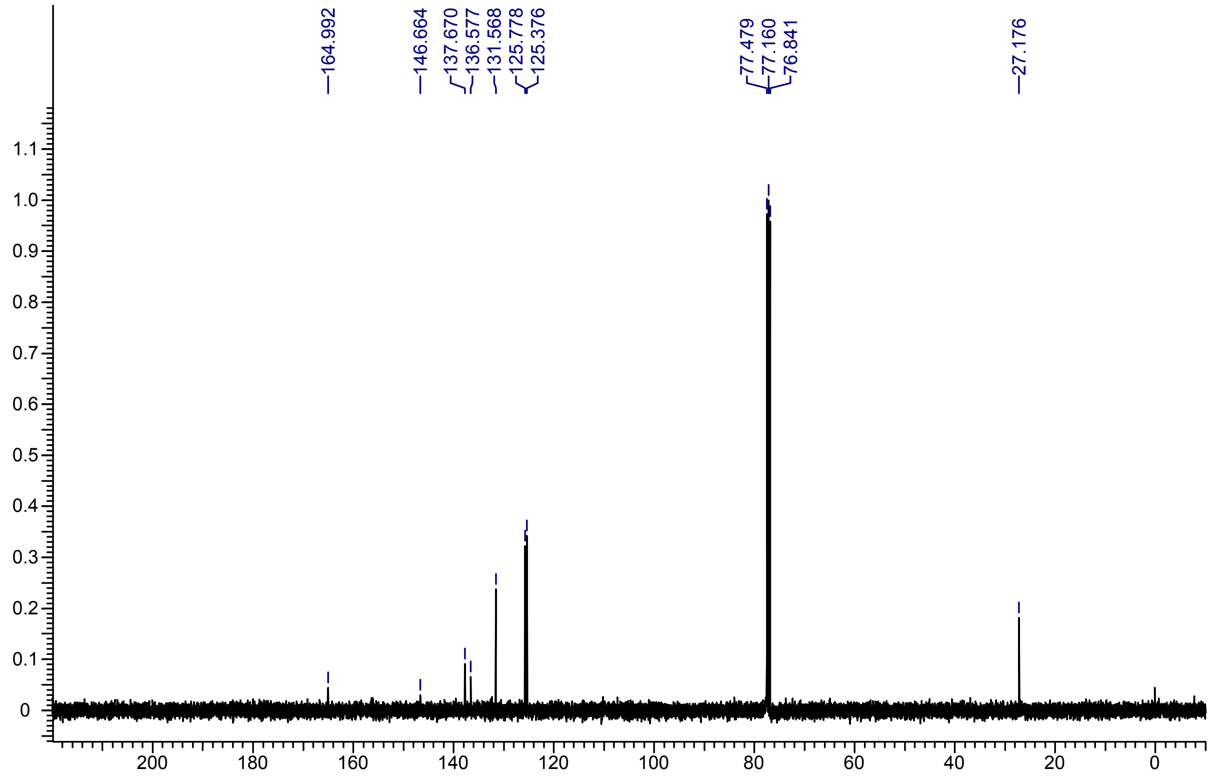

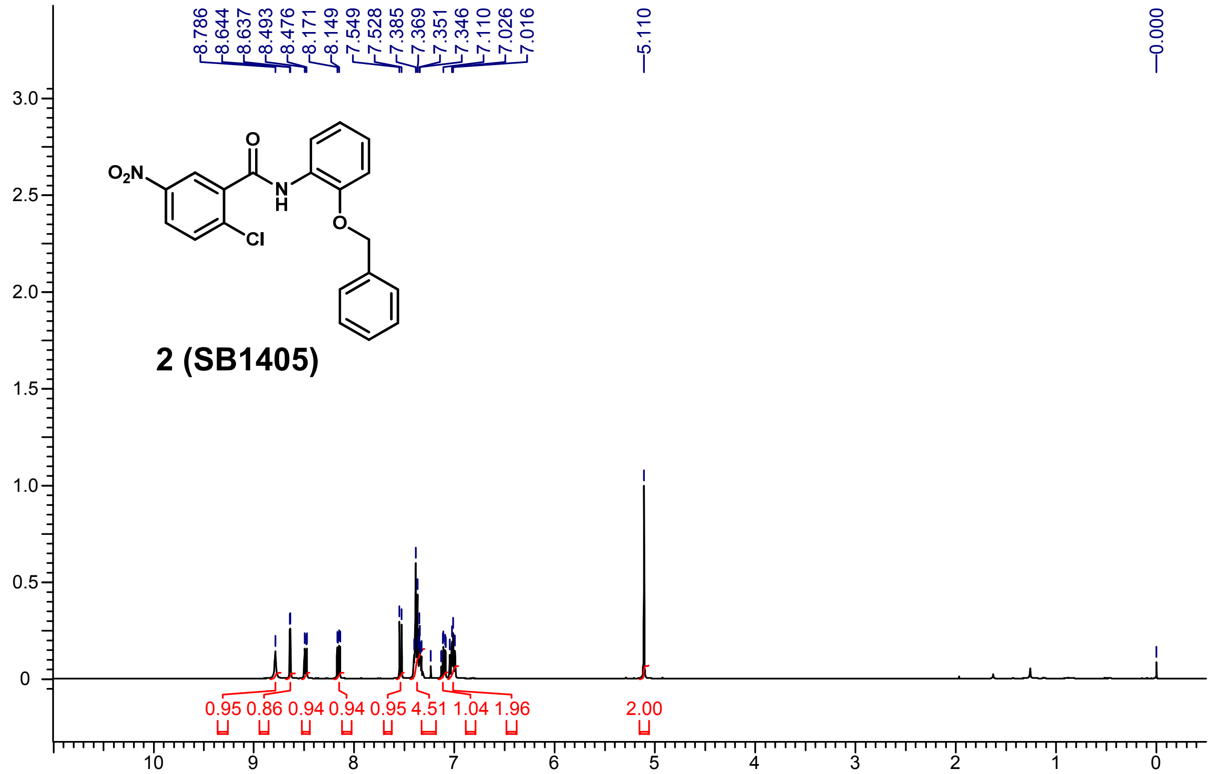
8.786
8.644
8.637
8.493
8.476
8.171
8.149
7.549
7.528
7.385
7.369
7.351
7.346
7.110
7.026
7.016
5.110
0.000
O
O_2N
N
H
Cl
O
2 (SB1405)
0.95 0.86 0.94 0.94 0.95 4.51 1.04 1.96
2.00

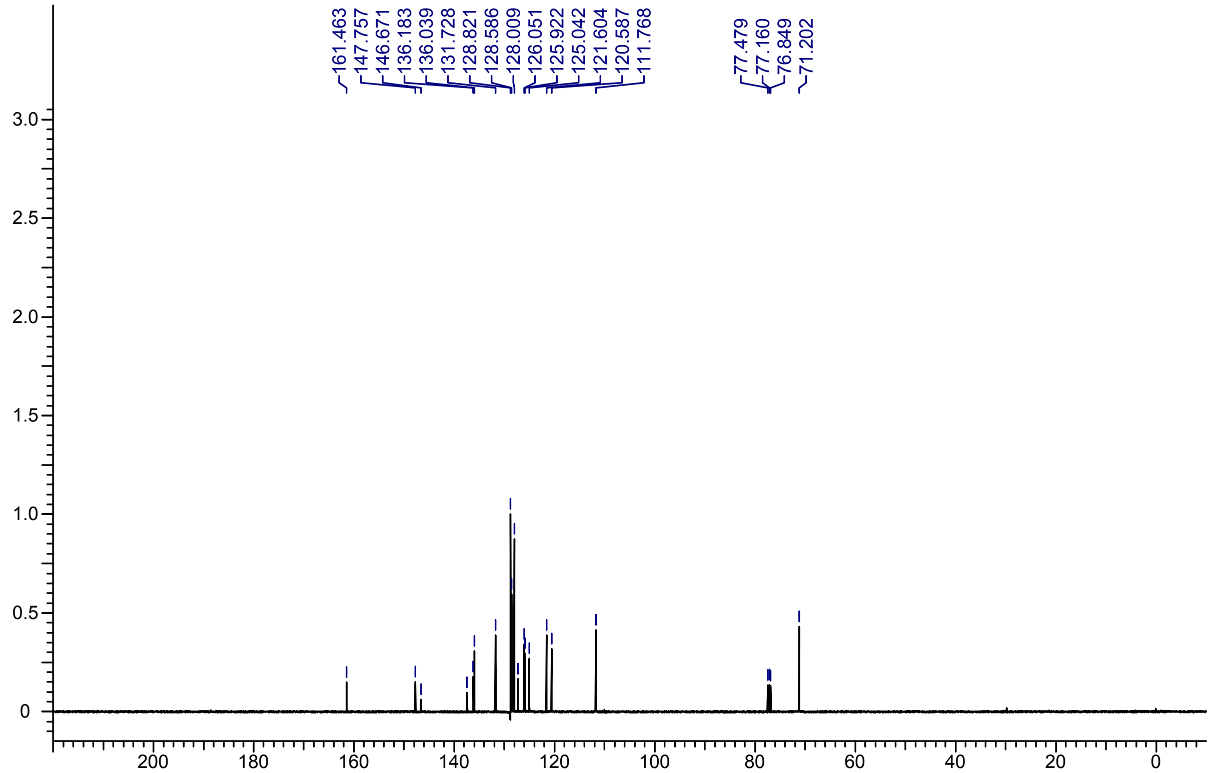
161.463
147.757
146.671
136.183
136.039
131.728
128.821
128.586
128.009
126.051
125.922
125.042
121.604
120.587
111.768
77.479
77.160
76.849
71.202

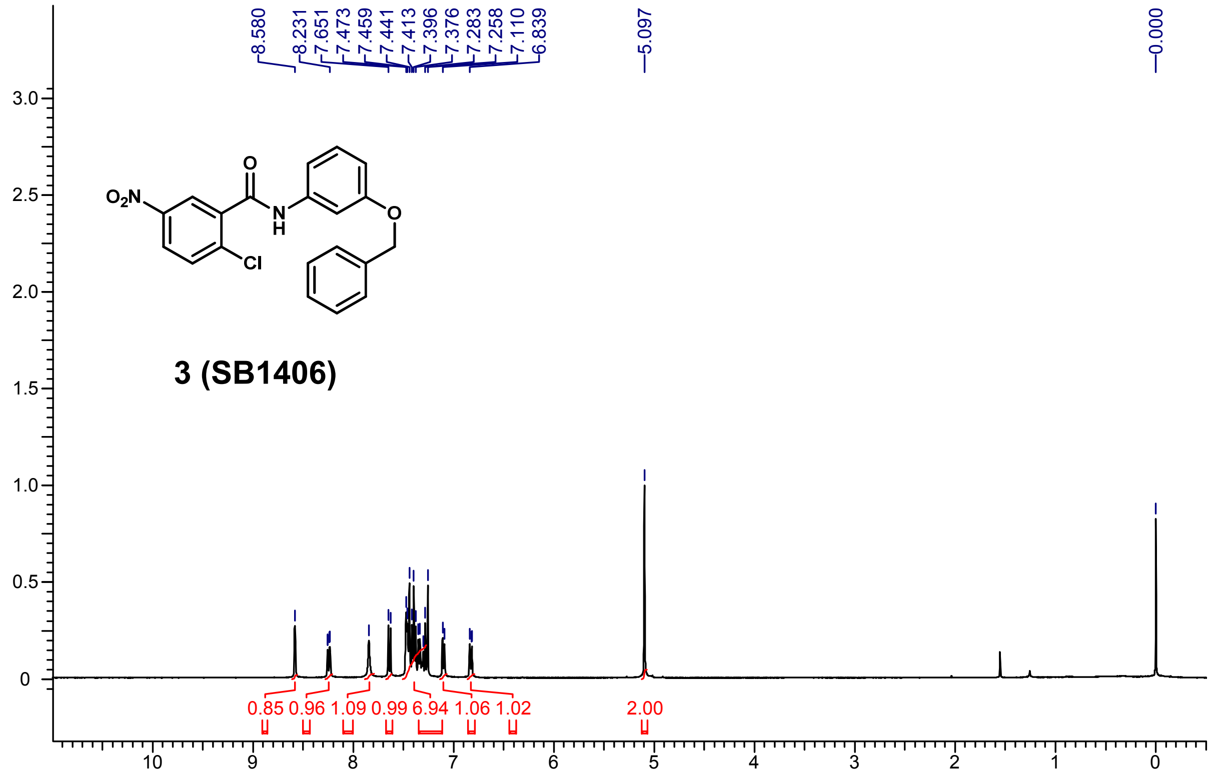
8.580
8.231
7.651
7.473
7.459
7.441
7.413
7.396
7.376
7.283
7.258
7.110
6.839
5.097
0.000
3 (SB1406)
0.85
0.96
1.09
0.99
6.94
1.06
1.02
2.00

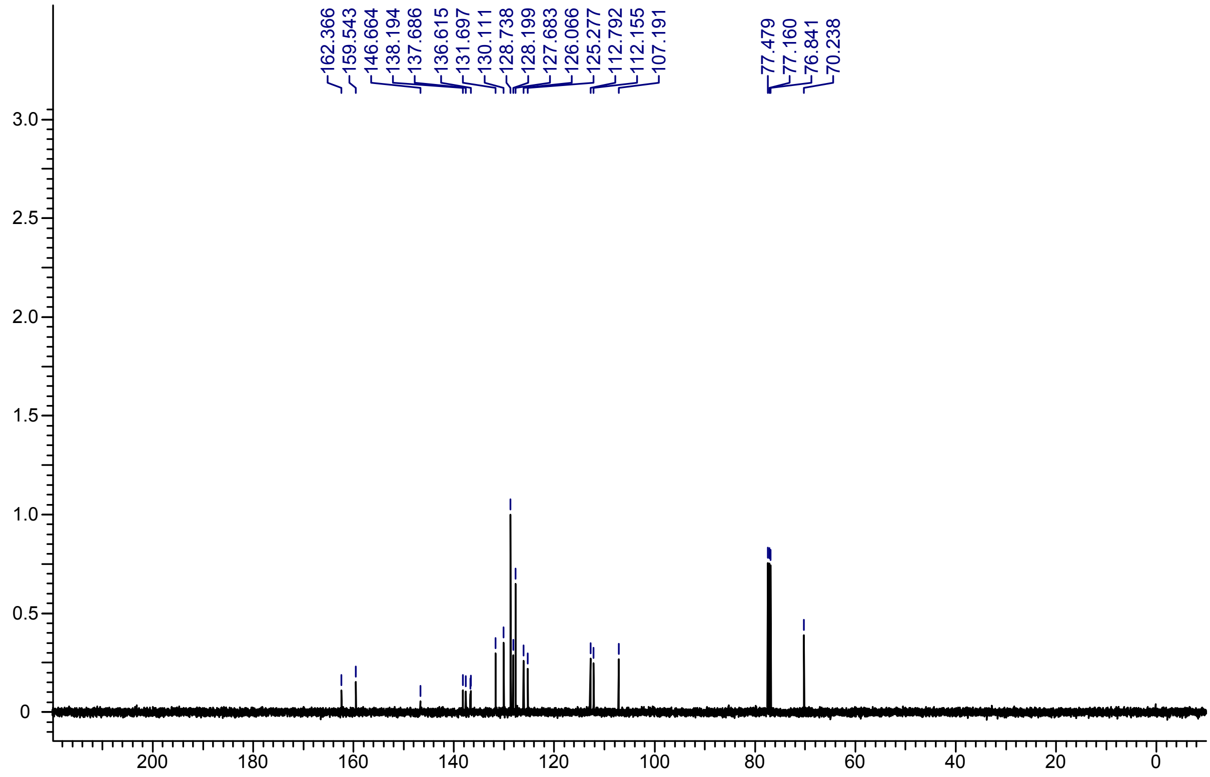
162.366
159.543
146.664
138.194
137.686
136.615
131.697
130.111
128.738
128.199
127.683
126.066
125.277
112.792
112.155
107.191
77.479
77.160
76.841
70.238

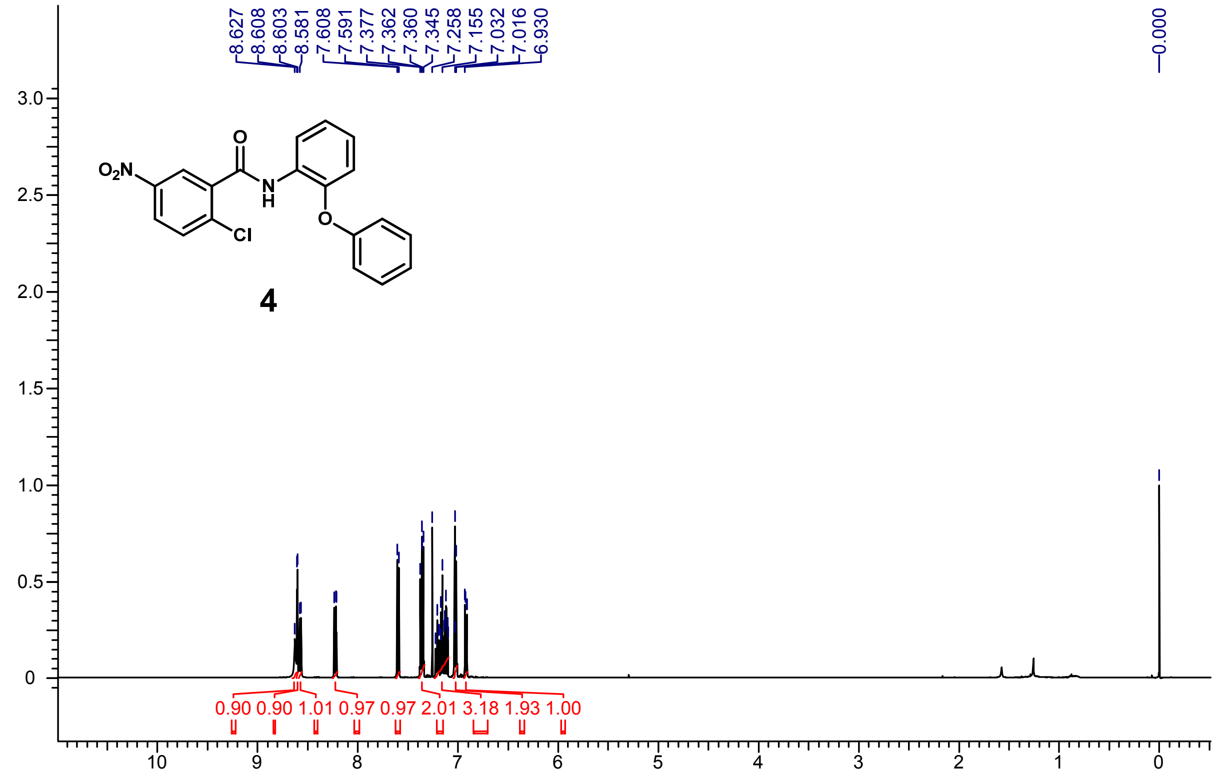

8.627
8.608
8.603
8.581
7.608
7.591
7.377
7.362
7.360
7.345
7.258
7.155
7.032
7.016
6.930
0.000
O
O_2N
N
H
Cl
O
4
0.90 0.90 1.01 0.97 0.97 2.01 3.18 1.93 1.00

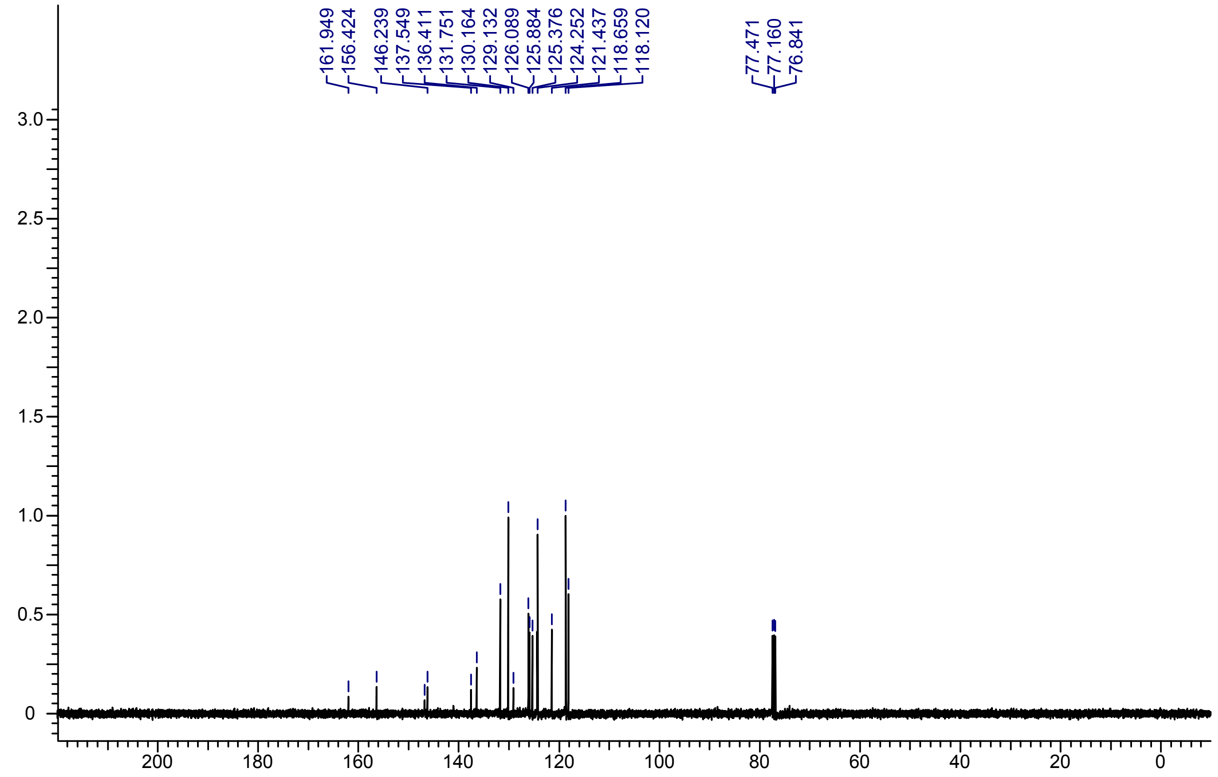

161.949
156.424
146.239
137.549
136.411
131.751
130.164
129.132
126.089
125.884
125.376
124.252
121.437
118.659
118.120
77.471
77.160
76.841

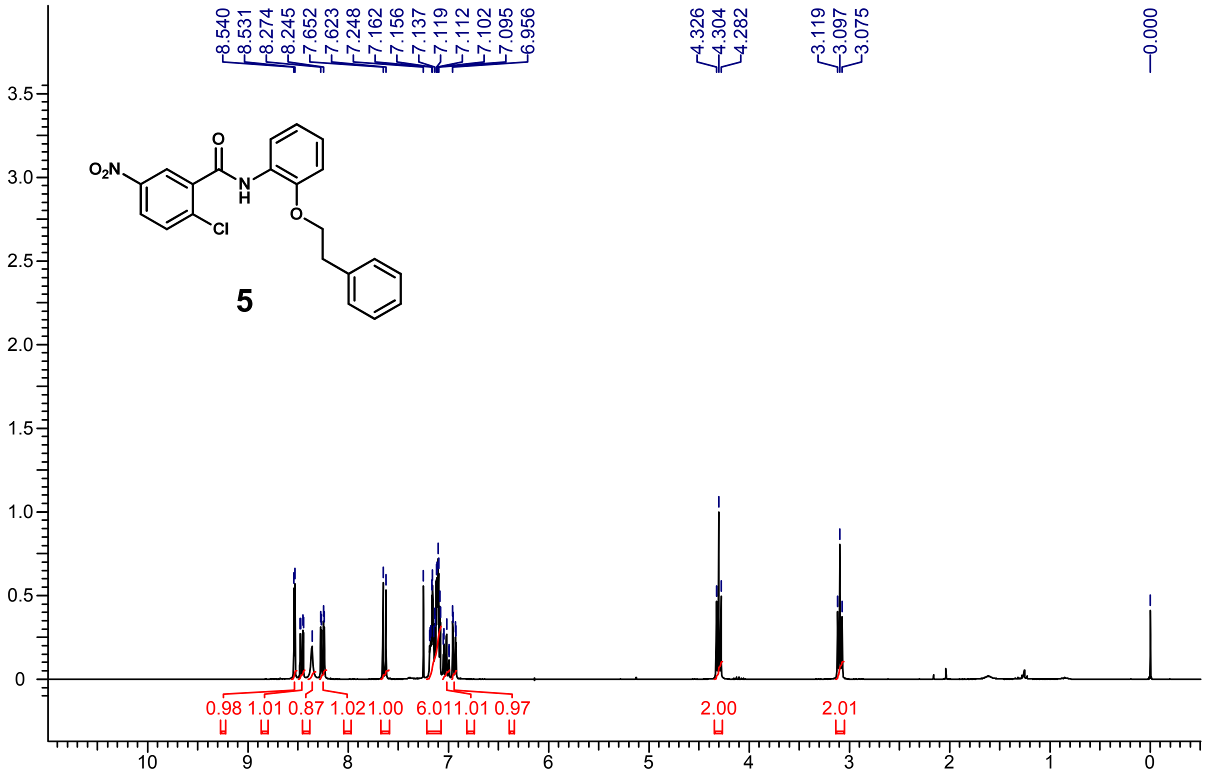
8.540
8.531
8.274
8.245
7.652
7.623
7.248
7.162
7.156
7.137
7.119
7.112
7.102
7.095
6.956
4.326
4.304
4.282
3.119
3.097
3.075
0.000
O
O_2N
N
H
Cl
O
5
0.98 1.01 0.87 1.02 1.00 6.01 1.01 0.97
2.00
2.01

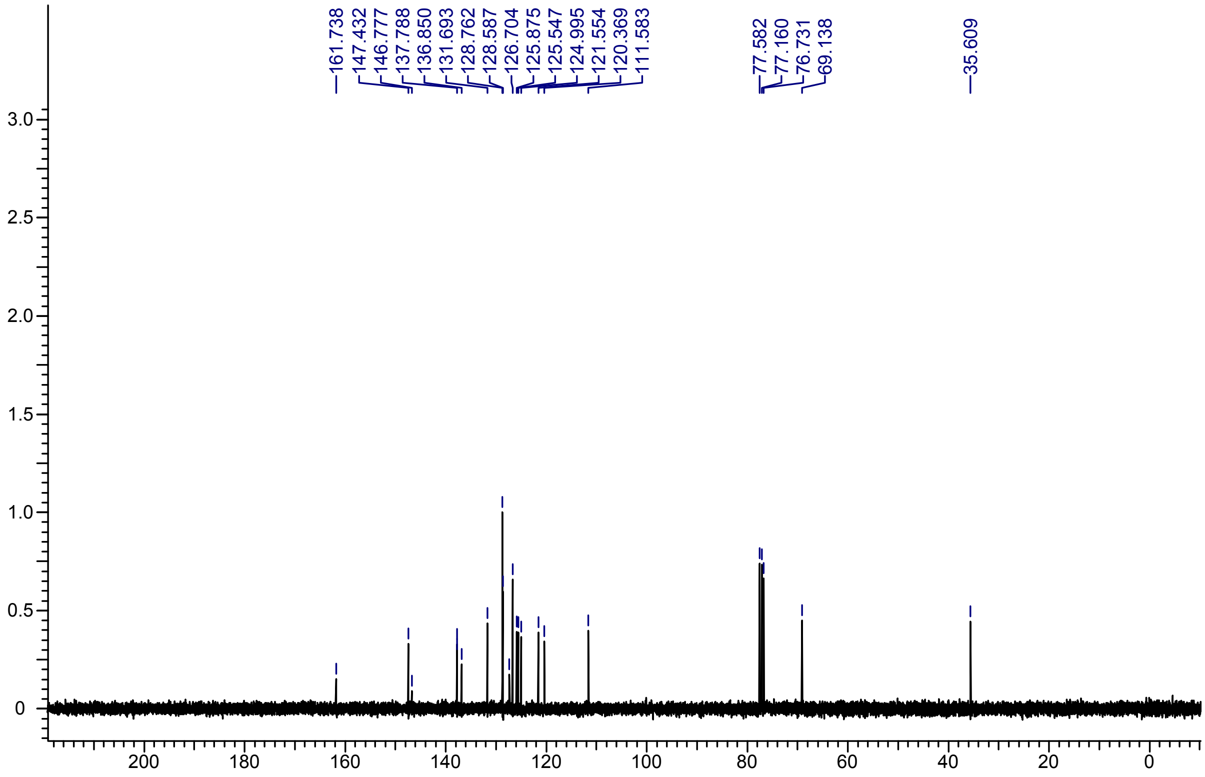
161.738
147.432
146.777
137.788
136.850
131.693
128.762
128.587
126.704
125.875
125.547
124.995
121.554
120.369
111.583
77.582
77.160
76.731
69.138
35.609

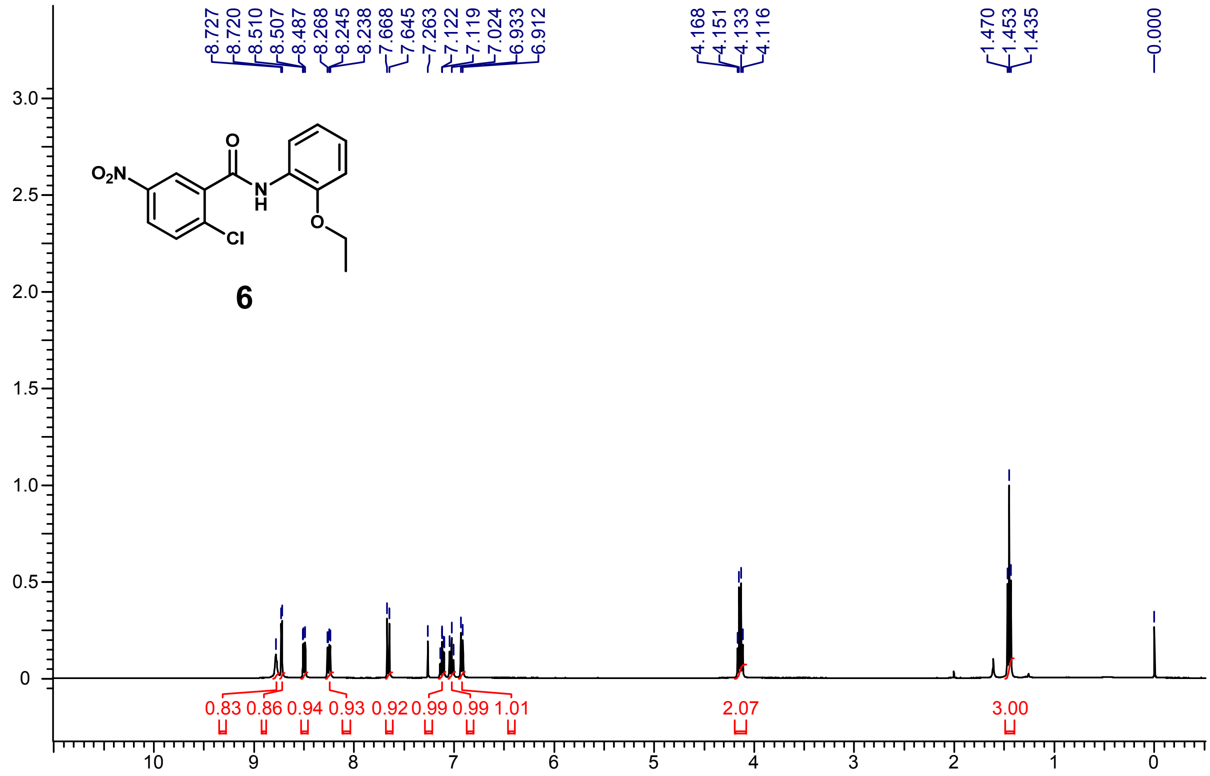
8.727 8.720 8.510 8.507 8.487 8.268 8.245 8.238 7.668 7.645 7.263 7.122 7.119 7.024 6.933 6.912
4.168 4.151 4.133 4.116
1.470 1.453 1.435
0.000
O_2N
O
N
H
Cl
O
6
0.83 0.86 0.94 0.93 0.92 0.99 0.99 1.01
2.07
3.00

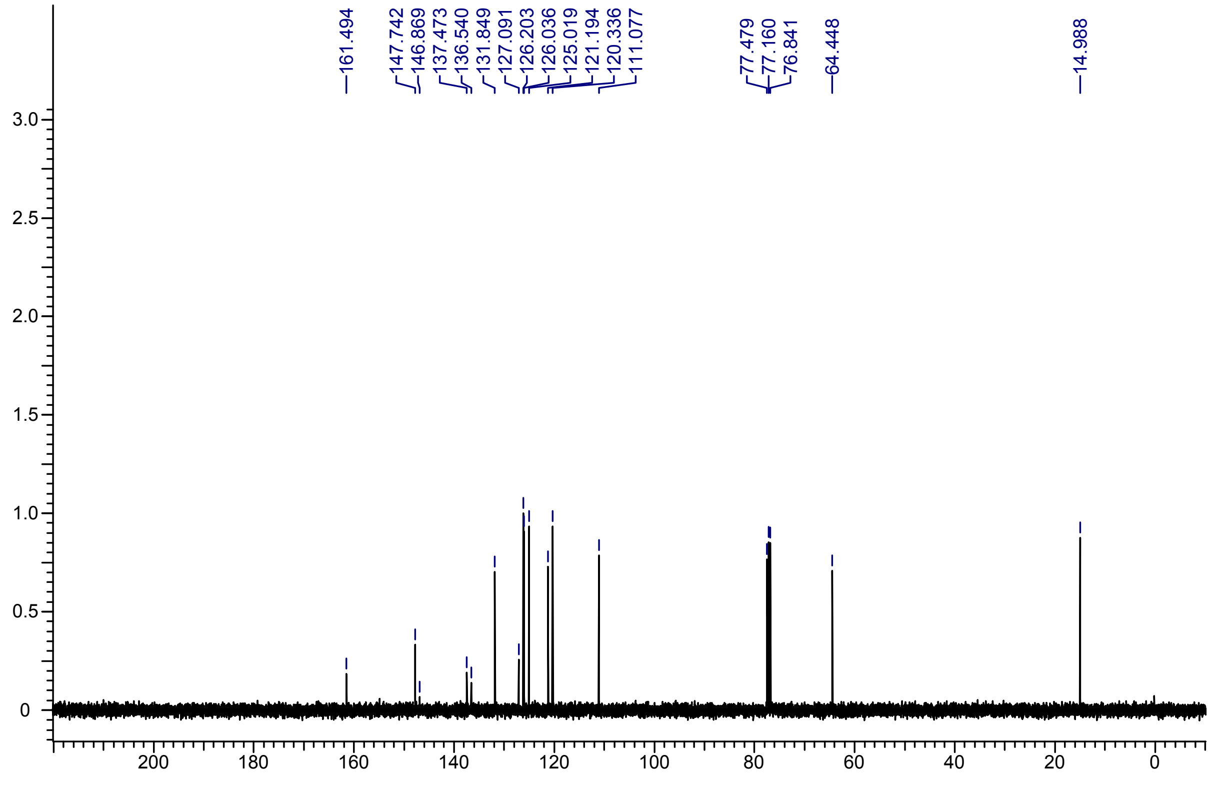
161.494
147.742 146.869 137.473 136.540 131.849 127.091 126.203 126.036 125.019 121.194 120.336 111.077
77.479 77.160 76.841
64.448
14.988

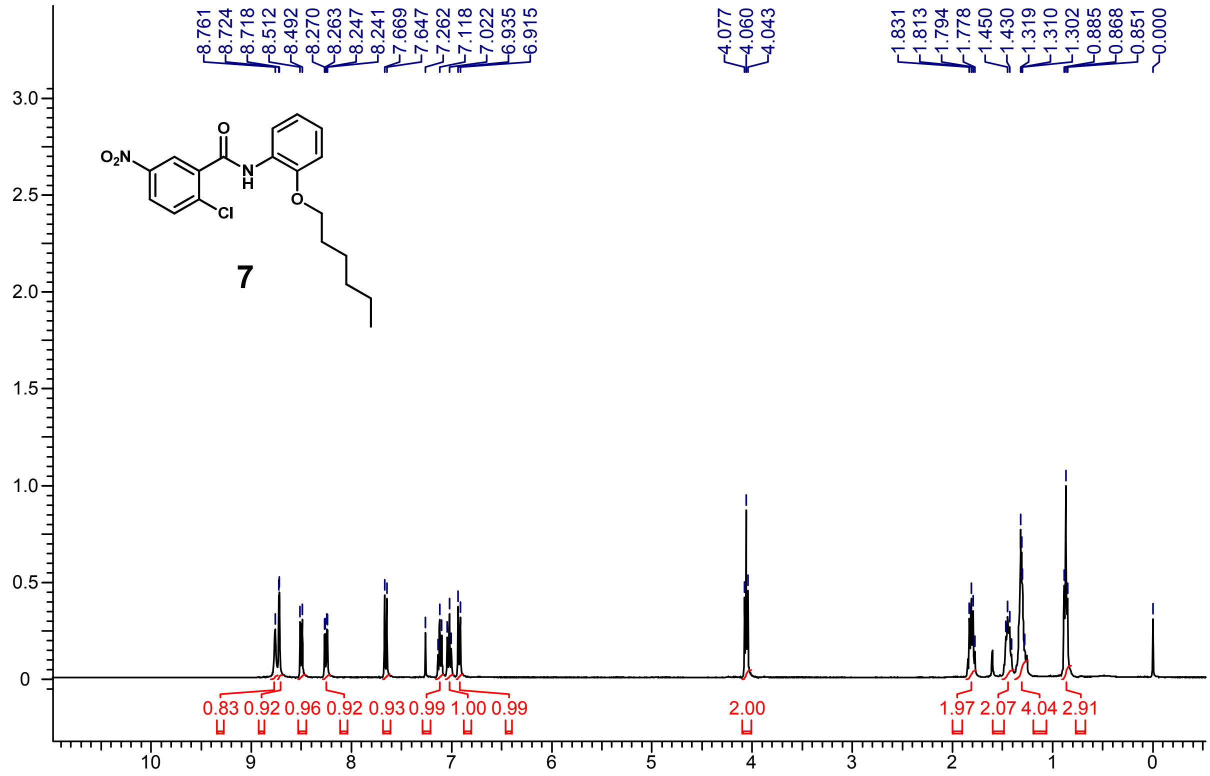
8.761
8.724
8.718
8.512
8.492
8.270
8.263
8.247
8.241
7.669
7.647
7.262
7.118
7.022
6.935
6.915
4.077
4.060
4.043
1.831
1.813
1.794
1.778
1.450
1.430
1.319
1.310
1.302
0.885
0.868
0.851
0.000
O2N
O
N
H
Cl
O
7
0.83
0.92
0.96
0.92
0.93
0.99
1.00
0.99
2.00
1.97
2.07
4.04
2.91

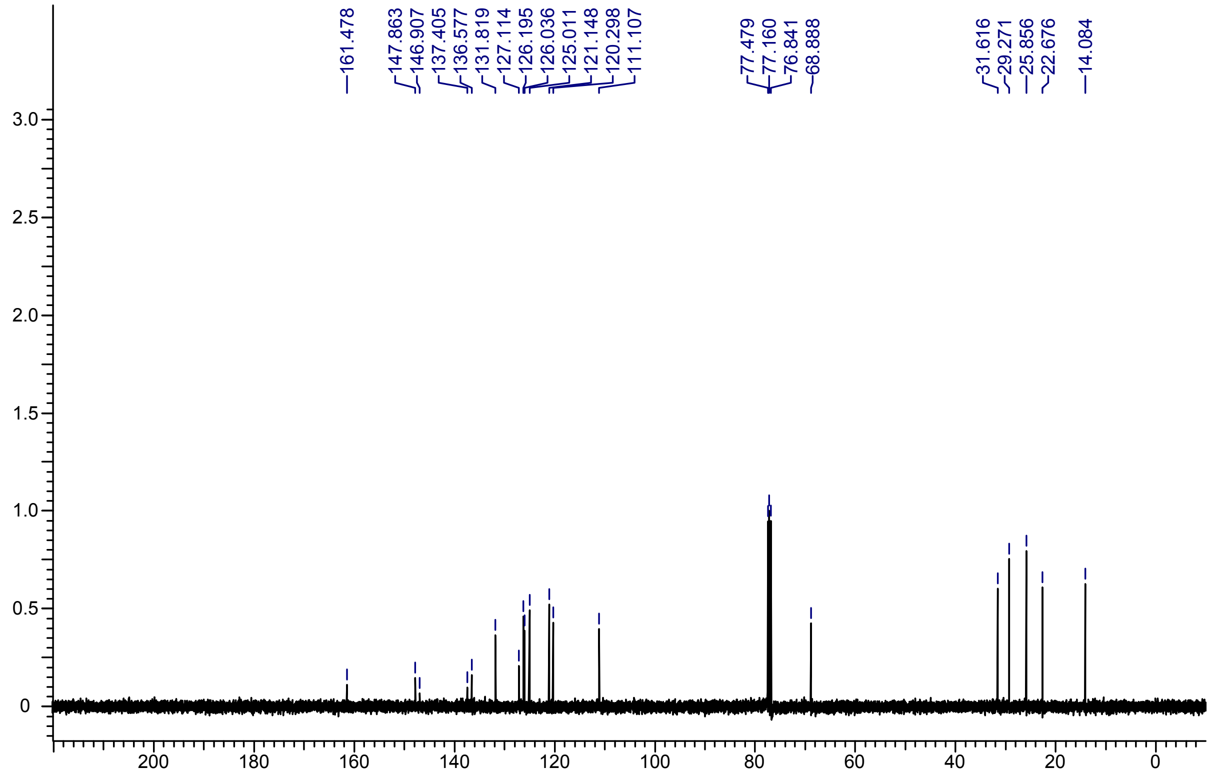
161.478
147.863
146.907
137.405
136.577
131.819
127.114
126.195
126.036
125.011
121.148
120.298
111.107
77.479
77.160
76.841
68.888
31.616
29.271
25.856
22.676
14.084

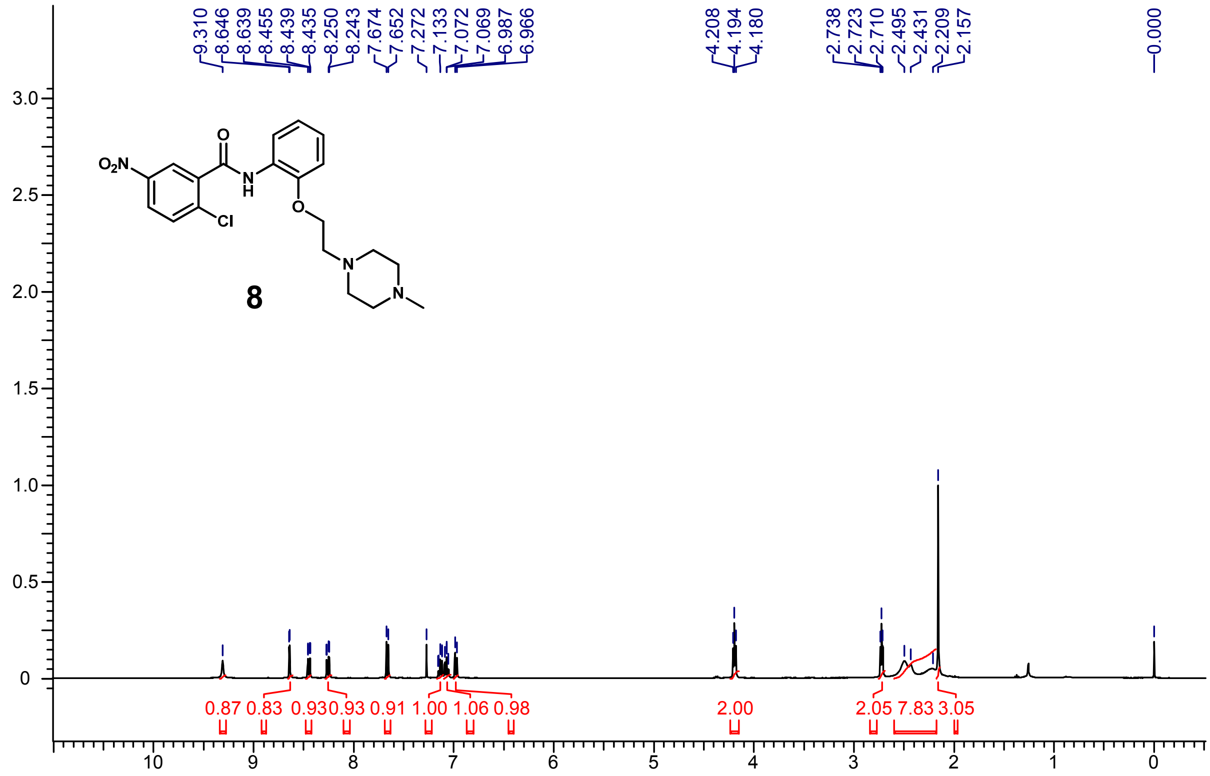

9.310
8.646
8.639
8.455
8.439
8.435
8.250
8.243
7.674
7.652
7.272
7.133
7.072
7.069
6.987
6.966
4.208
4.194
4.180
2.738
2.723
2.710
2.495
2.431
2.209
2.157
0.000
0.87 0.83 0.93 0.93 0.91 1.00 1.06 0.98
2.00
2.05 7.83 3.05

8

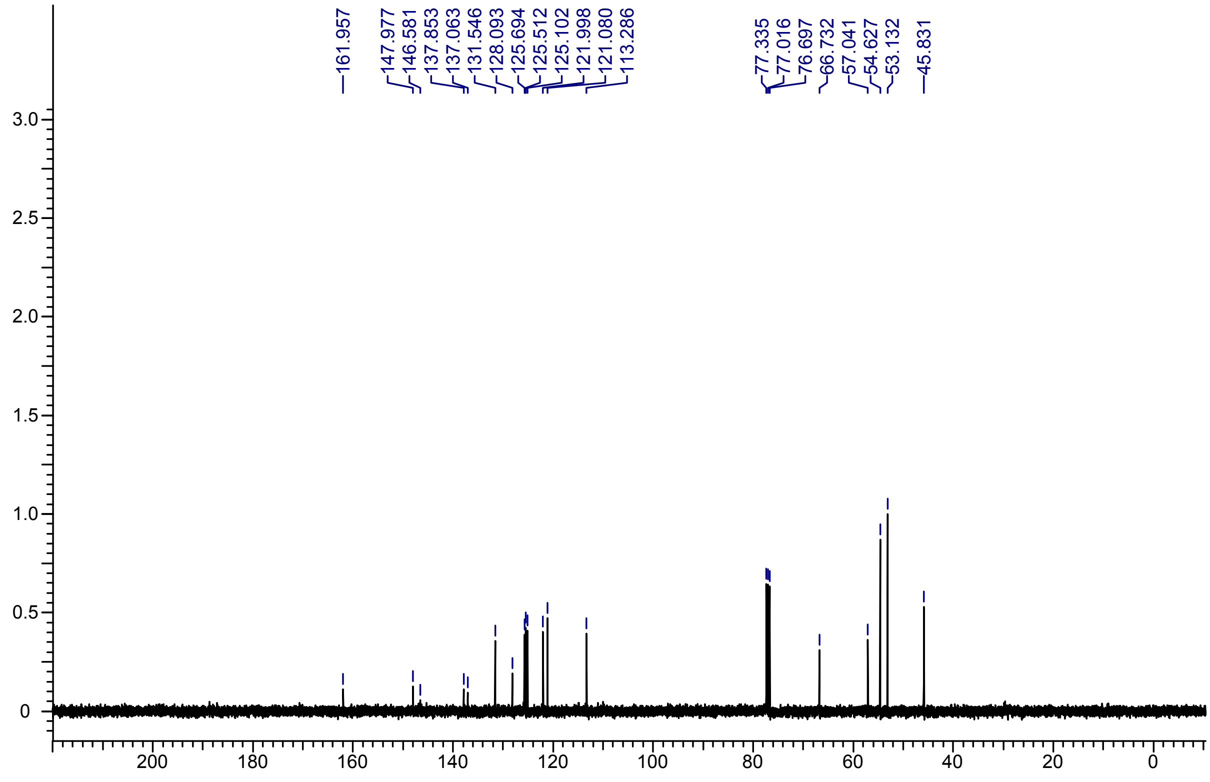

161.957
147.977
146.581
137.853
137.063
131.546
128.093
125.694
125.512
125.102
121.998
121.080
113.286
77.335
77.016
76.697
66.732
57.041
54.627
53.132
45.831

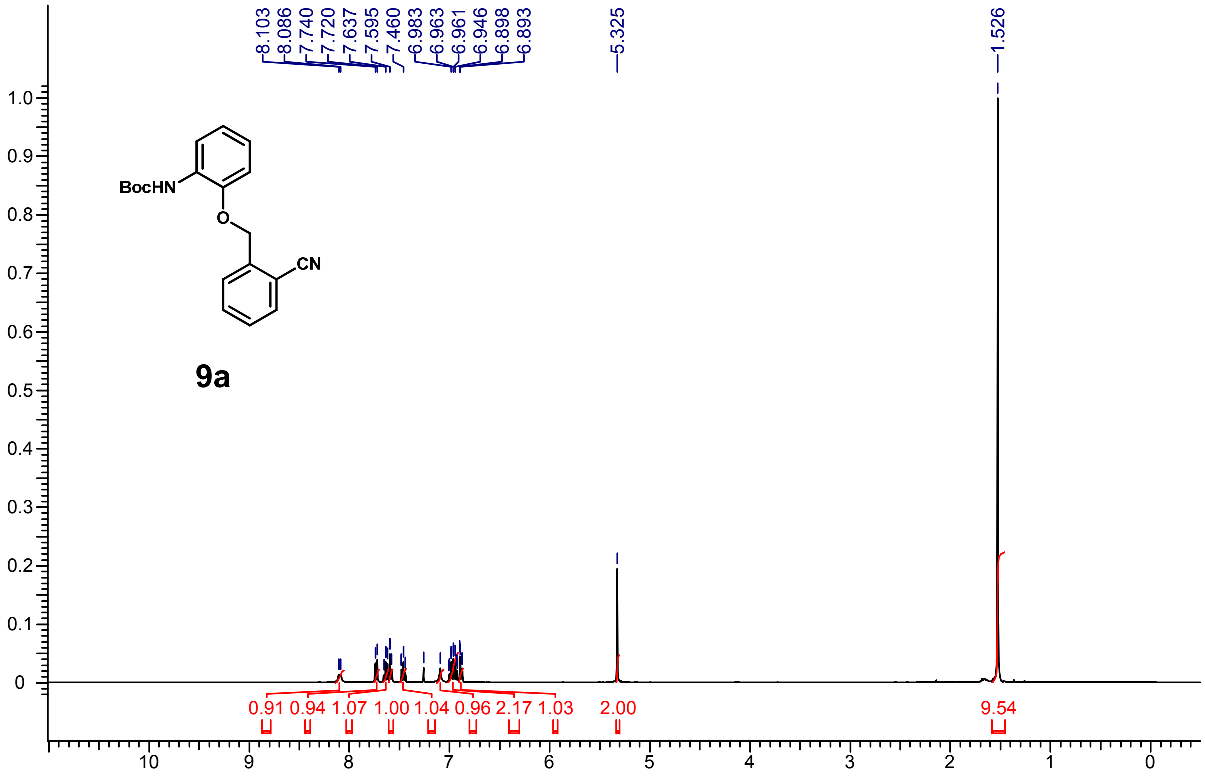
8.103
8.086
7.740
7.720
7.637
7.595
7.460
6.983
6.963
6.961
6.946
6.898
6.893
5.325
1.526
BocHN
O
CN
9a
0.91 0.94 1.07 1.00 1.04 0.96 2.17 1.03
2.00
9.54

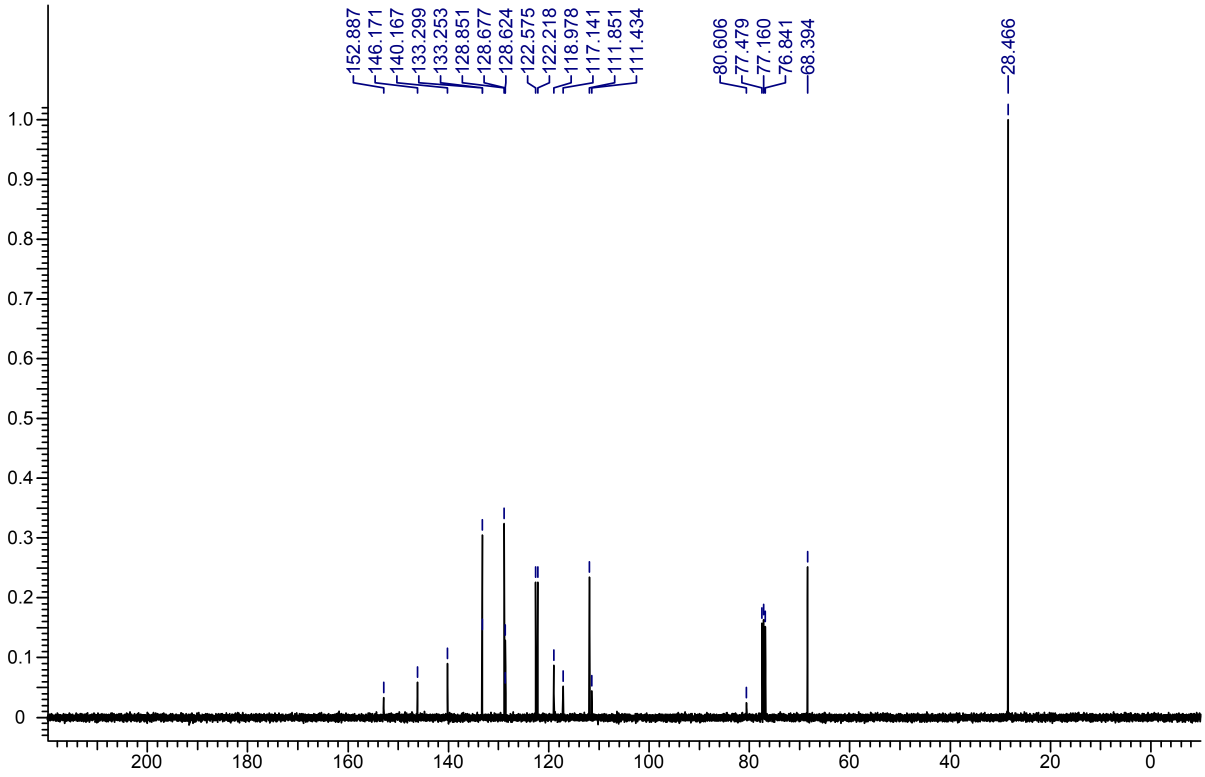
152.887
146.171
140.167
133.299
133.253
128.851
128.677
128.624
122.575
122.218
118.978
117.141
111.851
111.434
80.606
77.479
77.160
76.841
68.394
28.466

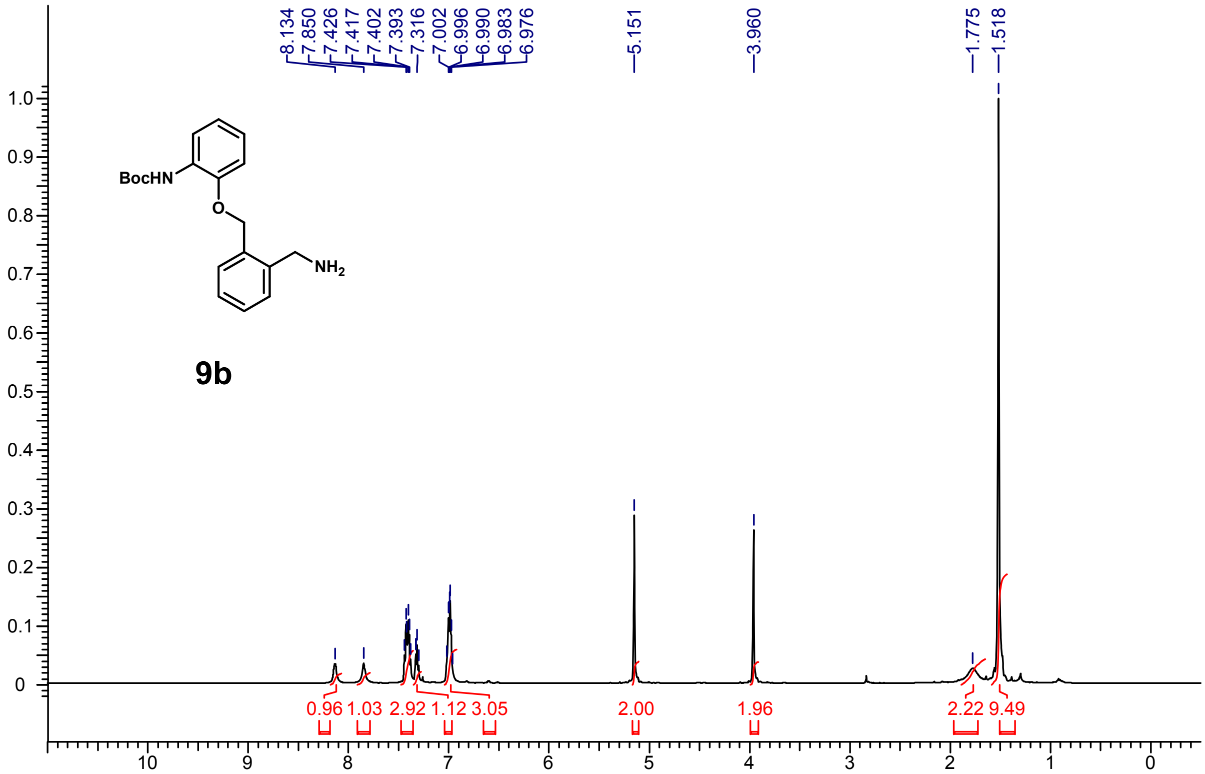
8.134
7.850
7.426
7.417
7.402
7.393
7.316
7.002
6.996
6.990
6.983
6.976
5.151
3.960
1.775
1.518
BocHN
O
NH2
9b
0.96 1.03 2.92 1.12 3.05
2.00
1.96
2.22 9.49

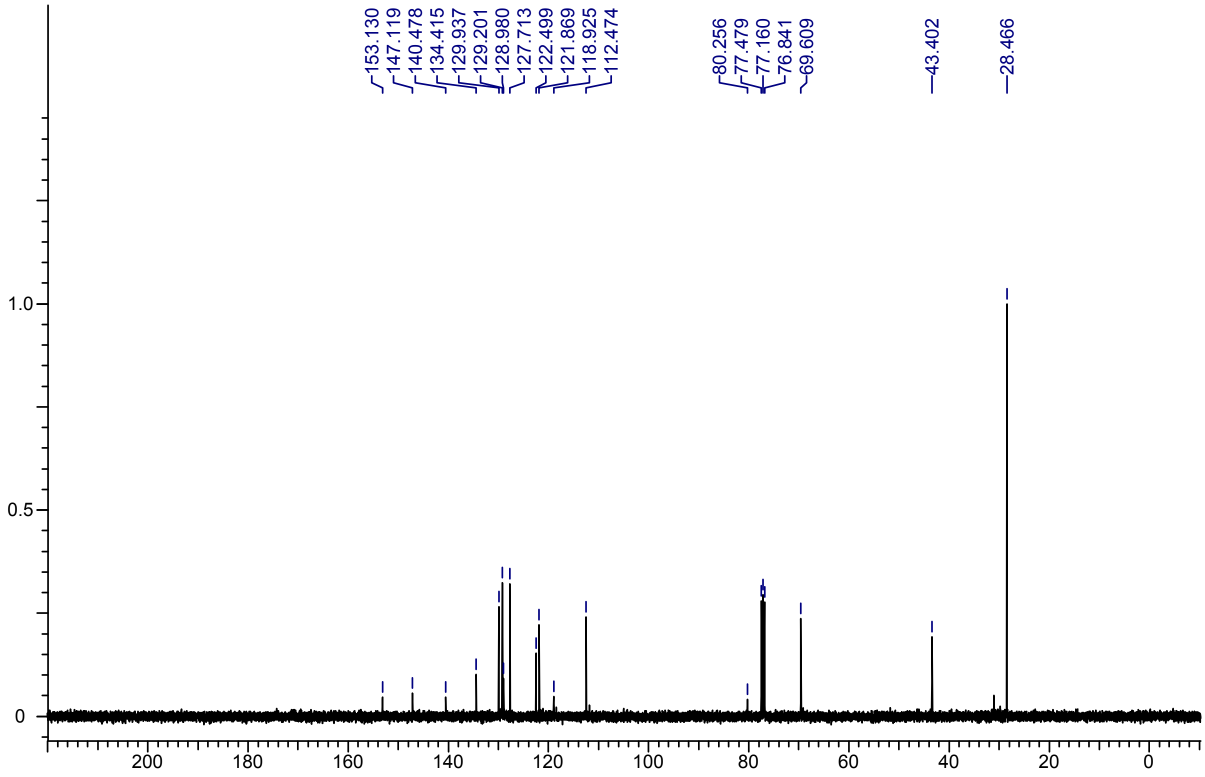
153.130
147.119
140.478
134.415
129.937
129.201
128.980
127.713
122.499
121.869
118.925
112.474
80.256
77.479
77.160
76.841
69.609
43.402
28.466

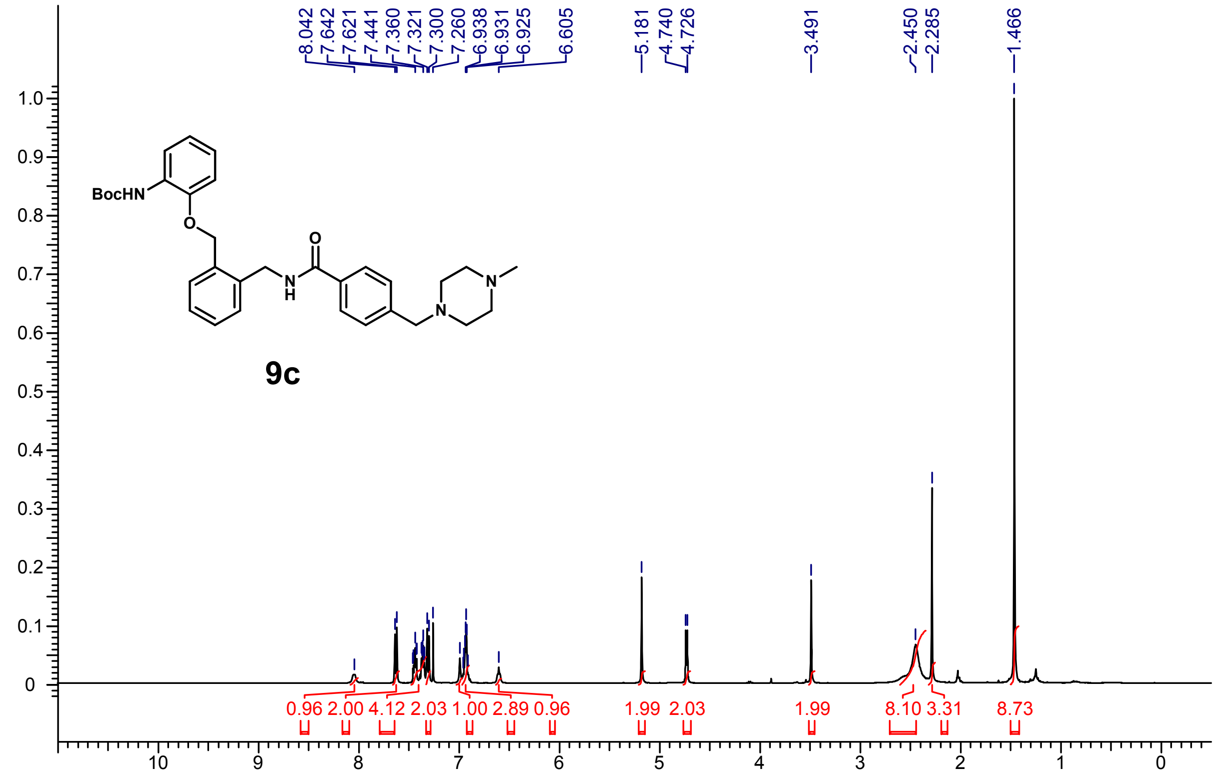
8.042
7.642
7.621
7.441
7.360
7.321
7.300
7.260
6.938
6.931
6.925
6.605
5.181
4.740
4.726
3.491
2.450
2.285
1.466
BocHN
O
N
H
O
N
N
9c
0.96 2.00 4.12 2.03 1.00 2.89 0.96
1.99 2.03
1.99
8.10 3.31
8.73

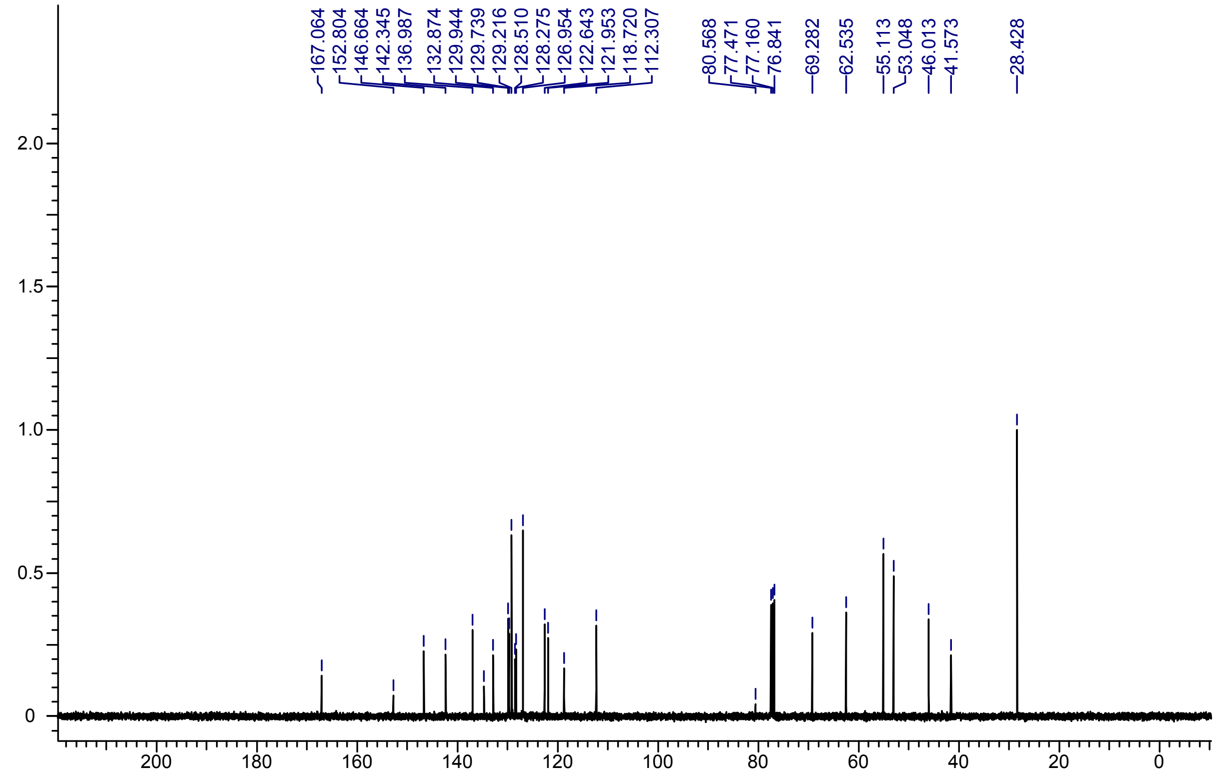
167.064
152.804
146.664
142.345
136.987
132.874
129.944
129.739
129.216
128.510
128.275
126.954
122.643
121.953
118.720
112.307
80.568
77.471
77.160
76.841
69.282
62.535
55.113
53.048
46.013
41.573
28.428

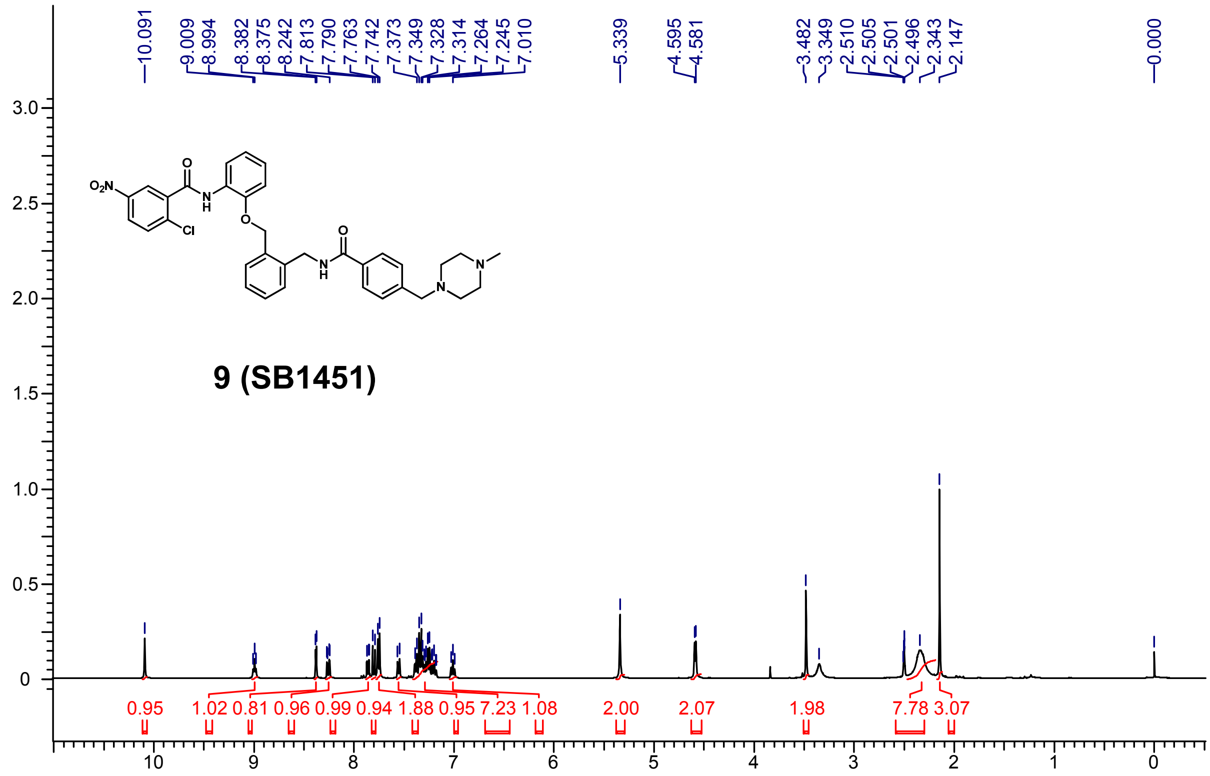
10.091
9.009
8.994
8.382
8.375
8.242
7.813
7.790
7.763
7.742
7.373
7.349
7.328
7.314
7.264
7.245
7.010
5.339
4.595
4.581
3.482
3.349
2.510
2.505
2.501
2.496
2.343
2.147
0.000
O_2N
O
N
H
Cl
9 (SB1451)
0.95 1.02 0.81 0.96 0.99 0.94 1.88 0.95 7.23 1.08 2.00 2.07 1.98 7.78 3.07

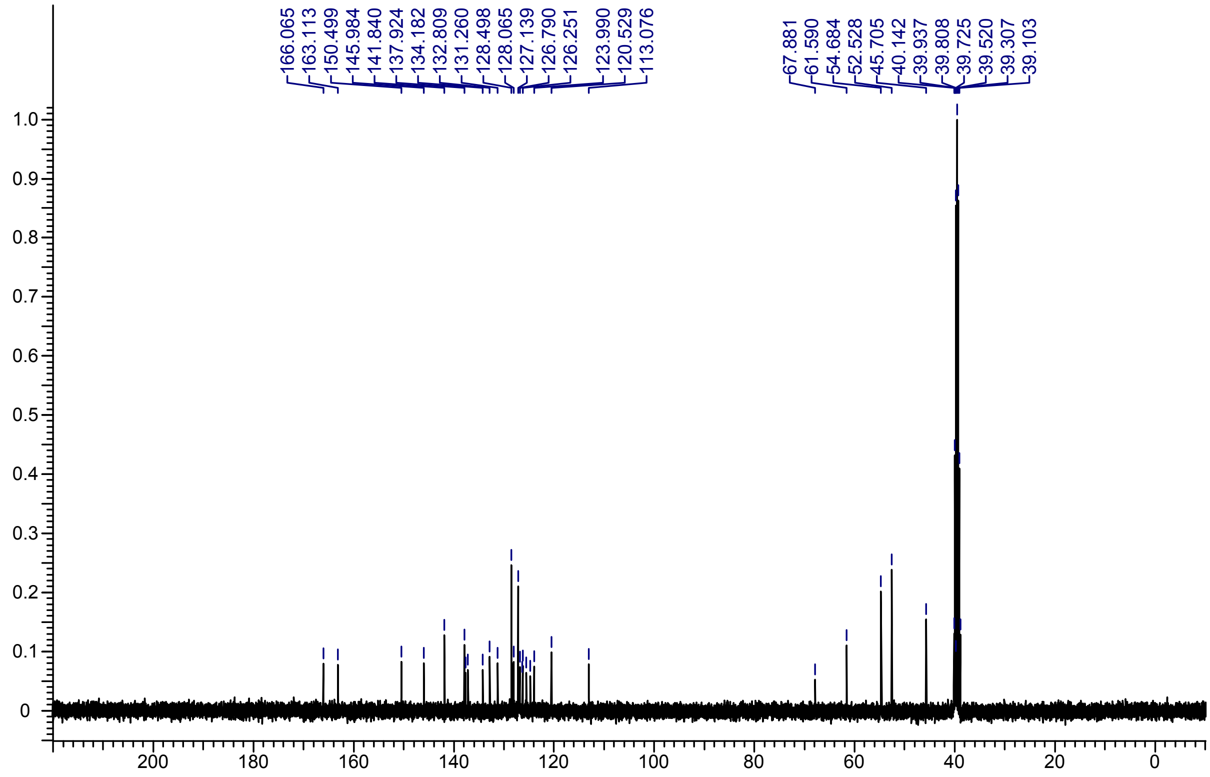
166.065
163.113
150.499
145.984
141.840
137.924
134.182
132.809
131.260
128.498
128.065
127.139
126.790
126.251
123.990
120.529
113.076
67.881
61.590
54.684
52.528
45.705
40.142
39.937
39.808
39.725
39.520
39.307
39.103

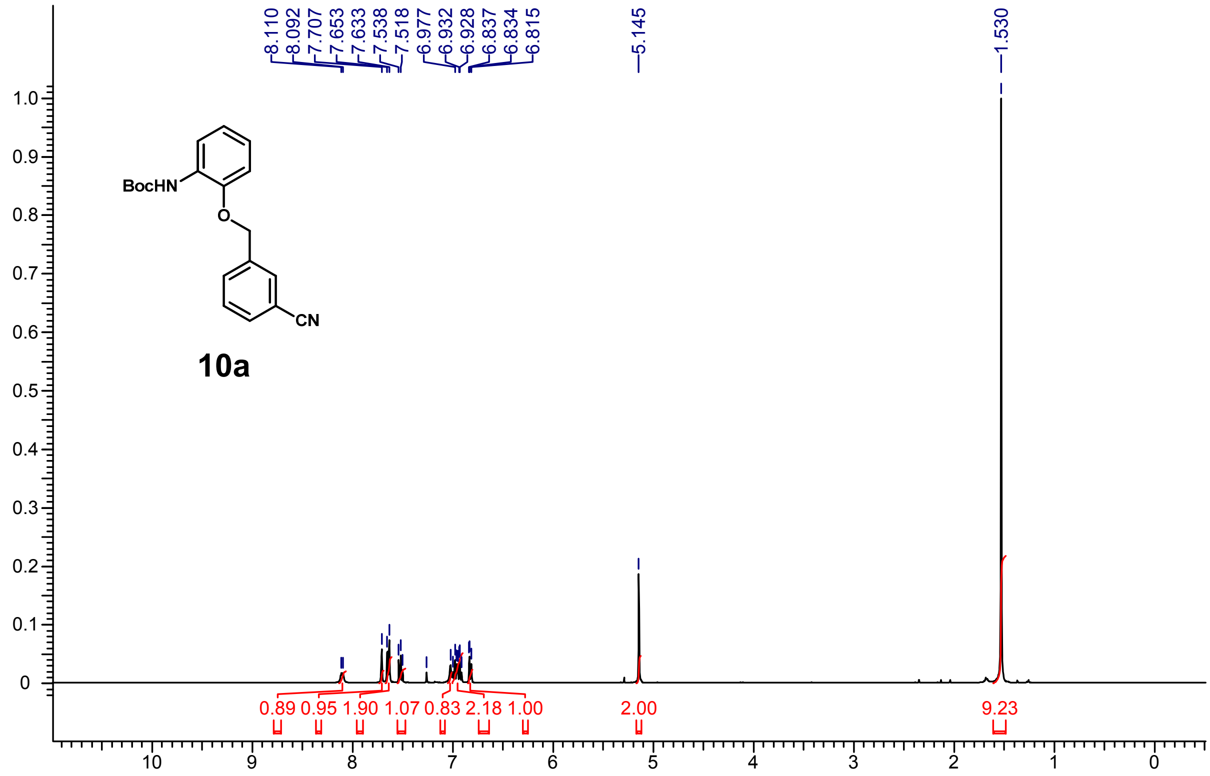
8.110
8.092
7.707
7.653
7.633
7.538
7.518
6.977
6.932
6.928
6.837
6.834
6.815
5.145
1.530
BocHN
O
CN
10a
0.89
0.95
1.90
1.07
0.83
2.18
1.00
2.00
9.23

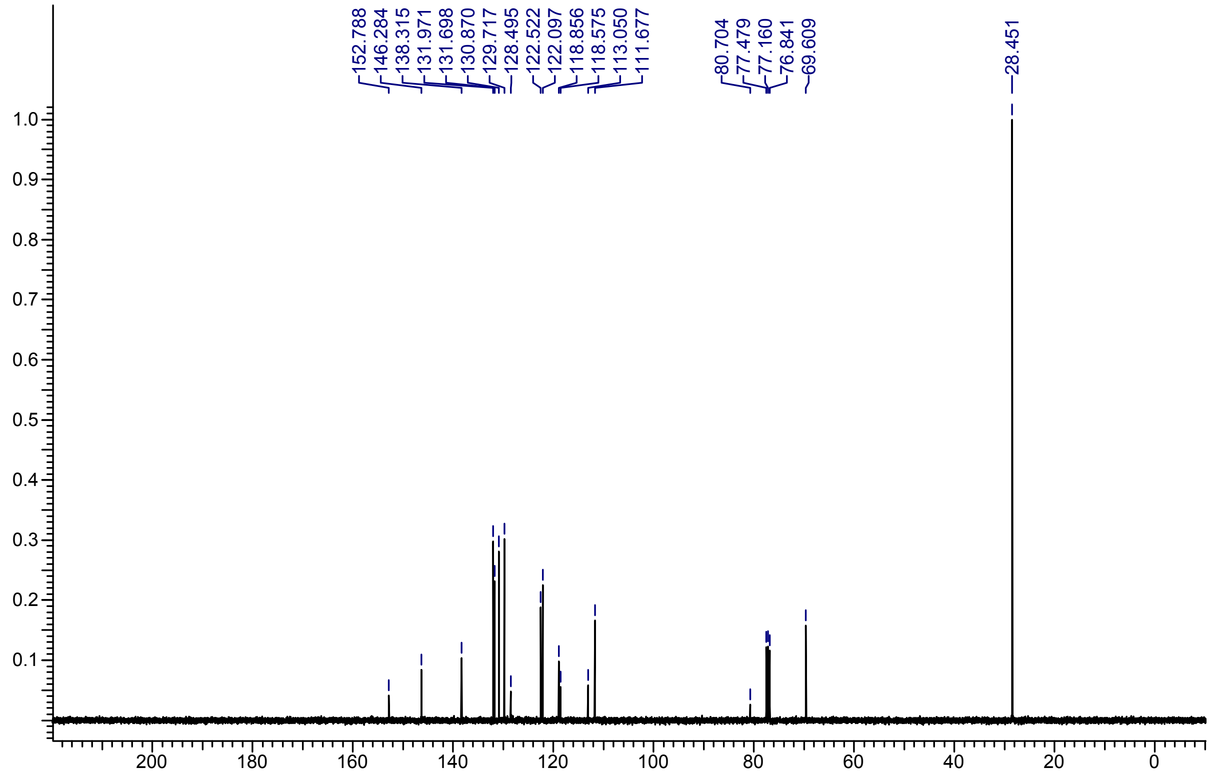
152.788
146.284
138.315
131.971
131.698
130.870
129.717
128.495
122.522
122.097
118.856
118.575
113.050
111.677
80.704
77.479
77.160
76.841
69.609
28.451

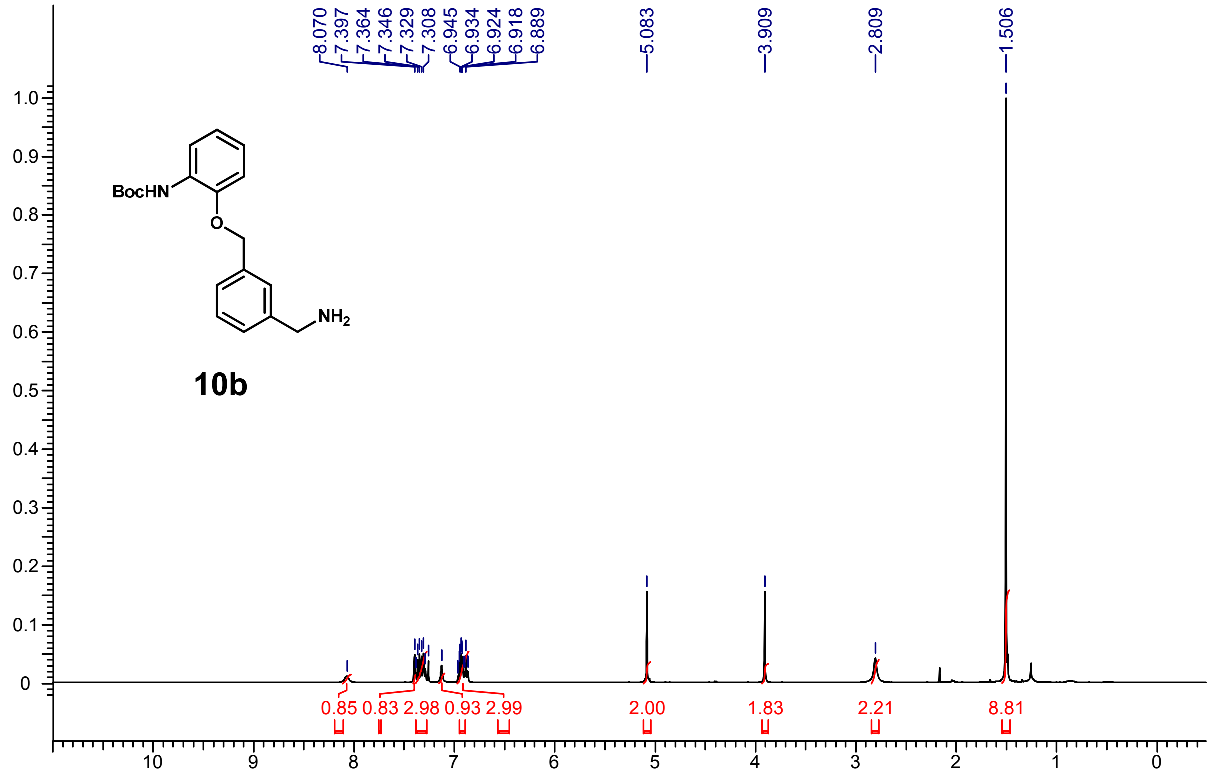
8.070
7.397
7.364
7.346
7.329
7.308
6.945
6.934
6.924
6.918
6.889
5.083
3.909
2.809
1.506
BocHN
O
NH2
10b
0.85 0.83 2.98 0.93 2.99
2.00
1.83
2.21
8.81

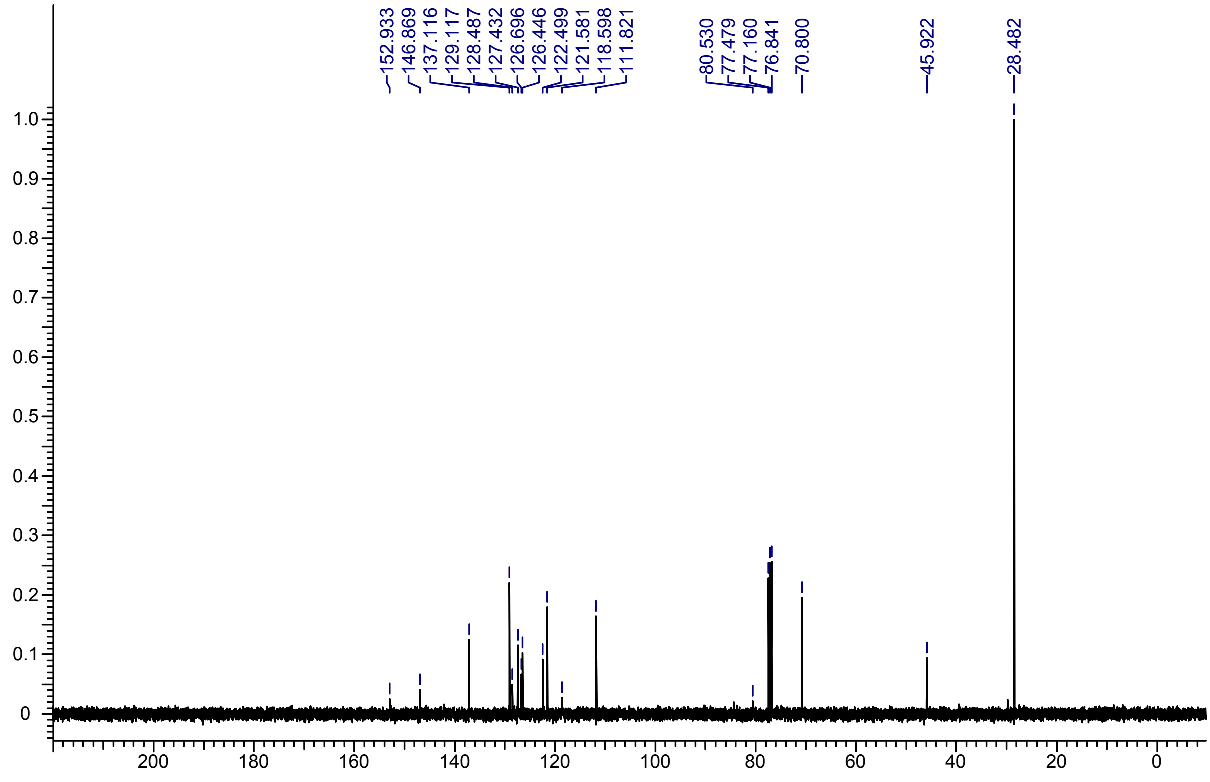
152.933
146.869
137.116
129.117
128.487
127.432
126.696
126.446
122.499
121.581
118.598
111.821
80.530
77.479
77.160
76.841
70.800
45.922
28.482

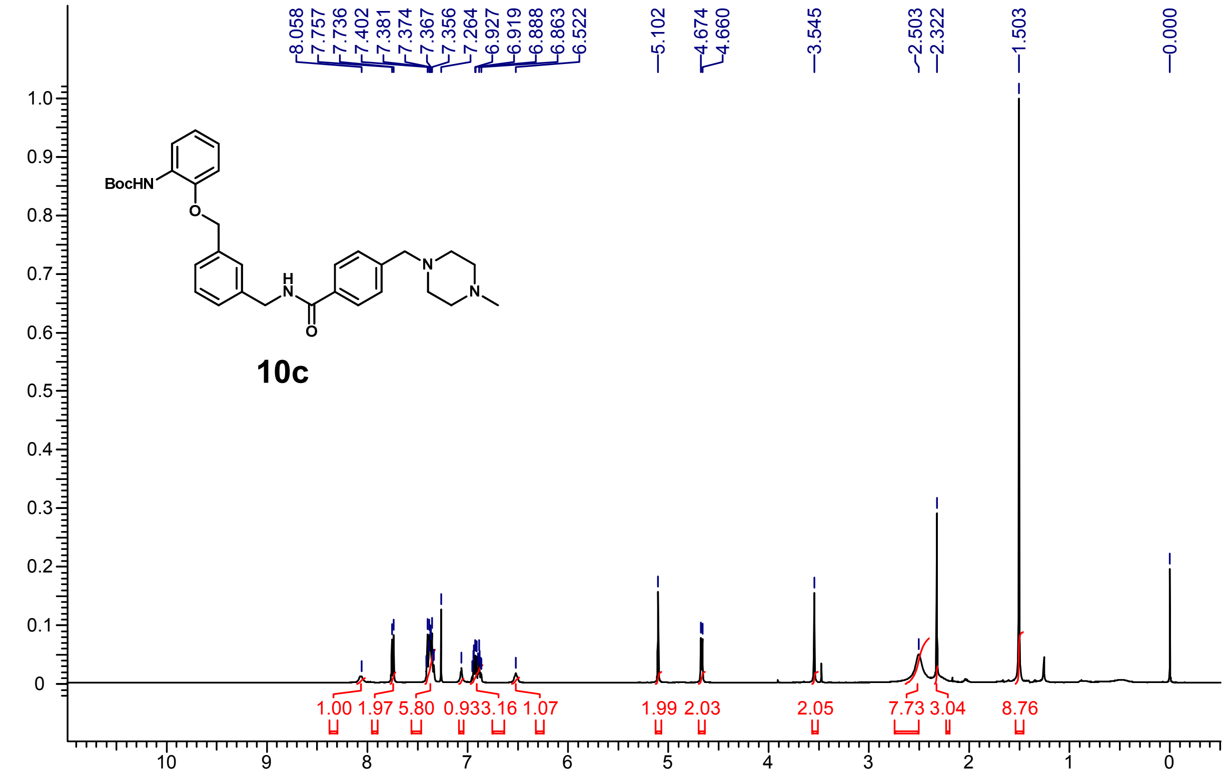
8.058 7.757 7.736 7.402 7.381 7.374 7.367 7.356 7.264 6.927 6.919 6.888 6.863 6.522
5.102
4.674 4.660
3.545
2.503 2.322
1.503
0.000
BocHN
O
H
N
O
N
N
10c
1.00 1.97 5.80 0.93 3.16 1.07 1.99 2.03 2.05 7.73 3.04 8.76

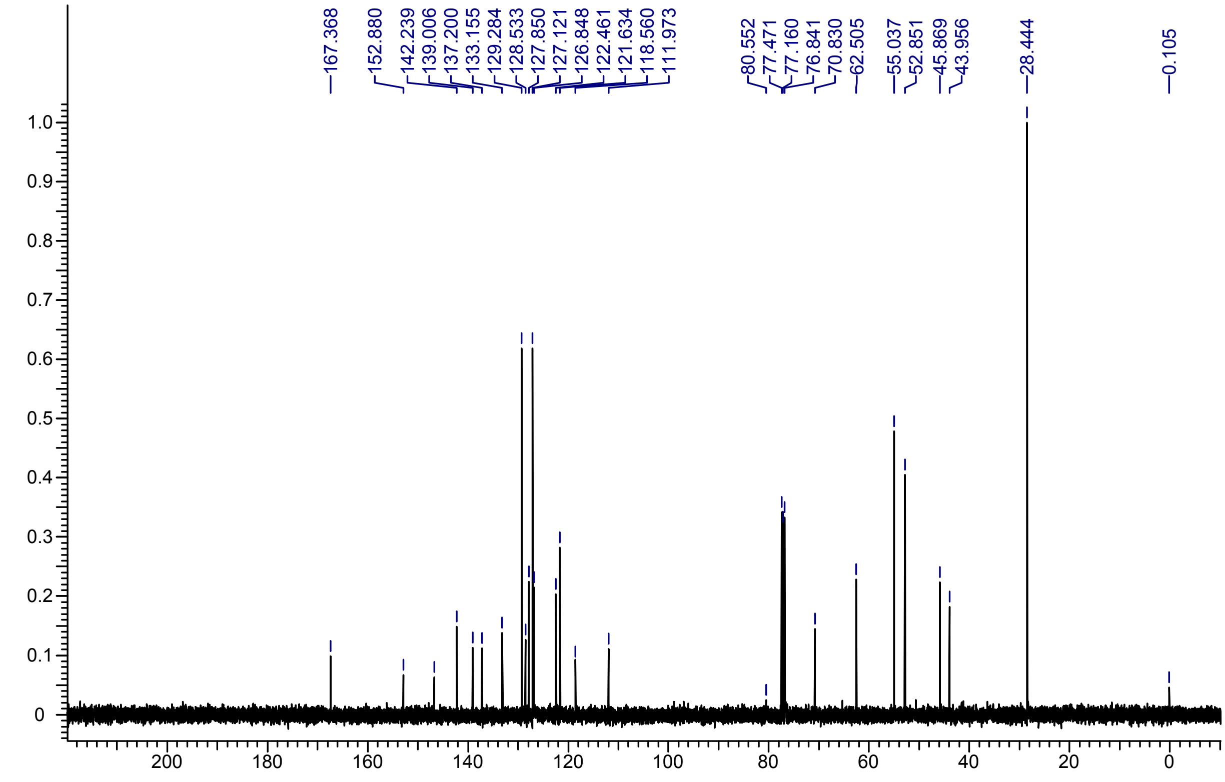
167.368
152.880 142.239 139.006 137.200 133.155 129.284 128.533 127.850 127.121 126.848 122.461 121.634 118.560 111.973
80.552 77.471 77.160 76.841 70.830 62.505
55.037 52.851 45.869 43.956
28.444
0.105

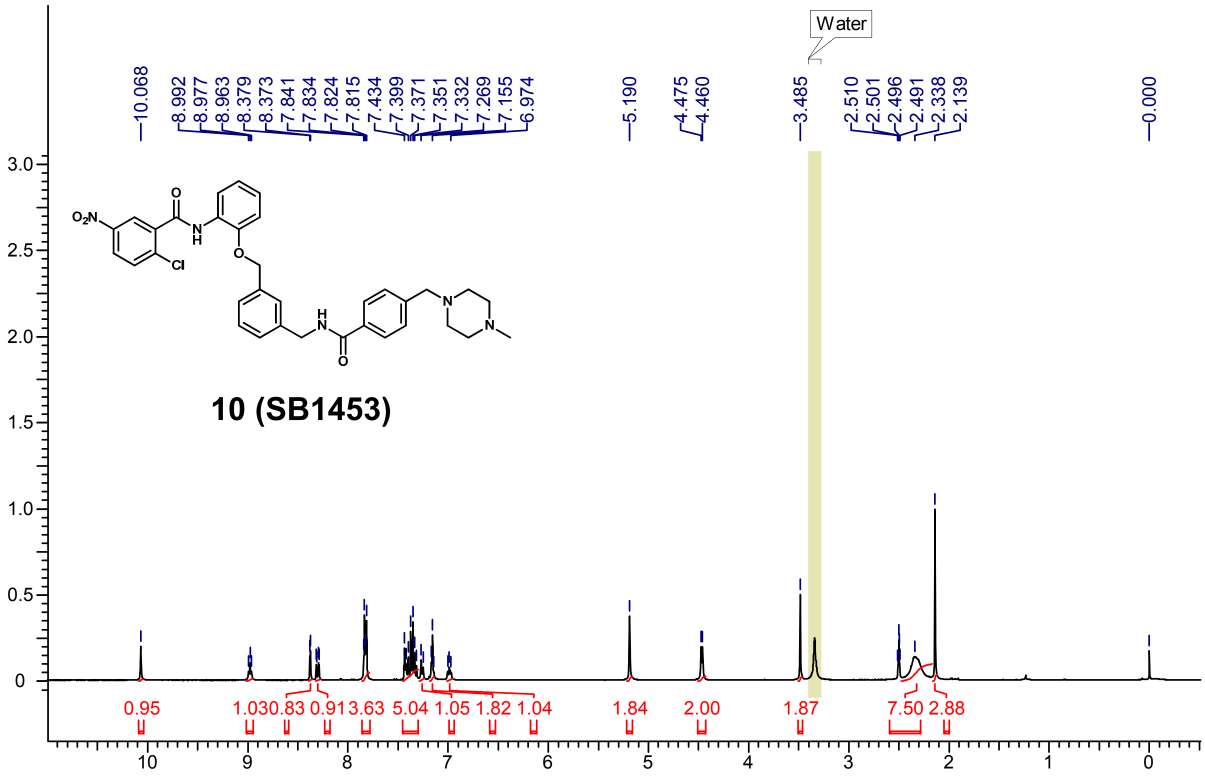
Water
10.068
8.992
8.977
8.963
8.379
8.373
7.841
7.834
7.824
7.815
7.434
7.399
7.371
7.351
7.332
7.269
7.155
6.974
5.190
4.475
4.460
3.485
2.510
2.501
2.496
2.491
2.338
2.139
0.000
0.95
1.03
0.83
0.91
3.63
5.04
1.05
1.82
1.04
1.84
2.00
1.87
7.50
2.88

10 (SB1453)

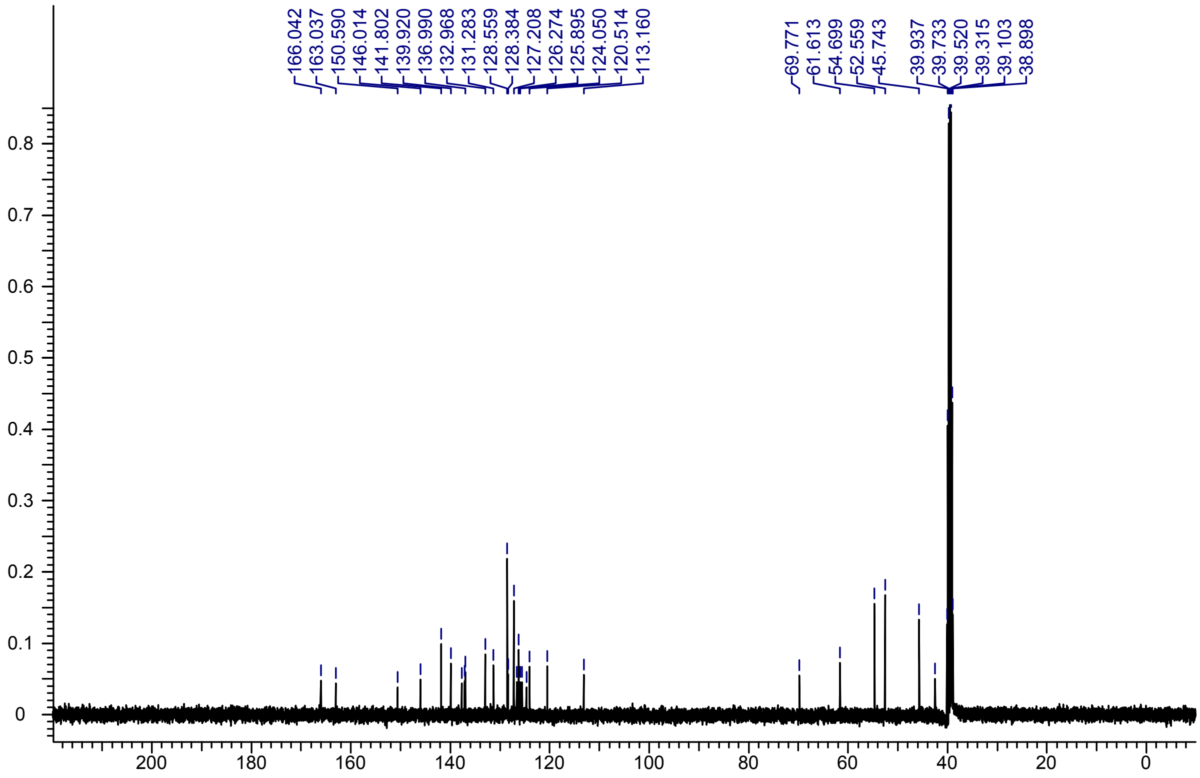
166.042
163.037
150.590
146.014
141.802
139.920
136.990
132.968
131.283
128.559
128.384
127.208
126.274
125.895
124.050
120.514
113.160
69.771
61.613
54.699
52.559
45.743
39.937
39.733
39.520
39.315
39.103
38.898

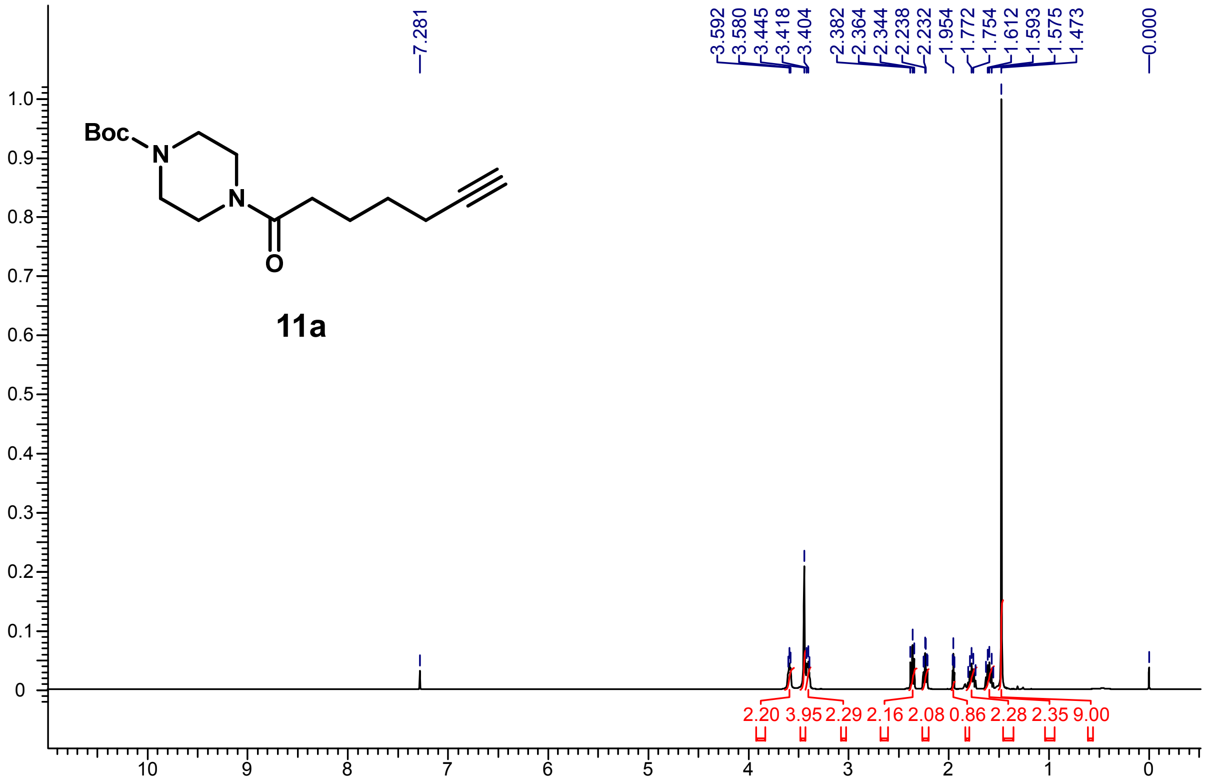

7.281
3.592
3.580
3.445
3.418
3.404
2.382
2.364
2.344
2.238
2.232
1.954
1.772
1.754
1.612
1.593
1.575
1.473
0.000
Boc
N
N
O
11a
2.20
3.95
2.29
2.16
2.08
0.86
2.28
2.35
9.00

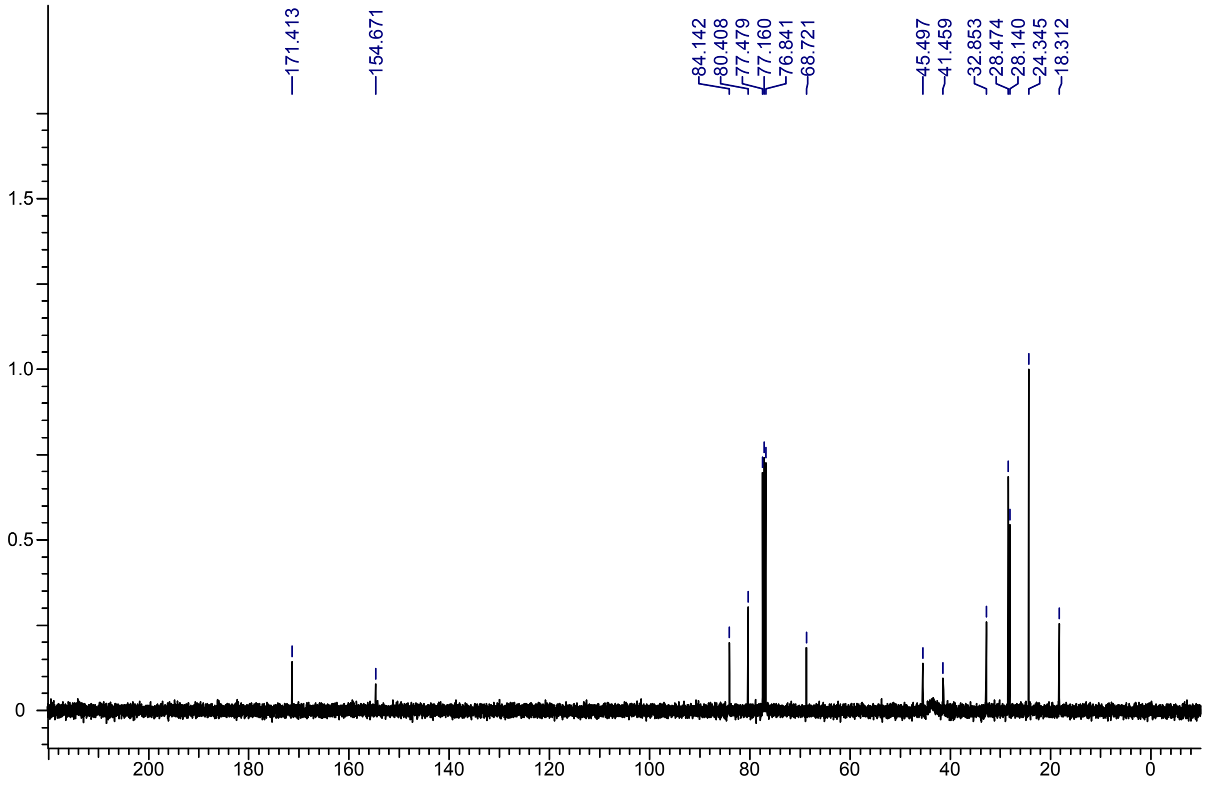

171.413
154.671
84.142
80.408
77.479
77.160
76.841
68.721
45.497
41.459
32.853
28.474
28.140
24.345
18.312

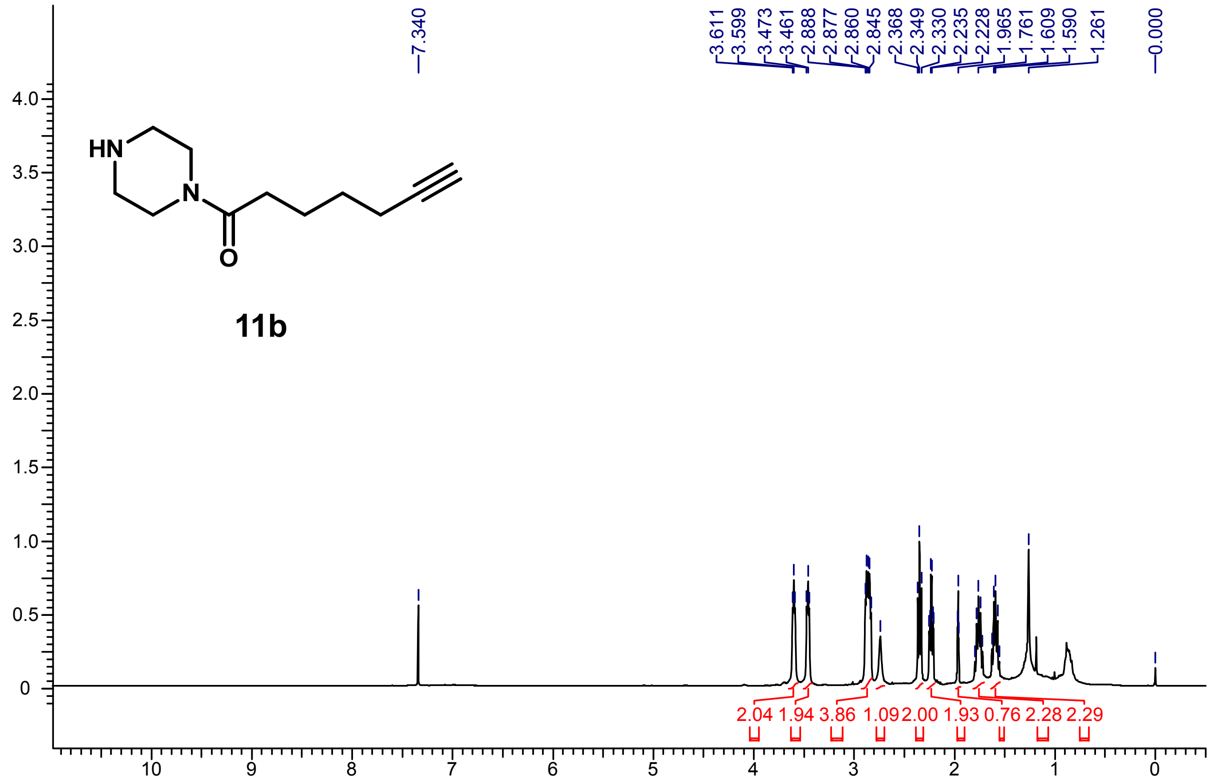

HN
N
O
11b
7.340
3.611
3.599
3.473
3.461
2.888
2.877
2.860
2.845
2.368
2.349
2.330
2.235
2.228
1.965
1.761
1.609
1.590
1.261
0.000
2.04 1.94 3.86 1.09 2.00 1.93 0.76 2.28 2.29

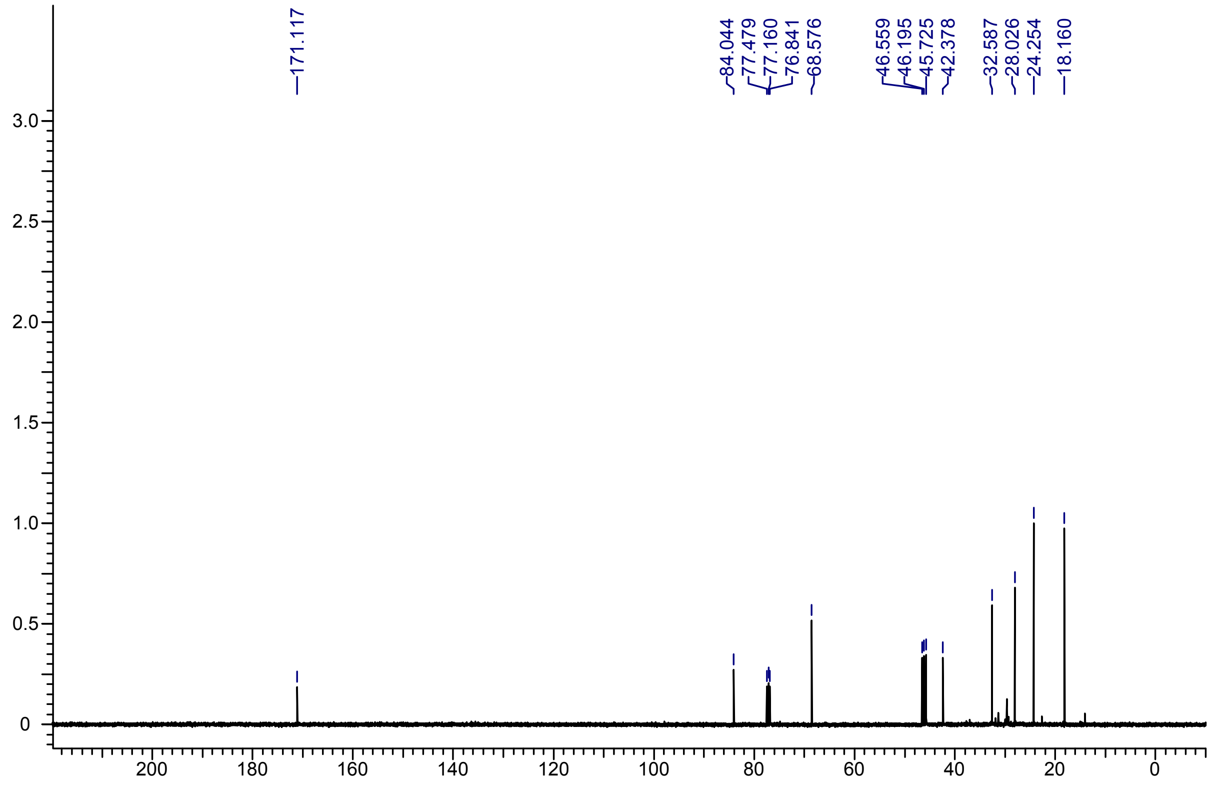

171.117
84.044
77.479
77.160
76.841
68.576
46.559
46.195
45.725
42.378
32.587
28.026
24.254
18.160

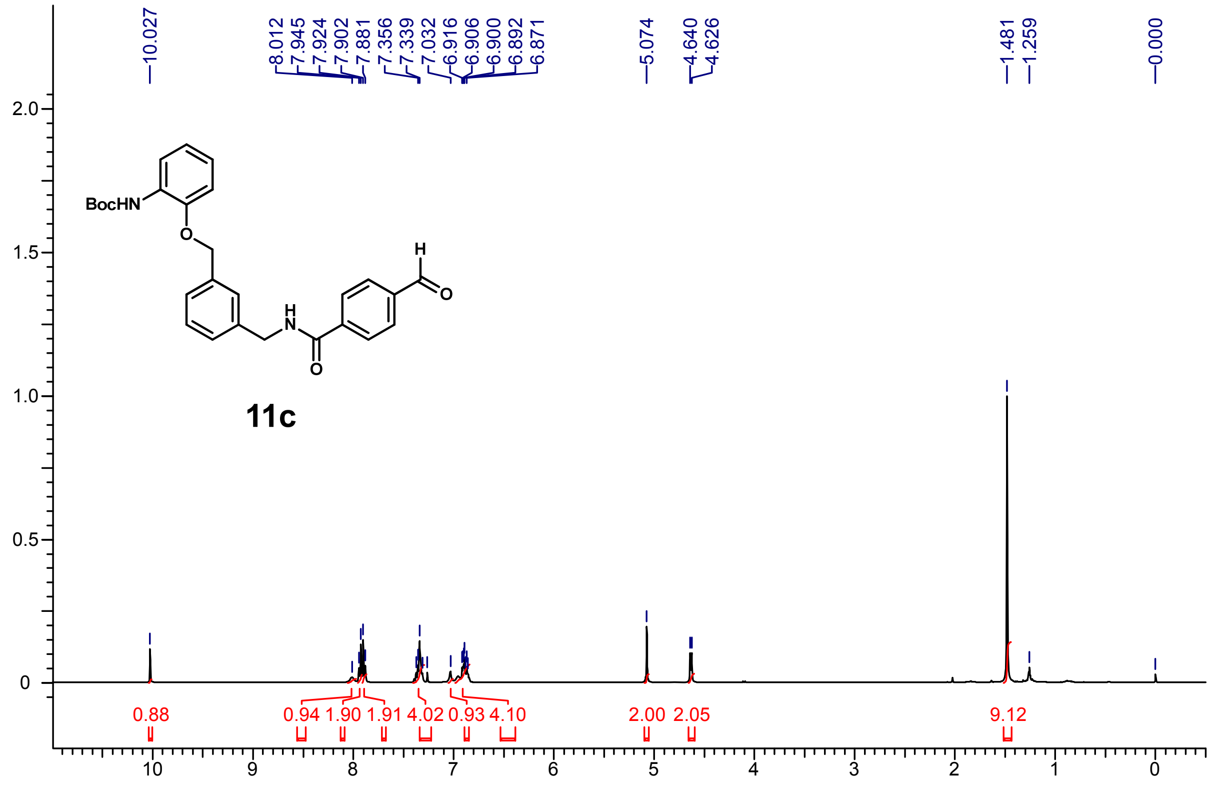
10.027
8.012
7.945
7.924
7.902
7.881
7.356
7.339
7.032
6.916
6.906
6.900
6.892
6.871
5.074
4.640
4.626
1.481
1.259
0.000
BocHN
O
H
N
H
O
O
11c
0.88
0.94 1.90 1.91 4.02 0.93 4.10
2.00 2.05
9.12

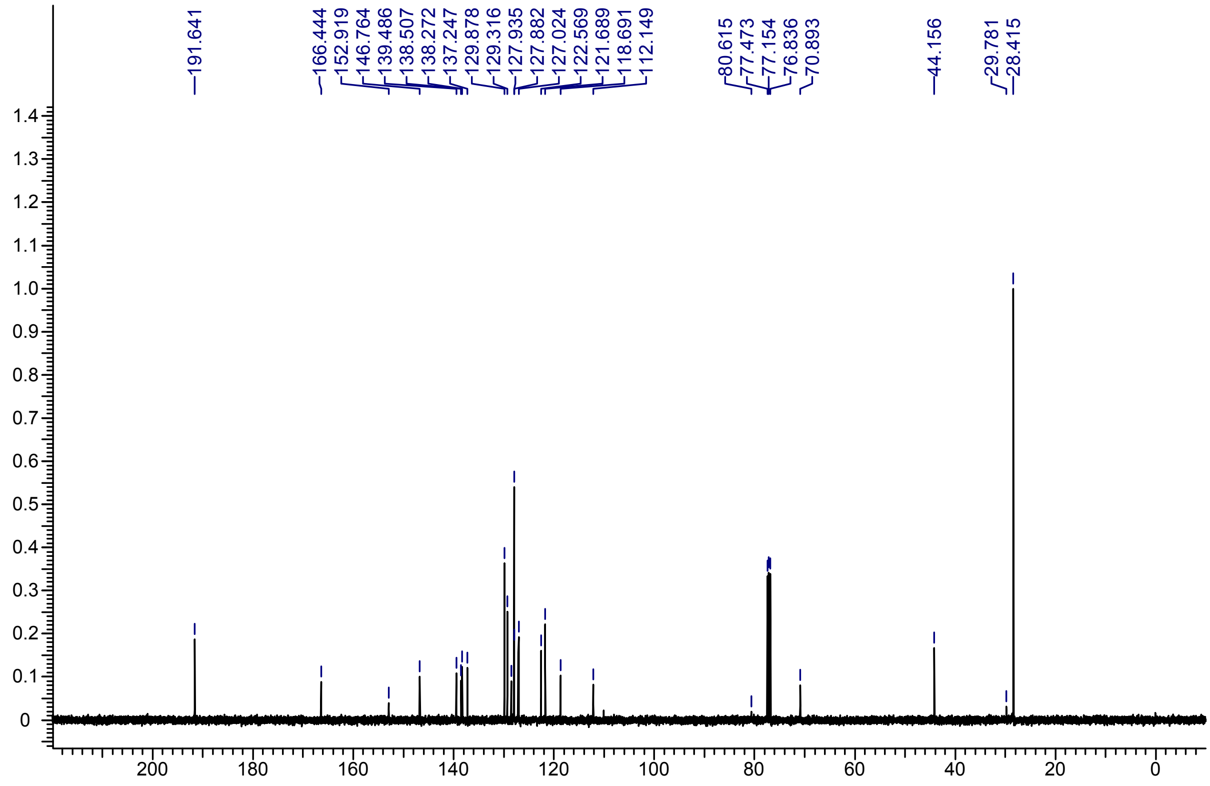
191.641
166.444
152.919
146.764
139.486
138.507
138.272
137.247
129.878
129.316
127.935
127.882
127.024
122.569
121.689
118.691
112.149
80.615
77.473
77.154
76.836
70.893
44.156
29.781
28.415

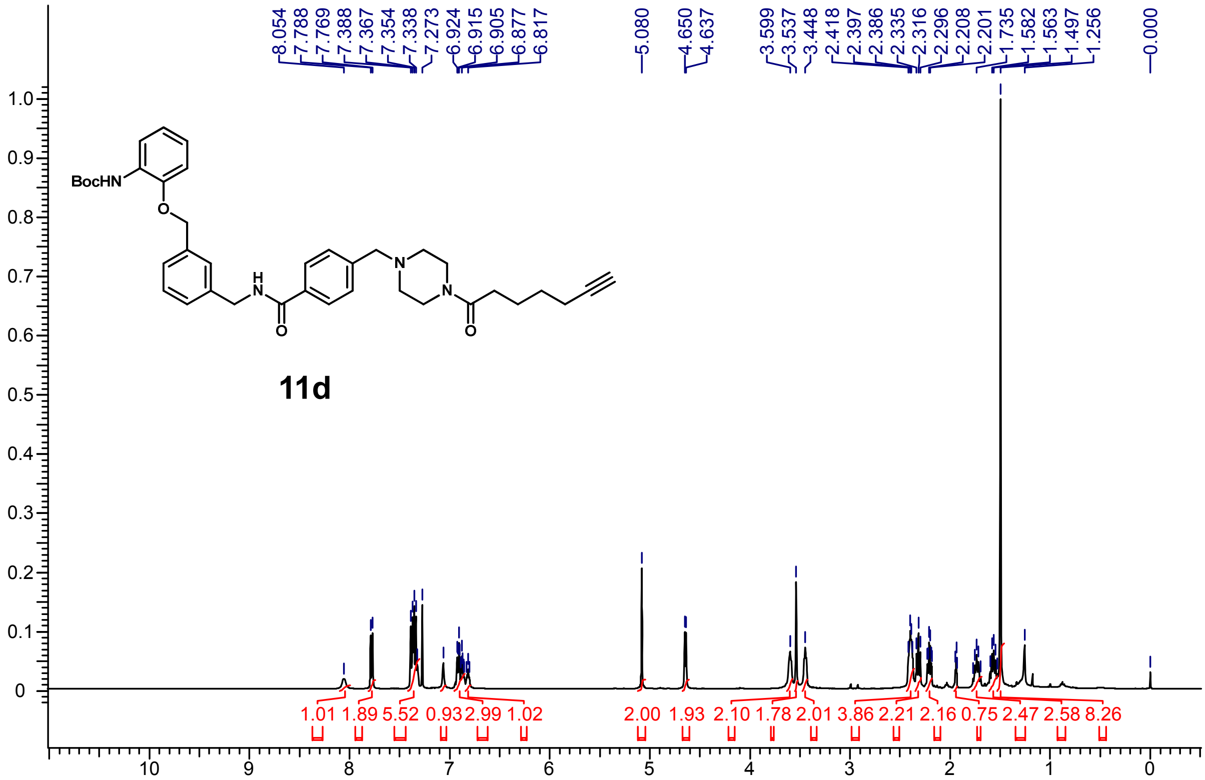
8.054 7.788 7.769 7.388 7.367 7.354 7.338 7.273 6.924 6.915 6.905 6.877 6.817
5.080
4.650 4.637
3.599 3.537 3.448 2.418 2.397 2.386 2.335 2.316 2.296 2.208 2.201 1.735 1.582 1.563 1.497 1.256
0.000
BocHN
O
H
N
N
N
O
O
11d
1.01 1.89 5.52 0.93 2.99 1.02 2.00 1.93 2.10 1.78 2.01 3.86 2.21 2.16 0.75 2.47 2.58 8.26

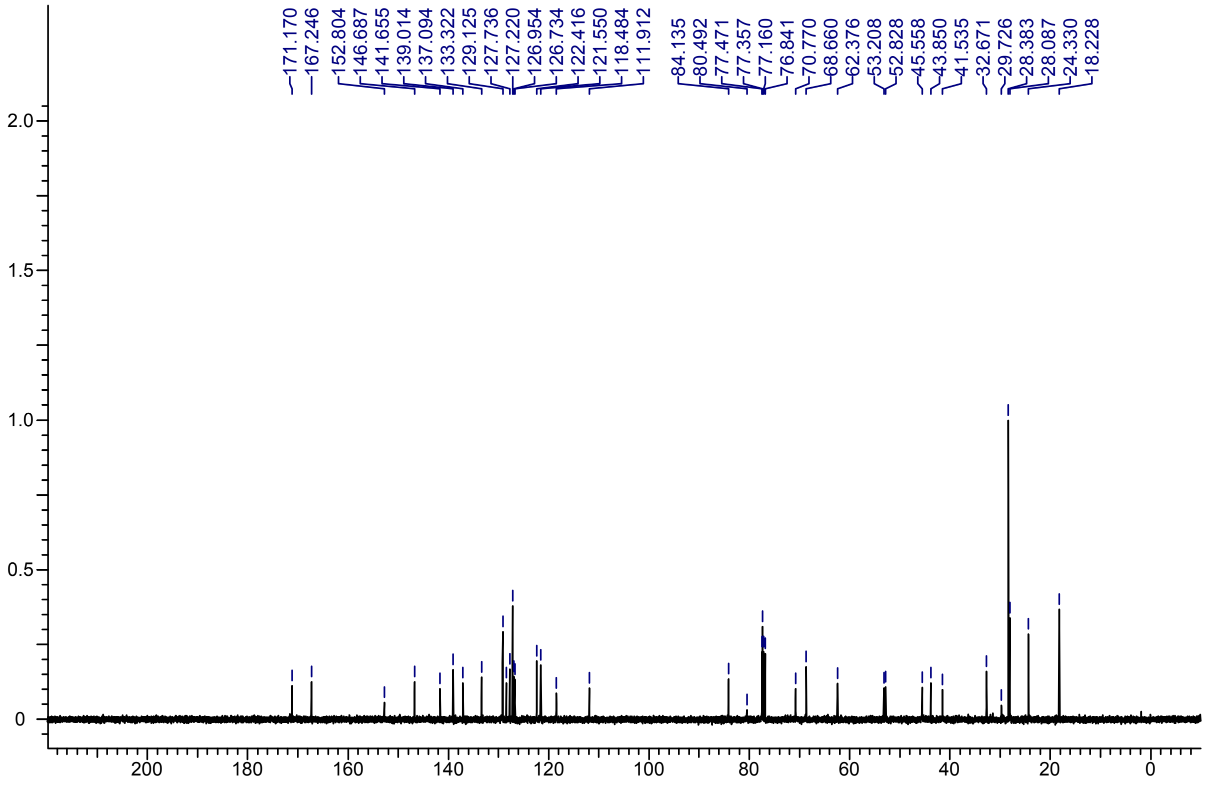
171.170 167.246 152.804 146.687 141.655 139.014 137.094 133.322 129.125 127.736 127.220 126.954 126.734 122.416 121.550 118.484 111.912
84.135 80.492 77.471 77.357 77.160 76.841 70.770 68.660 62.376 53.208 52.828 45.558 43.850 41.535 32.671 29.726 28.383 28.087 24.330 18.228

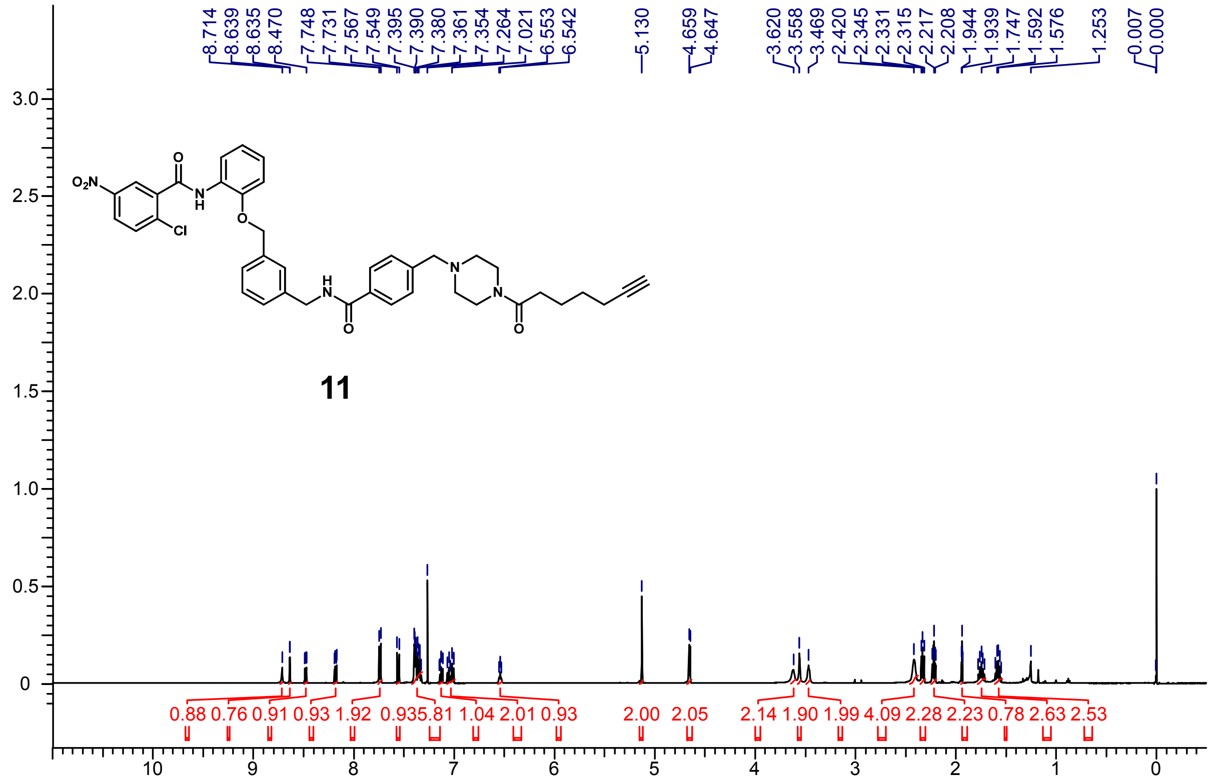

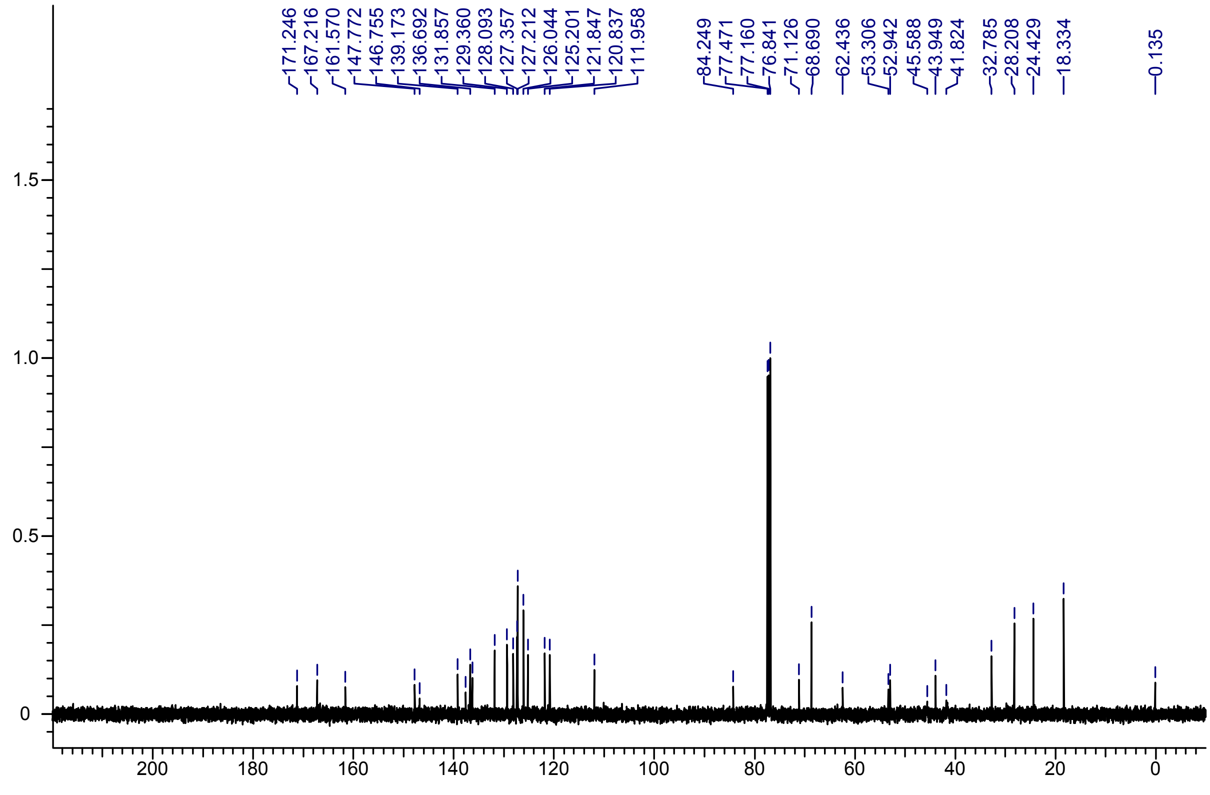

Supplement: Supplementary file 2 [file SC-007-C6SC01279E-s002.pdf]
